# Exploiting the radical reactivity of diazaphosphinanes in hydrodehalogenations and cascade cyclizations†

**DOI:** 10.1039/d0sc01352h

**Published:** 2020-04-23

**Authors:** Jingjing Zhang, Jin-Dong Yang, Jin-Pei Cheng

**Affiliations:** Center of Basic Molecular Science, Department of Chemistry, Tsinghua University Beijing 100084 China jdyang@mail.tsinghua.edu.cn jinpei_cheng@mail.tsinghua.edu.cn; State Key Laboratory of Elemento-Organic Chemistry, College of Chemistry, Nankai University Tianjin 300071 China

## Abstract

The remarkable reducibility of diazaphosphinanes has been extensively applied in various hydrogenations, based on and yet limited by their well-known hydridic reactivity. Here we exploited their unprecedented radical reactivity to implement hydrodehalogenations and cascade cyclizations originally inaccessible by hydride transfer. These reactions feature a broad substrate scope, high efficiency and simplicity of manipulation. Mechanistic studies suggested a radical chain process in which a phosphinyl radical is generated in a catalytic cycle *via* hydrogen-atom transfer from diazaphosphinanes. The radical reactivity of diazaphosphinanes disclosed here differs from their well-established hydridic reactivity, and hence, opens a new avenue for diazaphosphinane applications in organic syntheses.

## Abstract 2

Unprecedented radical reactivity of diazaphosphinanes was explored to implement efficient hydrodehalogenations and cascade cyclizations.

## Graphical abstract

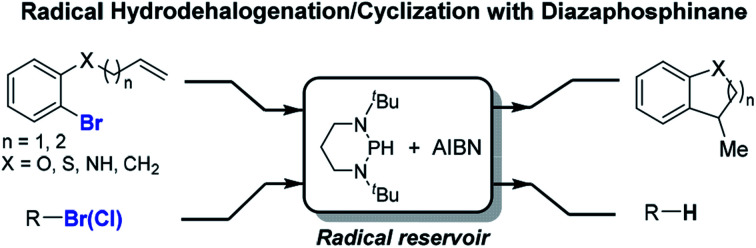

## Introduction

As is known, in polar hydride transfer (HT), an adequate thermodynamic compensation is needed to overcome its high kinetic barrier.^1^ Hence, direct HT usually requires a significant local accumulation of positive charge on hydride acceptors, which are commonly cationic species or some highly polarized compounds. This poses a very challenging, if not insuperable, barrier for reducing less polar substrates *via* HT. Consequently, hydrodehalogenation of organic halides with a hydride donor is barely achieved *via* nucleophilic aromatic substitution.^2^ Compared with polar HT, the radical pathway displays a kinetic superiority because of its considerably lower intrinsic barrier.^3^ In this regard, hydrogen-atom transfer (HAT) may provide an alternative to enable reduction with hydride donors, which is unlikely to occur through direct HT.

**Scheme 1:**
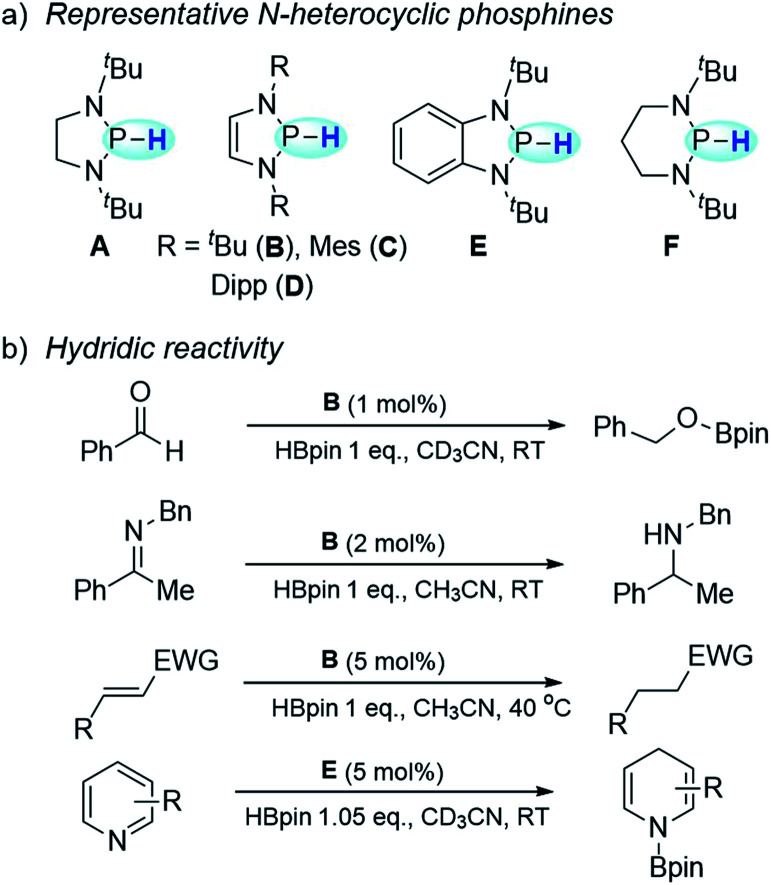
N-heterocyclic phosphines and their applications. (a) Representative N-heterocyclic phosphines. (b) Applications of N-heterocyclic phosphines in hydridic reduction.

N-heterocyclic phosphines have recently attracted particular attention owing to their superior hydricity (Scheme 1a).^4^ Their hydridic reactivity has been extensively exploited in catalytic or stoichiometric reduction of various unsaturated compounds, such as imines,^5^ aldehydes and ketones,^6^ polar olefins^7^ and pyridines^8^ (Scheme 1b). A kinetic scale with respect to the HT tendency from N-heterocyclic phosphines was also reported.^9^ In contrast to the well-studied hydridic reactivity, understanding of their reducibility in hydrogen-atom or electron transfer pathways remains strikingly underdeveloped.

**Scheme 2:**
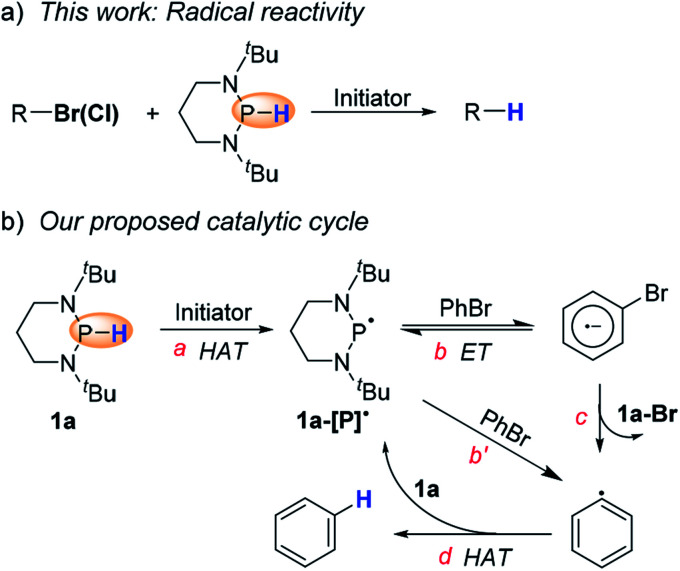
Our work. (a) Radical hydrodehalogenation, and (b) catalytic cycle proposed in this work.

Based on the hydrogen-atom donating ability of diazaphosphinanes (DAPs) derived recently,^10^ we envisioned that trivalent phosphinyl radicals, which could be generated *via* HAT from DAPs, may allow a chance for radical reduction, and hence, renders a quite different reactivity and selectivity pattern from that by direct HT of DAPs. Aiming to verify this assumption, we have examined here the hydrodehalogenation of organic halides under properly designed conditions in favor of radical initiation. To our delight, the anticipated radical hydrodehalogenation of organohalides was indeed realized with high efficiency and wide substrate coverage, and therefore, could be a superior alternative to substitute some of the traditional approaches (*e.g.*, halogen–metal exchange,^11^ transition metal catalysis,^12^ nucleophilic aromatic substitution^2^ and radical dehalogenation^13^) where limitation exists for removing halogens. Here, we used 1,3-di-*tert*-butyl-1,3,2-diazaphosphinane (**1a**, in Scheme 2a) as a potent hydrogen-atom donor to implement efficient radical hydrodehalogenation as well as cascade cyclization of organic halides, which were found to be inert when reduced by the same diazaphosphinane *via* direct HT.

Scheme 2b shows our design, which was proposed based on the following analysis. Comparing the P–H bond dissociation free energy (BDFE) (∼78 kcal mol^−1^)^10^ of **1a** with that of the isobutyronitrile α–C–H bond (∼85 kcal mol^−1^),^14^ we can expect a feasible HAT from **1a** to the isobutyronitrile radical, generated from homolysis of azobis(isobutyronitrile) (AIBN), to give the crucial phosphinyl radical **1a-[P]˙** (step a). The *in situ* formed **1a-[P]˙** with an oxidation potential (*E*_ox_) of −2.39 V (*vs.* ferrocene in acetonitrile, see the ESI† for details) is among the most potent neutral electron donors ever reported.^15^ It could undergo reversible electron transfer (ET, step b) with bromobenzene^16^ to give the reactive bromobenzene radical anion and stable phosphenium cation (or alternatively, abstracts a bromine atom (step b′) from bromobenzene). Then, subsequent spontaneous C–Br bond scission (step c) produces the phenyl radical (C–H BDFE of benzene: ∼105 kcal mol^−1^),^14^ which readily captures a hydrogen-atom from **1a** to regenerate **1a-[P]˙** (step d) and simultaneously initiate the next cycle.

## Results and discussion

To evaluate the feasibility of the present design, we chose the bromobenzene **2a** as the test substrate (Table 1). As seen, treatment of **2a** with 1.2 equiv. of **1a** and 10 mol% AIBN in toluene harvested the product benzene **3a** in 90% yield (entry 1 in Table 1). Reducing the amount of AIBN to 5 mol% caused an inferior result (78%, entry 2). Further evaluations were performed by varying P–H reductants. Replacement of **1a** with structurally similar **1b** resulted in a much lower yield (<10%, entry 3). This is primarily because the poor reducing power of the **1b**-derived phosphinyl radical (*E*_ox_ = −1.94 V, Fig. S1†) makes electron transfer to bromobenzene sluggish. The same reason could be applied to rationalize the poor results of **1c** and **1d** systems (<5%, entry 4 and 5). Nevertheless, employment of the stronger reducing reagent **C** only gave a moderate yield (77%, entry 6), along with some unidentified byproducts. This is probably due to the phosphinyl radical derived from **C** being too reactive to allow a sufficient ET (to bromobenzene) to proceed before it is quenched by other components in the system. In the control reactions without either the initiator AIBN or heating, trace amounts of products were obtained (<5%, entries 7 and 8), indicating that nucleophilic aromatic substitution may exist as a background reaction. Eventually, 1.2 equiv. of **1a** and 10 mol% AIBN in toluene solvent were used as the standard conditions.

**Table 1:** Condition optimization for hydrodehalogenation of bromobenzene

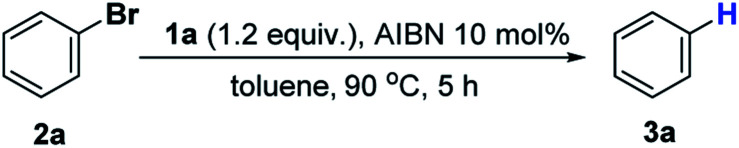		
Entry	Condition^a^	Yield^b^
1	Standard condition	90%
2	5 mol% AIBN	78%
3	**1b** as reductant	<10%
4	**1c** as reductant	<5%
5	**1d** as reductant	<5%
6	**C** as reductant	77%
7	No AIBN	<5%
8	No heat	<5%
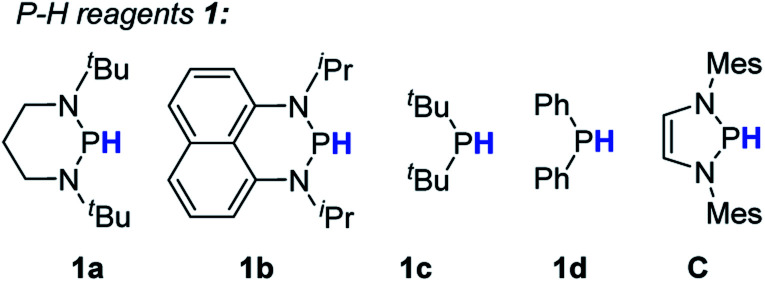		

a Reactions were conducted using 0.10 mmol of **2a** in 0.5 mL toluene.

b ^1^H NMR yields using 1,3,5-trimethoxybenzene as the internal standard.

**Scheme 3:**
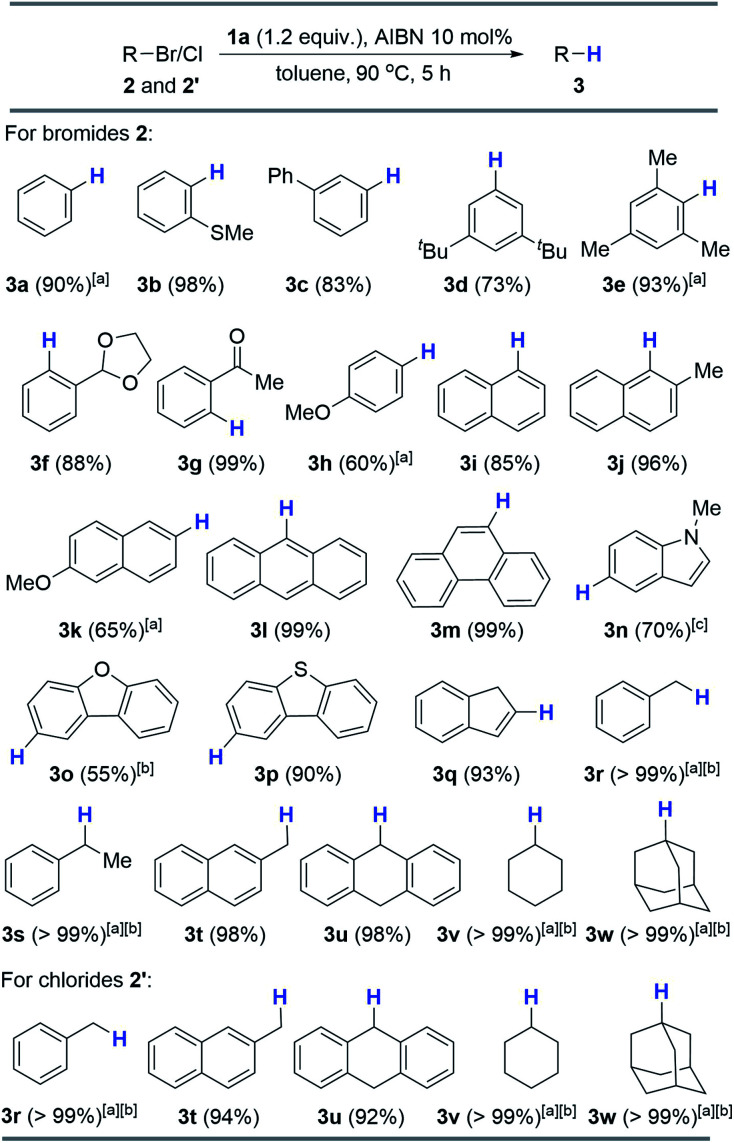
Substrate scope of hydrodebromination/dechlorination. The reactions were conducted using 0.10 mmol **2** or **2′** in 0.5 mL toluene and isolated yields were given unless otherwise specified. [a] ^1^H NMR yields using 1,3,5-trimethoxybenzene as the internal standard. [b] Using toluene-*d*_8_ as solvent for these low boiling products. [c] Reaction time: 12 hours.

With the optimized conditions derived, we next evaluated the substrate scope, which covered both aromatic and aliphatic bromides and chlorides (Scheme 3; see the ESI† for details). As shown in Scheme 3, the reduction of 2-methylthio-bromobenzene produced arene **3b** in a quantitative yield (98%). 3-Phenyl (**2c**) and 3,5-di-*tert*-butyl (**2d**) analogs provided good results (83% and 73%). Impressively, the sterically hindered 2,4,6-trimethyl-bromobenzene is also a compatible substrate, affording **3e** in 93% yield. The present reaction worked well with the labile acetal moiety and furnished **3f** in a high yield (88%). Notably, the 2-carbonyl analog can be quantitatively reduced to hydrodehalogenated **3g** (99%). Direct hydride transfer to the electrophilic carbonyl group, as previously reported,^6^ can be completely inhibited. As expected, a moderate yield (60%) was obtained for electron-rich 4-methoxy-bromobenzene (**2h**) because of its low reduction potential. Furthermore, hydrodebromination of substituted bromonaphthalenes (**2i–k**) proceeded smoothly and offered moderate to excellent yields (65–96%). Other condensed cyclic bromides (**2l** and **2m**) were also viable substrates which gave corresponding products quantitatively. Besides, electron-rich 5-bromoindole could react with **1a**/AIBN and **3n** was produced in a moderate yield (70%) after prolonging the reaction time to 12 hours. Other heterocyclic substrates can also be reduced to yield corresponding arenes (**3o** and **3p** in 55% and 90% yields, respectively). Alkenyl bromide can be efficiently hydrodehalogenated as well (**3q**, 93%). Additionally, all the alkyl bromides tested here were readily debrominated to give **3r–w** in excellent yields (>98%). Moreover, alkyl chlorides **2′** were also viable substrates, giving excellent yields under the standard conditions (Scheme 3).

**Scheme 4:**
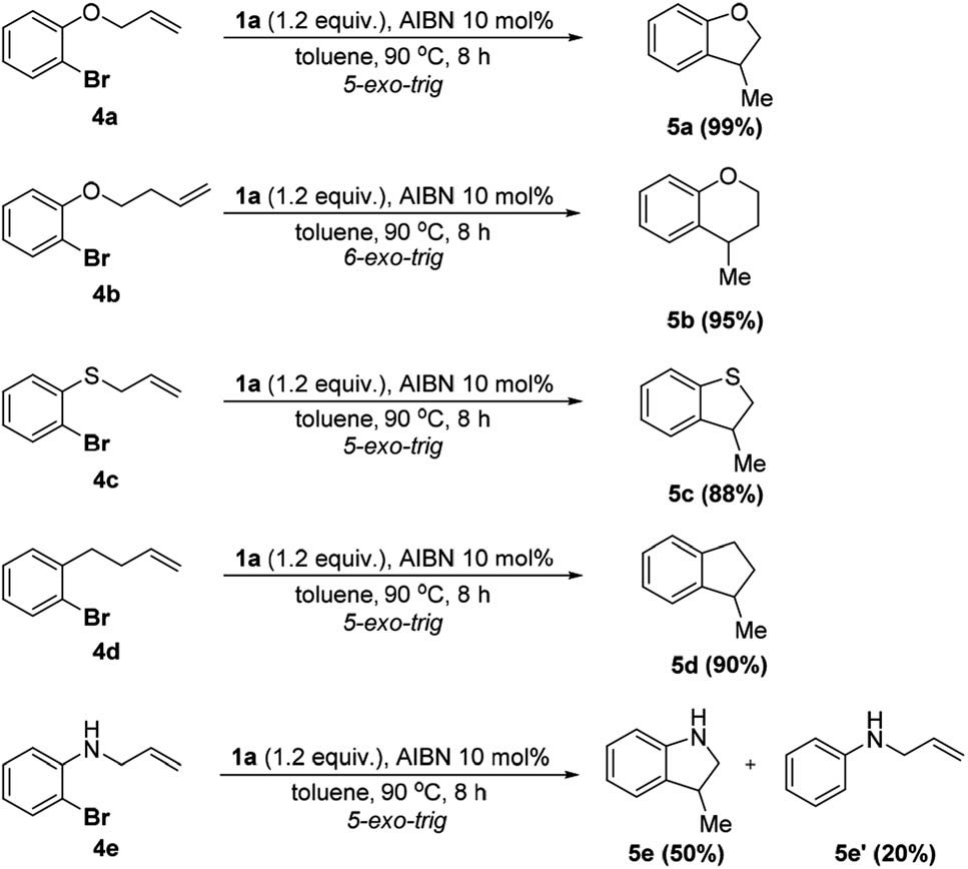
Reductive radical cyclization using 1.0 mmol of **4** in 3.0 mL toluene. Isolated yields were given.

To further apply the **1a**/AIBN system to realize a cascade cyclization, a series of substrates bearing *ortho*-allyl moieties were employed. The results are given in Scheme 4 (see the ESI† for details). As seen, **4a–d** were converted to the corresponding 5-*exo*-trig cyclization products in nearly quantitative yields (88–99%) and high chemo-selectivity (cyclization : direct hydrodehalogenation >15 : 1). Lengthening the side alkyl chain had a trivial effect on the reaction (**4b**, 95%). The excellent reactivity and chemoselectivity indicated that the present system could avoid the over reduction observed in other similar processes.^15*b*^ For the substrate bearing the NH group, both cyclization **5e** (50%) and direct hydrodehalogenation **5e′** (20%) products were observed with a recovery of about 20% of the starting material. Formation of **5e′** is presumably ascribed to the competitive HAT from the NH moiety or **1a** to the aryl radical.

Next, we focused on elucidating the mechanistic details of the present system with particular interest in the process of C–Br bond activation, *i.e.*, to identify whether the phosphinyl radical directly abstracts the bromine atom from bromobenzene like the reactions mediated by tin hydrides,^17^ silanes,^13*a*^ silylated cyclohexadienes,^13*b*^ and N-heterocyclic carbene–borane complexes,^13*c*,18^ or the phosphinyl radical transfers an electron to bromobenzene to trigger the cycle as depicted in Scheme 2b. DFT calculations showed that **1a-[P]˙** and **1b-[P]˙** should have a comparable ability (with an energy difference of 1.3 kcal mol^−1^, see the ESI† for details) in abstracting the bromine atom. This failed to explain the disparate yields of 90% for **1a-[P]˙** and <10% for **1b-[P]˙** (largely from a background reaction, see the text). Besides, according to the redox potentials of **1a-[P]˙** (*E*_ox_ = −2.39 V *vs.* Fc in MeCN) and bromobenzene (*E*_red_ = −2.8 V),^16^ the electron transfer from **1a-[P]˙** to bromobenzene is a feasible reversible process, while that for **1b-[P]˙** (*E*_ox_ = −1.94 V) is thermodynamically prohibited. These findings preferentially support an ET-initiated mechanism rather than a direct bromine abstraction.

**Scheme 5:**
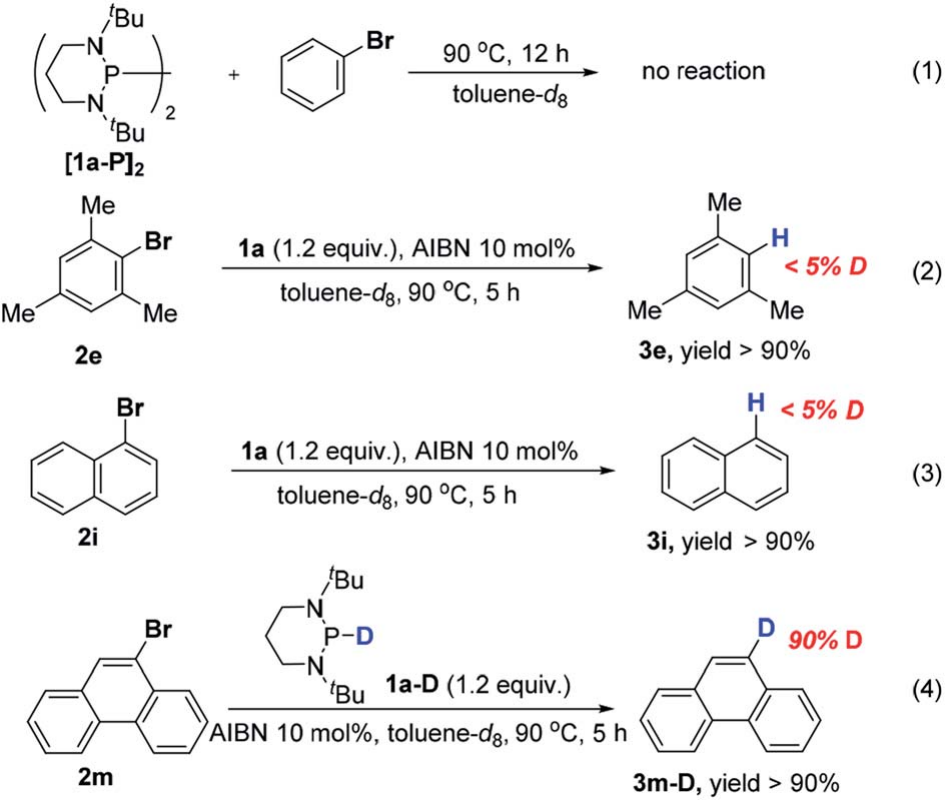
Control experiments with ^1^H NMR yields (using 1,3,5-trimethoxybenzene as the internal standard; see the ESI† for details).

On the other hand, we have recently found that **1a** reacted smoothly with AIBN to produce the bisphosphine **[1a-P]2**.^10^ Considering the facile interconversion between **[1a-P]2** and phosphinyl radical **1a-[P]˙**,^19^ bisphosphine was synthesized separately to examine whether it may have functioned as a radical reservoir during hydrodehalogenation. As depicted in Scheme 5 (eqn (1)), no product was detected when **[1a-P]2** was mixed with bromobenzene under the standard conditions. This is presumably because the relatively strong P–P bond of **[1a-P]2** made it difficult to dissociate to provide the corresponding phosphinyl radical **1a-[P]˙** at the reaction temperature (90 °C, see the ESI† for details). Thus, the possibility of **[1a-P]2** as a reaction intermediate was excluded. Because the solvent toluene could be a common hydrogen donor, and if so, it has potential to quench the phenyl radical. To examine this, deuterium labelling experiments were conducted in toluene-*d*_8_ (eqn (2) and (3)). The absence of deuterium incorporation indicated that the hydrogen could not come from toluene. Furthermore, replacement of **1a** with its deuterated counterparts **1a–d** resulted in the desired product with a 90% deuterium abundance (eqn (4)). Hence, **1a** is suggested to be the hydrogen source at last.

Based on these control experiments, the plausible catalytic mechanism proposed in Scheme 2 can be verified. The *in situ* generated phosphinyl radical preferentially served as a potent electron donor to activate the bromides. The reaction is most likely to proceed through an ET-initiated radical chain process,^20^ although a direct bromine abstraction cannot be completely excluded at the present stage.

## Conclusions

In conclusion, in this work we unlocked unprecedented radical reactivity of diazaphosphinanes to achieve efficient hydrodehalogenation and cascade cyclization, which is distinguished from their well-established hydridic reactivity. The phosphinyl radical, accessed in a catalytic cycle, is believed to be responsible for activating the carbon–bromine bonds through ET. This new reaction may provide a superior approach to remove halogens from many organohalides in terms of substrate scope, reaction efficiency and chemo-selectivity. Exploitation of other radical reductions using the strategy presented here is ongoing.

## Conflicts of interest

There are no conflicts to declare.

## Supplementary Material

SC-011-D0SC01352H-s001
