# Supplementary material for: Exploiting the radical reactivity of diazaphosphinanes in hydrodehalogenations and cascade cyclizations
Source: Chem Sci. 2020 Apr 23;11(18):4786–90. doi: 10.1039/d0sc01352h (PMC8159257; doi:10.1039/d0sc01352h)
Supplement: SC-011-D0SC01352H-s001 [file SC-011-D0SC01352H-s001.pdf]

Electronic Supplementary Material (ESI) for Chemical Science.
This journal is © The Royal Society of Chemistry 2020

Supplementary Information

Exploiting the Radical Reactivity of Diazaphosphinanes in Hydrodehalogenations and Cascade Cyclizations

Jingjing Zhang,[a] Jin-Dong Yang,[a*] Jin-Pei Cheng[a,b*]

[a] Center of Basic Molecular Science, Department of Chemistry, Tsinghua University, Beijing 100084, China.
[b] State Key Laboratory of Elemento-organic Chemistry, College of Chemistry, Nankai University, Tianjin 300071, China.

Content

1. General information. S2
2. Preparation of 1,3-Di-tert-butyl-1,3,2-diazaphosphinane **1a**. S2
3. The Synthesis of **1a-[P]**$^+$ and **1b-[P]**$^+$. S3
4. Cyclic Voltammetry of the Phosphenium Cations of **1a** and **1b**. S3
5. General Procedure for Condition Optimization for Hydrodehalogenation of Bromobenzene. S3
6. General Procedure for Hydrodehalogenations of Aryl, Alkenyl, Alkyl Bromides **2** and chlorides **2'**. S4
7. General Procedure for Cyclizations. S5
8. Mechanism Studies in Scheme 5. .... S7
9. DFT Calculations. S11
10. Representative NMR Spectra. S13
11. SMD-M06-2X/6-31+G(d) Calculated Cartesian Coordinates and Energies. S33
12. Reference. S42

1. General information.

Chemicals: All hydrodehalogenation substrates **2** and **2'** were purchased from J&K Chemical. Other reagents and solvent were purchased from **J&K** or **TCI** Chemicals and used without further purification unless specified otherwise. **4a-e** were synthesized according to reported methods.[1] **1a** has been synthesized and characterized in our paper.[2] And **1a-D**, **1b**, **1a-[P]$^+$** and **1b-[P]$^+$** have been synthesized and characterized in our recent paper,[3] herein we only showed NMR spectra of **1a-[P]$^+$** and **1b-[P]$^+$** to clarify the reliability. Toluene and toluede-d_8 were purchased from **J&K** Chemical (99.9 %, Extra dry, water < 10 ppm, J&K seal). Reaction temperature refers to the temperature of an aluminum heating block or a silicon oil bath, which was controlled by an electronic temperature modulator from IKA.

Reactions: All hydrodehalogenation and cyclization reactions were carried out in dry glass wares under an argon atmosphere using Schlenk technique throughout the reaction procedures.

Analytics: ^{1}H and ^{13}C NMR spectra were recorded in $CDCl_3$ (*δ* = 7.26 for ^{1}H NMR and *δ* = 77.16 for ^{13}C NMR) on 400 MHz NMR instrument at Center of Basic Molecular Science (CBMS) of Tsinghua University. Data for ^{1}H NMR spectra are reported as follows: chemical shift (multiplicity, coupling constants, number of hydrogens). Abbreviations are as follows: s (singlet), d (doublet), t (triplet), q (quartet), m (multiplet) and br (broad).

Electrochemistry: All the samples were prepared and all the electrochemical experiments were performed in an inert Ar atmosphere. The supporting electrolyte was $[Bu_4N][PF_6]$, which was recrystallized three times in EtOH and dried about 12 hours before use, and the electrolyte concentration is about 0.1 M in acetonitrile. A standard three-electrode cell consists of a glassy carbon disk as work electrode, a platinum wire as a counter electrode, and 0.1 M $AgNO_3$/Ag (in 0.1 M $[Bu_4N][PF_6]$-acetonitrile) as reference electrode. Ferrocene ($Fc^{+/0}$) was used as an external reference and was found to be 0.04 V with respect to our reference electrode. The sample concentrations of **1a-[P]$^+$** and **1b-[P]$^+$** are about 1.0 mM. The scan rate was 100 mV/s. The potentials are reported in volts (V) *vs.* $Fc^{+/0}$.

2. Preparation of 1,3-Di-tert-butyl-1,3,2-diazaphosphinane 1a.

The 1,3-di-tert-butyl-1,3,2-diazaphosphinane **1a** was prepared and characterized in our recent paper.[2]

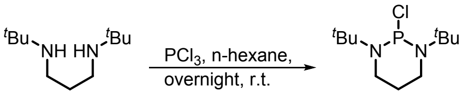

1a-Cl

3.3 mL (5.0 g, 37 mmol) of phosphorus trichloride was treated dropwise with a solution of 13.0 g (70 mmol) $(CH_2)_3(NH^tBu)_2$ in 200 mL of n-hexane under stirring at 0 °C. After stirring overnight, the reaction mixture was filtered. Hexane was removed from the filtrate in vacuum to produce yellow solid, which was then washed with Et_2O (3 ×10 mL) to give 6.6 g yellow solid of **1a-Cl** (71%).[2]

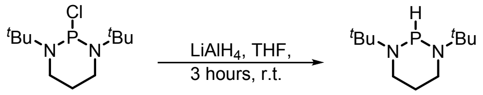

1a-Cl **1a**

A solution of **1a-Cl** (2.5 g, 10 mmol) in THF (40 mL) was cooled to 0 °C, and a 1 M solution of $LiAlH_4$ in THF (2.5 mL, 2.5 mmol) was slowly added. The mixture was stirred for 3 hours at room temperature. The

solvent was evaporated in vacuum. Then, the residue was extracted with n-hexane (50 mL) and filtered. The solvent of filtrate was removed and produced 1.9 g yellow oil of **1a** (88%).[2]

3. The Synthesis of 1a-[P]$^+$ and 1b-[P]$^+$.[3]

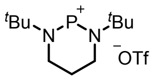

1a-[P]$^+$

The preparation of **1a-[P]$^+$** was the same as **1b-[P]$^+$**. $AgSO_3CF_3$ (AgOTf) (0.51 g, 2.0 mmol) was added into the solution of 1,3-di-tert-butyl-2-chloro-1,3,2-diazaphosphinane[2] (0.50 g, 2.0 mmol) in 10 mL of acetonitrile. After 1 hour stirring, the reaction mixture was filtered through a glass frit packed with Celite. The filtrate was concentrated to yield white solid **1a-[P]$^+$** 0.67 g (92%).

^{1}H NMR (400 MHz, CD_3CN) δ 3.39 (dd, *J* = 11.3, 5.8 Hz, 4H), 2.10 – 1.99 (m, 2H), 1.45 (d, *J* = 3.1 Hz, 18H). **^{13}C NMR** (101 MHz, CD_3CN) δ 62.63 (d, *J* = 18.7 Hz), 43.98 (d, *J* = 7.8 Hz), 28.52 (d, *J* = 14.6 Hz), 24.43. **^{31}P NMR** (162 MHz, CD_3CN) δ 248.59 (s). **ESI-HR** calcd for $C_{11}H_{24}N_2P$ (M^+) 215.1672, found 215.1667.

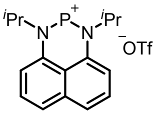

1b-[P]$^+$

$AgSO_3CF_3$ (AgOTf) (0.51 g, 2.0 mmol) was added into the solution of 2-chloro-1,3-diisopropyl-2,3-dihydro-1H-naphtho[1,8-*de*][1,3,2]diazaphosphinine[4] (0.61 g, 2.0 mmol) in 10 mL of acetonitrile. After 1 hour stirring, the reaction mixture was filtered through a glass frit packed with Celite. The filtrate was concentrated to yield red solid **1b-[P]$^+$** 0.78 g (93%).

^{1}H NMR (400 MHz, CD_3CN) δ 7.47 – 7.33 (m, 4H), 6.94 (dd, *J* = 5.7, 2.9 Hz, 2H), 4.40 – 4.31 (m, *J*, 2H), 1.54 (dd, *J* = 6.7, 1.1 Hz, 12H). **^{31}P NMR** (162 MHz, CD_3CN) δ 111.47 (s). **^{13}C NMR** (101 MHz, CD_3CN) δ 137.22, 135.78, 126.75, 121.43, 119.48, 108.95, 51.13 (d, *J* = 27.6 Hz), 21.33 (d, *J* = 17.5 Hz). **^{1}H NMR** (400 MHz, toluene-d_8) δ 7.19 (d, *J* = 8.0 Hz, 2H), 7.12 – 7.08 (m, 2H), 6.51 (d, *J* = 7.7 Hz, 2H), 3.89 (tt, *J* = 13.5, 6.6 Hz, 2H), 1.32 (dd, *J* = 6.6, 2.2 Hz, 12H). **^{31}P NMR** (162 MHz, toluene-d_8) δ 141.26 (s).
The NMR spectroscopic data are in good agreement with those in the literature.[4a]

4. Cyclic Voltammetry of the Phosphenium Cations of 1a and 1b.[3]

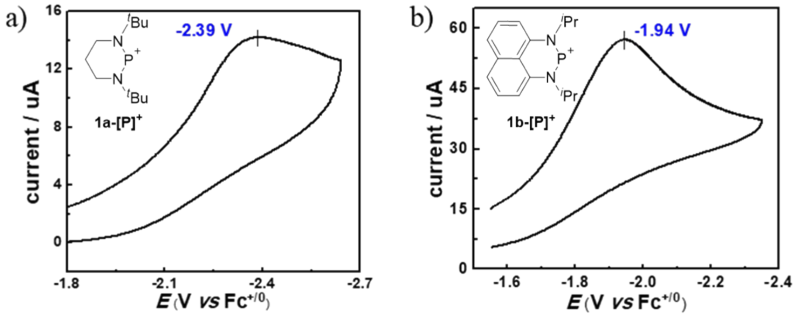

Figure S1. Cyclic voltammetry of the phosphenium cations of a) **1a** and b) **1b** in acetonitrile at 20 °C with 0.1 M $[Bu_4N][PF_6]$ as supporting electrolyte at a sweep rate of 100 mV/s. The concentrations of the phosphenium cations are about 1.0 mM in acetonitrile.

5. General Procedure for Condition Optimization for Hydrodehalogenation of Bromobenzene.

2a (0.1 mmol), AIBN, reductants (0.12 mmol) and toluene-d_8 (0.5 mL) were taken in a Schlenk tube under argon. The mixture was stirred at 90 °C for 5 hours to ensure complete hydrodehalogenation. The resulting mixture was cooling down to room temperature and 1,3,5-trimethoxybenzene as the internal standard was

added to the mixture (0.1 mmol). The yields were monitored by ^{1}H NMR spectra.

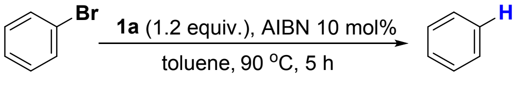

2a **3a**

Entry	Condition[a]	Yield[b]
1	standard condition	90%
2	5 mol% AIBN	78%
3	**1b** as reductant	< 10%
4	**1c** as reductant	< 5%
5	**1d** as reductant	< 5%
6	**C** as reductant	77%
7	no AIBN	< 5%
8	no heat	< 5%

*P-H reagents **1**:*

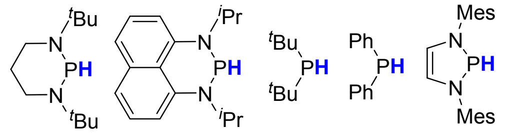

1a **1b** **1c** **1d** **C**

[a] Reactions were conducted using 0.10 mmol of **2a** in 0.5 mL toluene. [b] ^{1}H NMR yields using 1,3,5-trimethoxybenzene as the internal standard.

6. General Procedure for Hydrodehalogenations of Aryl, Alkenyl, Alkyl Bromides 2 and chlorides 2'.

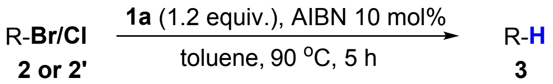

2 or **2'** (0.1 mmol), AIBN (0.01 mmol), **1a** (0.12 mmol) and toluene (0.5 mL) were taken in a Schlenk tube under argon. The mixture was stirred at 90 °C for 5 hours to ensure complete hydrodehalogenation. The resulting mixture was concentrated under vacuum and the crude product was purified by flash column chromatography through a silica plug using hexane as the eluent.

Representative NMR spectra data for products **3**:

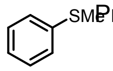

3b

Prepared from **2b** (0.1 mmol) for 5 hours at 90 °C. Purification by flash column chromatography (silica gel, hexane) gave **3b** in 98% yield as the colorless oil. **^{1}H NMR** (400 MHz, $CDCl_3$) δ 7.39 – 7.25 (m, 4H), 7.18 (ddd, J = 8.3, 4.0, 1.7 Hz, 1H), 2.52 (s, 3H). **^{13}C NMR** (101 MHz, $CDCl_3$) δ 138.46, 128.83, 126.71, 125.05, 15.90.

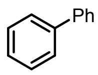

3c

Prepared from **2c** (0.1 mmol) for 5 hours at 90 °C. Purification by flash column chromatography (silica gel, hexane) gave **3c** in 83% yield as the white solid. **^{1}H NMR** (400 MHz, $CDCl_3$) δ 7.69 (d, J = 7.6 Hz, 4H), 7.53 (t, J = 7.7 Hz, 4H), 7.47 – 7.40 (m, 2H). **^{13}C NMR** (101 MHz, $CDCl_3$) δ 141.33, 128.82, 127.32, 127.24.

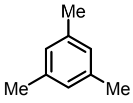

3e

Prepared from **2e** (0.1 mmol) for 5 hours at 90 °C. Purification by flash column chromatography (silica gel, hexane) gave **3e** in 93% yield as the colorless oil. **^{1}H NMR** (400 MHz, $CDCl_3$) δ 6.91 (s, 3H), 2.39 (s, 9H). **^{13}C NMR** (101 MHz, $CDCl_3$) δ 137.76, 126.98,

21.25.

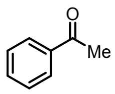

3g

Prepared from **2g** (0.1 mmol) for 5 hours at 90 °C. Purification by flash column chromatography (silica gel, hexane) gave **3g** in 99% yield as the colorless oil. **^{1}H NMR** (400 MHz, $CDCl_3$) δ 8.02 – 7.92 (m, 2H), 7.59 (t, *J* = 7.4 Hz, 1H), 7.49 (t, *J* = 7.6 Hz, 2H), 2.63 (s, 3H). **^{13}C NMR** (101 MHz, $CDCl_3$) δ 198.12, 137.16, 133.08, 128.56, 128.30, 26.59.

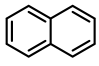

3i

Prepared from **2i** (0.1 mmol) for 5 hours at 90 °C. Purification by flash column chromatography (silica gel, hexane) gave **3i** in 85% yield as the colorless oil. **^{1}H NMR** (400 MHz, $CDCl_3$) δ 7.89 (dd, *J* = 6.1, 3.3 Hz, 4H), 7.52 (dd, *J* = 6.2, 3.2 Hz, 4H). **^{13}C NMR** (101 MHz, $CDCl_3$) δ 133.52, 127.93, 125.86.

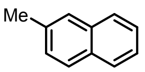

3j

Prepared from **2j** (0.1 mmol) for 5 hours at 90 °C. Purification by flash column chromatography (silica gel, hexane) gave **3j** in 96% yield as the colorless oil. **^{1}H NMR** (400 MHz, $CDCl_3$) δ 7.85 (d, *J* = 7.9 Hz, 1H), 7.81 – 7.79 (m, 2H), 7.66 (s, 1H), 7.47 (qd, *J* = 6.8, 3.4 Hz, 2H), 7.37 (dd, *J* = 8.4, 1.2 Hz, 1H), 2.57 (s, 3H). **^{13}C NMR** (101 MHz, $CDCl_3$) δ 135.45, 133.71, 131.74, 128.13, 127.71, 127.62, 127.25, 126.85, 125.87, 124.96, 21.73.

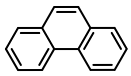

3m

Prepared from **2m** (0.1 mmol) for 5 hours at 90 °C. Purification by flash column chromatography (silica gel, hexane) gave **3m** in 99% yield as the white solid. **^{1}H NMR** (400 MHz, $CDCl_3$) δ 8.73 (d, *J* = 8.1 Hz, 2H), 7.93 (dd, *J* = 7.8, 0.9 Hz, 2H), 7.78 (s, 2H), 7.74 – 7.61 (m, 4H). **^{13}C NMR** (101 MHz, $CDCl_3$) δ 132.07, 130.33, 128.58, 126.93, 126.57, 122.67.

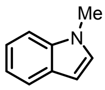

3n

Prepared from **2n** (0.1 mmol) for 12 hours at 90 °C. Purification by flash column chromatography (silica gel, hexane) gave **3n** in 70% yield as the colorless oil. **^{1}H NMR** (400 MHz, $CDCl_3$) δ 7.70 (d, *J* = 7.1 Hz, 1H), 7.38 (d, *J* = 7.7 Hz, 1H), 7.33 – 7.24 (m, 1H), 7.17 (td, *J* = 6.9, 0.8 Hz, 1H), 7.10 (d, *J* = 2.9 Hz, 1H), 6.55 (d, *J* = 3.0 Hz, 1H), 3.84 (s, 3H). **^{13}C NMR** (101 MHz, $CDCl_3$) δ 136.72, 128.78, 128.51, 121.49, 120.88, 119.28, 109.18, 100.92, 32.81.

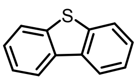

3p

Prepared from **2p** (0.1 mmol) for 5 hours at 90 °C. Purification by flash column chromatography (silica gel, hexane) gave **3p** in 90% yield as the colorless oil. **^{1}H NMR** (400 MHz, $CDCl_3$) δ 8.25 – 8.16 (m, 2H), 7.97 – 7.86 (m, 2H), 7.57 – 7.41 (m, 4H). **^{13}C NMR** (101 MHz, $CDCl_3$) δ 139.47, 135.58, 126.72, 124.37, 122.83, 121.60.

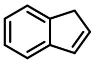

3q

Prepared from **2q** (0.1 mmol) for 5 hours at 90 °C. Purification by flash column chromatography (silica gel, hexane) gave **3q** in 93% yield as the colorless oil. **^{1}H NMR** (400 MHz, $CDCl_3$) δ 7.63 (dd, *J* = 22.7, 7.3 Hz, 2H), 7.47 (t, *J* = 7.3 Hz, 1H), 7.38 (t, *J* = 7.2 Hz, 1H), 7.07 (d, *J* = 5.4 Hz, 1H), 6.77 – 6.64 (m, 1H), 3.56 (s, 2H). **^{13}C NMR** (101 MHz, $CDCl_3$) δ 145.01, 143.82, 134.25, 132.24, 126.40, 124.72, 123.87, 121.12, 39.21.

7. General Procedure for Cyclizations.

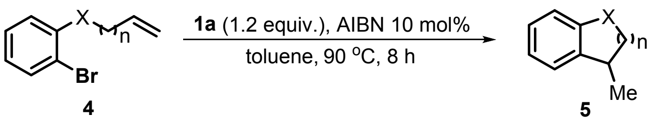

4 (1.0 mmol), AIBN (0.1 mmol), **1a** (1.2 mmol) and toluene (3.0 mL) were taken in a Schlenk tube under argon. The mixture was stirred at 90 °C for 8 hours. The resulting mixture was concentrated under vacuum and the crude product was purified by flash column chromatography through a silica plug with hexane as the eluent.

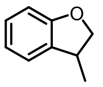

5a

Prepared from **4a** (1.0 mmol) for 8 hours at 90 °C. Purification by flash column chromatography (silica gel, hexane) gave **5a** in 99% yield as the colorless oil. ^{1}**H NMR** (400 MHz, $CDCl_3$) δ 7.19 – 7.06 (m, 2H), 6.86 (t, *J* = 7.4 Hz, 1H), 6.78 (d, *J* = 8.0 Hz, 1H), 4.69 – 4.65 (m, 1H), 4.11 – 4.00 (m, 1H), 3.63 – 3.42 (m, 1H), 1.33 (d, *J* = 6.9 Hz, 3H). ^{13}**C NMR** (101 MHz, $CDCl_3$) δ 159.73, 132.26, 127.99, 123.79, 120.43, 109.47, 78.47, 36.50, 19.31.

The NMR spectroscopic data are in good agreement with those in the literature.[5]

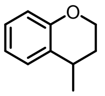

5b

Prepared from **4b** (1.0 mmol) for 8 hours at 90 °C. Purification by flash column chromatography (silica gel, hexane) gave **5b** in 95% yield as the colorless oil. ^{1}**H NMR** (400 MHz, $CDCl_3$) δ 7.15 (d, J = 7.7 Hz, 1H), 7.10 – 7.04 (m, 1H), 6.89 – 6.82 (m, 1H), 6.82 – 6.75 (m, 1H), 4.18 (qdd, *J* = 10.8, 7.2, 3.3 Hz, 2H), 2.95 (dd, *J* = 13.2, 6.6 Hz, 1H), 2.12 – 2.04 (m, 1H), 1.80 – 1.64 (m, 1H), 1.33 (d, *J* = 7.0 Hz, 3H). ^{13}**C NMR** (101 MHz, $CDCl_3$) δ 154.34, 128.64, 127.62, 127.21, 120.20, 116.72, 63.86, 30.34, 28.51, 22.20.

The NMR spectroscopic data are in good agreement with those in the literature.[6]

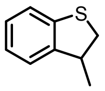

5c

Prepared from **4c** (1.0 mmol) for 8 hours at 90 °C. Purification by flash column chromatography (silica gel, hexane) gave **5c** in 88% yield as the colorless oil. ^{1}**H NMR** (400 MHz, $CDCl_3$) δ 7.11 (d, *J* = 7.5 Hz, 1H), 7.05 – 6.99 (m, 2H), 6.95 (t, *J* = 7.4 Hz, 1H), 3.46 – 3.34 (m, 2H), 2.89 (dd, *J* = 10.2, 8.1 Hz, 1H), 1.28 (d, *J* = 6.6 Hz, 3H). ^{13}**C NMR** (101 MHz, $CDCl_3$) δ 144.40, 141.29, 127.39, 124.27, 123.68, 122.28, 42.75, 40.89, 18.90.

The NMR spectroscopic data are in good agreement with those in the literature.[7]

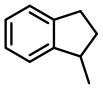

5d

Prepared from **4d** (1.0 mmol) for 8 hours at 90 °C. Purification by flash column chromatography (silica gel, hexane) gave **5d** in 90% yield as the colorless oil. ^{1}**H NMR** (400 MHz, $CDCl_3$) δ 7.23 – 7.10 (m, 4H), 3.21 – 3.15 (m, 1H), 2.95 – 2.75 (m, 2H), 2.39 – 2.22 (m, 1H), 1.64 – 1.52 (m, 1H), 1.29 (d, J = 6.9 Hz, 3H). ^{13}**C NMR** (101 MHz, $CDCl_3$) δ 148.73, 143.85, 126.09, 126.07, 124.31, 123.15, 39.42, 34.74, 31.44, 19.87.

The NMR spectroscopic data are in good agreement with those in the literature.[8]

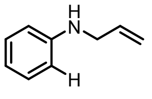

5e`

Prepared from **4e** (1.0 mmol) for 8 hours at 90 °C. Purification by flash column chromatography (silica gel, hexane / ethyl acetate = 50 : 1) gave **5e'** in 20% yield as the colorless oil. ^{1}**H NMR** (400 MHz, $CDCl_3$) δ 7.17 (t, *J* = 7.7 Hz, 2H), 6.70 (t, *J* = 7.4 Hz, 1H), 6.62 (d, *J* = 8.2 Hz, 2H), 5.95 – 5.92 (m, 1H), 5.28 (d, *J* = 17.2 Hz, 1H), 5.16 (d, *J* = 10.2 Hz, 1H), 3.77 (d, *J* = 5.3 Hz, 2H). ^{13}**C NMR** (101 MHz, $CDCl_3$) δ 148.09, 135.50, 129.23, 117.54, 116.22, 113.00, 46.58.

The NMR spectroscopic data are in good agreement with those in the literature.[9]

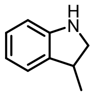

5e

Prepared from **4e** (1.0 mmol) for 8 hours at 90 °C. Purification by flash column chromatography (silica gel, hexane / ethyl acetate = 50 : 1) gave **5e** in 50% yield as the colorless oil. **^{1}H NMR** (400 MHz, $CDCl_3$) δ 7.08 (d, *J* = 7.3 Hz, 1H), 7.02 (t, *J* = 7.6 Hz, 1H), 6.73 (t, *J* = 7.4 Hz, 1H), 6.64 (t, *J* = 7.7 Hz, 1H), 3.68 (t, *J* = 8.6 Hz, 1H), 3.43 – 3.29 (m, 1H), 3.10 (t, *J* = 8.6 Hz, 1H), 1.31 (d, *J* = 6.8 Hz, 3H). **^{13}C NMR** (101 MHz, $CDCl_3$) δ 151.23, 134.35, 127.28, 123.36, 118.69, 109.50, 55.45, 36.66, 18.66.

The NMR spectroscopic data are in good agreement with those in the literature.[10]

8. Mechanism Studies in Scheme 5.

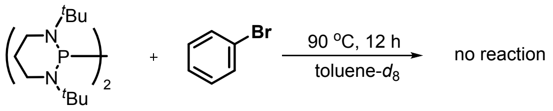

AIBN (0.12 mmol), **1a** (0.1 mmol) and toluene-d_8 (0.5 mL) were mixed in a Schlenk tube under argon. The solution reacted at 90 °C for 2 hours. The dimer **[1a-P]$_2$** was quantitatively generated through the ^{31}P NMR analysis,[3] and then the bromobenzene (0.1 mmol) was added into the **[1a-P]$_2$** solution. The new mixture continued to react at 90 °C for 12 hours. No desired debrominated product was detected.

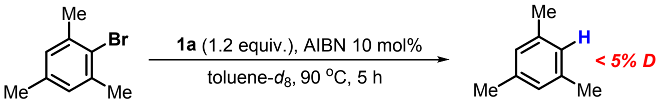

2e (0.1 mmol), AIBN (0.01 mmol), **1a** (0.12 mmol), internal standard (1,3,5-trimethoxybenzene, 0.06 mmol) and toluene-d_8 (0.5 mL) were taken in a NMR tube under argon at 90 °C for 5 hours. The deuterated ratio of the product **3e** was determined by in situ ^{1}H NMR spectrum in toluene-d_8. And the NMR spectra of mixture for the reaction were shown as follow:

(1) ^{1}H NMR in toluene-d_8

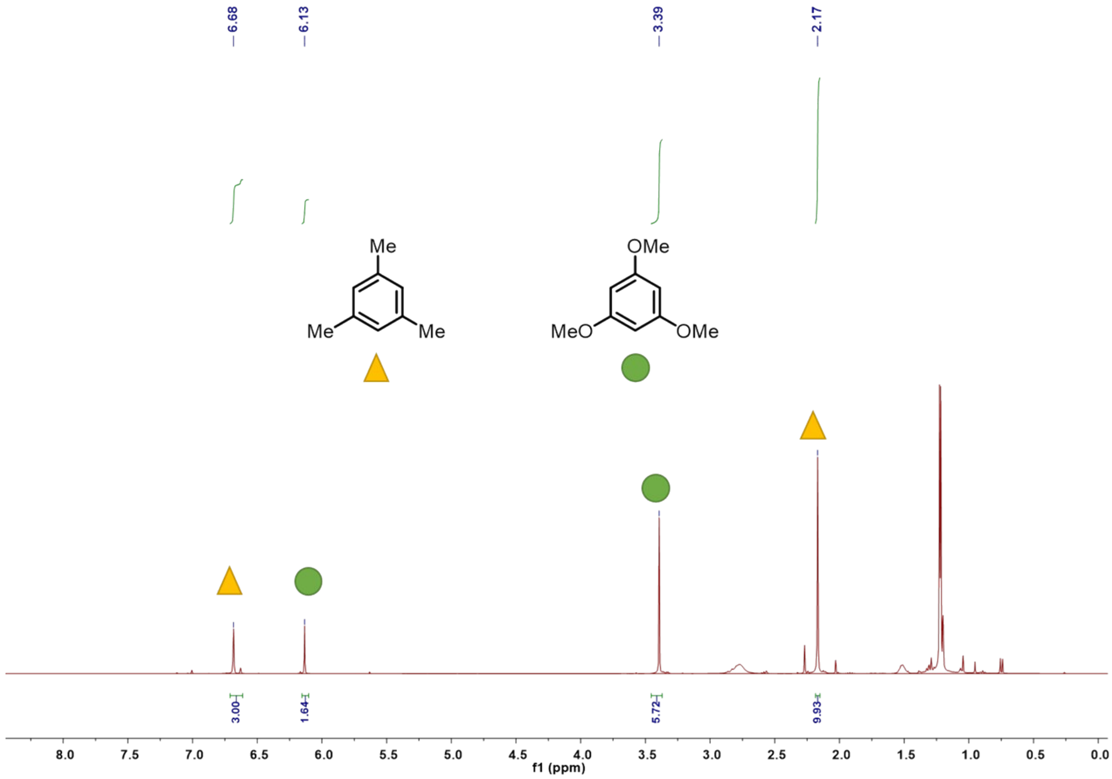

(1) ^{31}P NMR in toluene-d_8

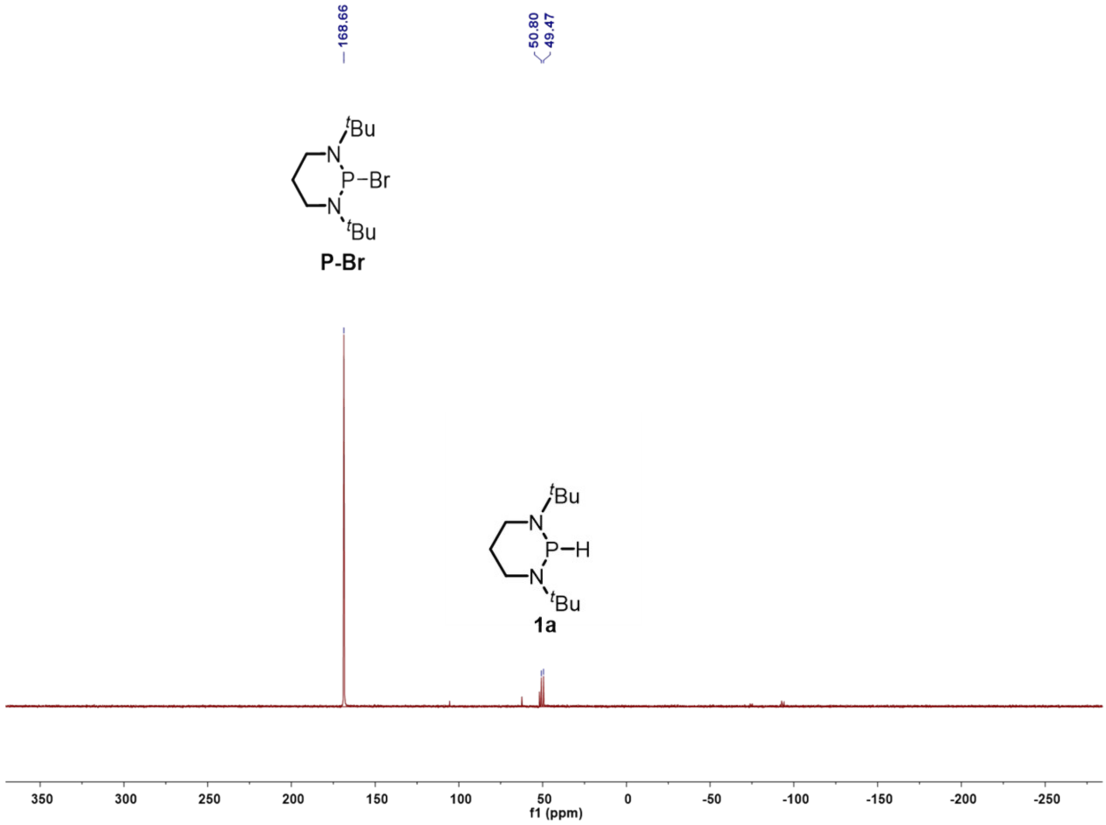

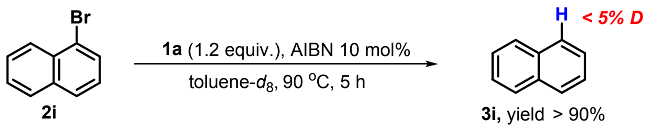

2i (0.1 mmol), AIBN (0.01 mmol), **1a** (0.12 mmol), internal standard (1,3,5-trimethoxybenzene, 0.07 mmol) and toluene-d_8 (0.5 mL) were taken in a NMR tube under argon at 90 °C for 5 hours. The deuterated ratio of the product **3i** was determined by in situ ^{1}H NMR spectrum in toluene-d_8. And the ^{1}H NMR spectra of mixture for the reaction were shown as follow:

(1) ^{1}H NMR in toluene-d_8

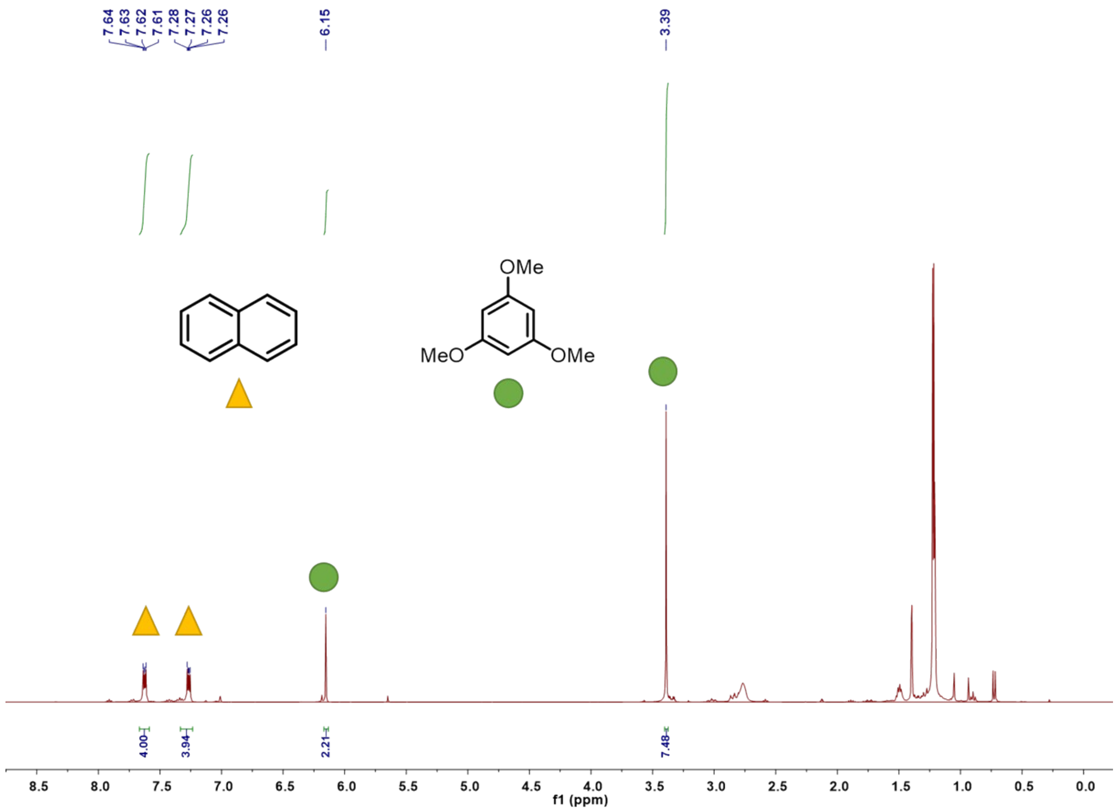

(2) ^{31}P NMR in toluene-d_8

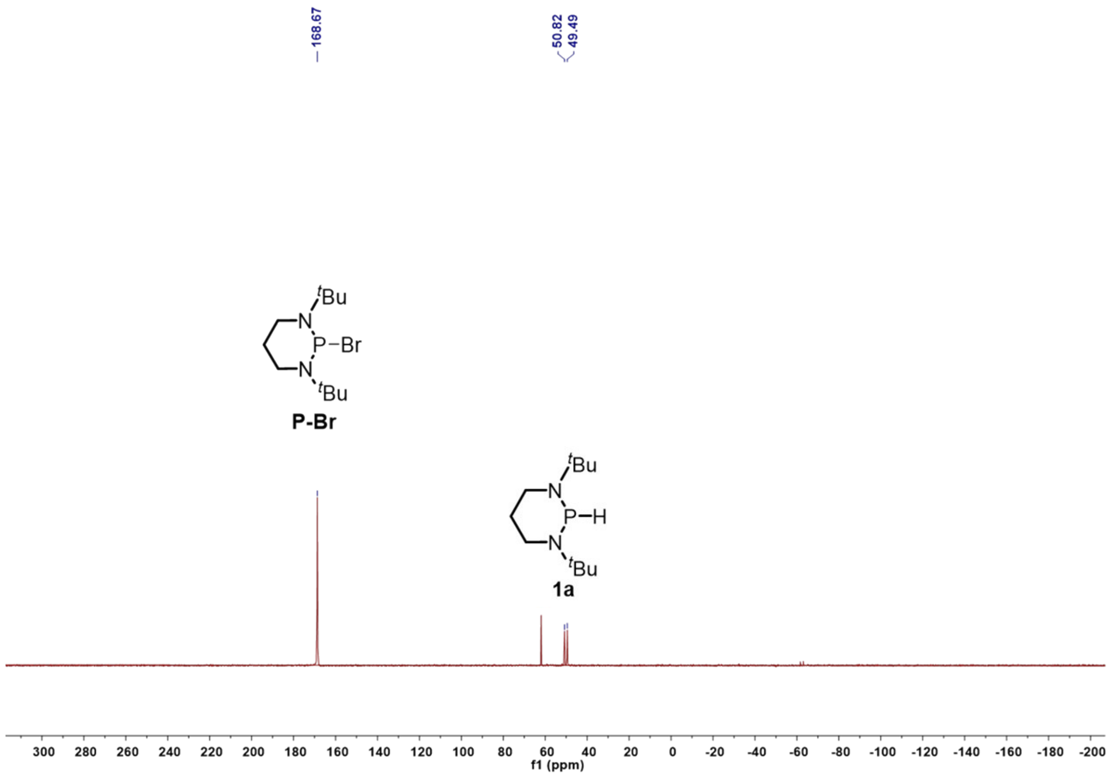

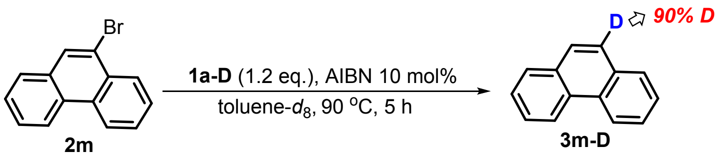

2m (0.1 mmol), AIBN (0.01 mmol), **1a-D** (0.12 mmol) and toluene-d_8 (0.5 mL) were taken in a NMR tube under argon at 90 °C for 5 hours. The deuterated ratio of the product **3m** was determined by in situ ^{1}H NMR spectrum in toluene-d_8. And the NMR spectra of mixture for the reaction were shown as follow:

(1) ^{1}H NMR in toluene-d_8

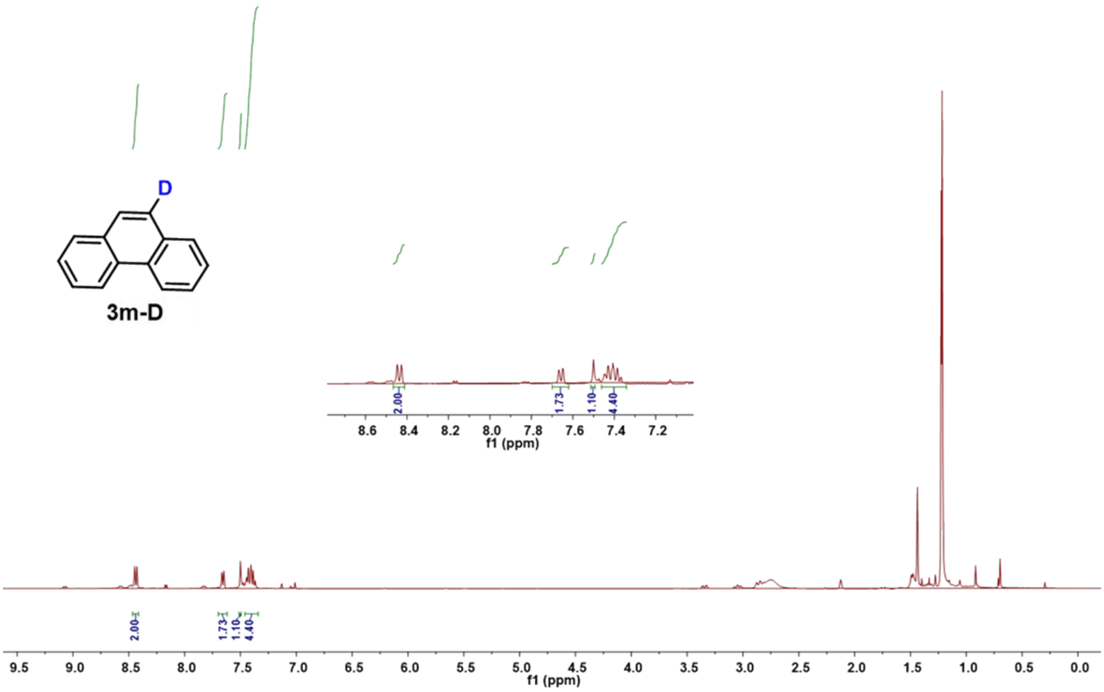

(2) ^{31}P NMR in toluene-d_8

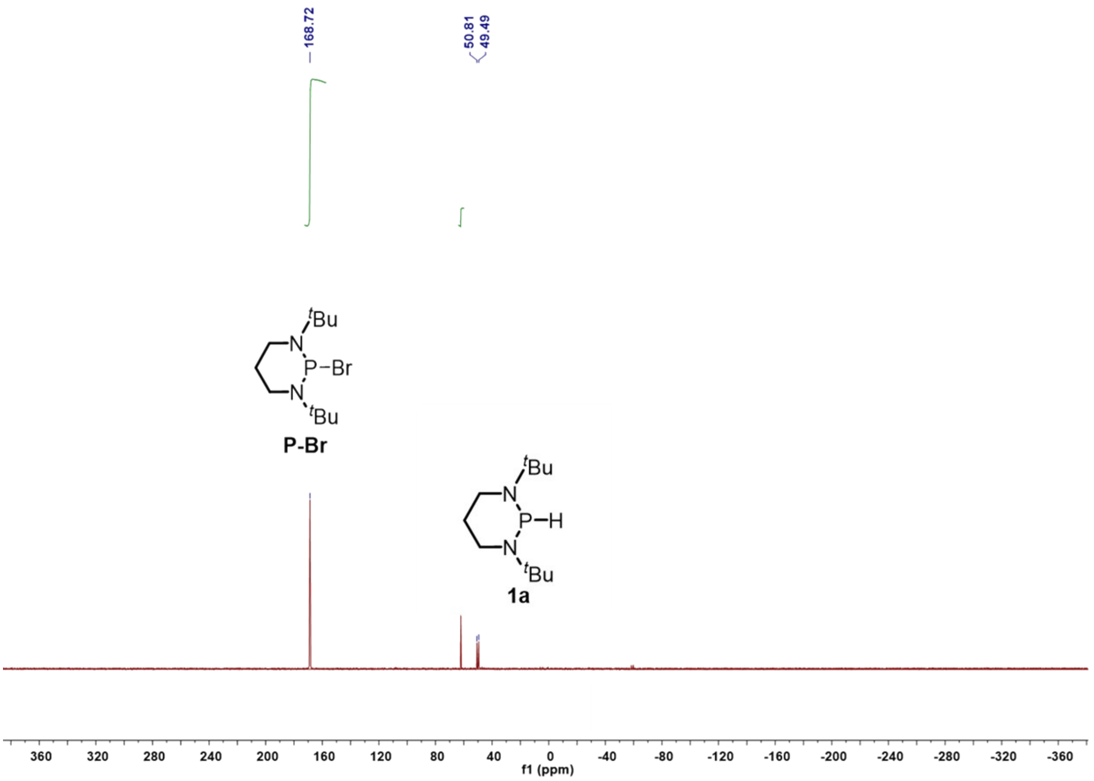

9. DFT Calculations.

Quantum chemistry calculations were conducted by using Gaussian 09[11]. Geometry optimizations and frequency computations were performed using the M06-2X[12] density functional in conjunction with the 6-31+G(d) basis set and an ultrafine integration grid. The SMD[13] model was used to account for the solvation

effects of toluene, the solvent used experimentally. All of the optimized geometries were characterized as minima or transition state structures by frequency calculations. Thermal free energy corrections were obtained at 293.15 K. To obtain more accurate electronic energies, single-point energy calculations were performed at the (SMD)-M06-2X/6-311++G(2df,2p) level with the (SMD)-M06-2X/6-31+G(d) optimized structure.

The differences of bond dissociation free energies of P-Br bonds of **1a-Br** and **1b-Br** were calculated on the basic of reaction Gibbs free energy changes of Eq. S1-2 through DFT calculations. The P-Br bond of **1a-Br** is only 1.3 kcal/mol larger than that of **1b-Br**, however, the reaction did not work at all when **1b** was as the reductant. Therefore, the mechanism that the phosphinyl radicals abstract the bromine atom od bromobenzene to perform the hydrodebromination was excluded.

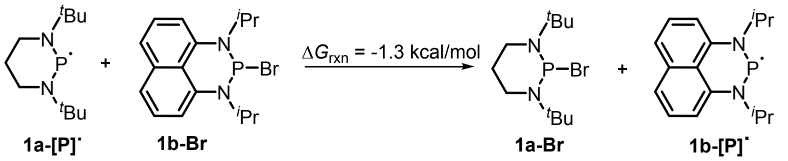

(S1)

$$\Delta G_{rxn} = \mathrm{BDFE}_{\mathbf{1b\text{-}Br}}(\text{P-Br}) - \mathrm{BDFE}_{\mathbf{1a\text{-}Br}}(\text{P-Br}) = -1.3 \text{ kcal/mol} \qquad \text{(S2)}$$

During the course of our mechanism studies, we had deliberately tried to capture the radical species under reaction conditions, and also through the reaction of **1a** with equivalent AIBN at 90 °C. However, no EPR signal was detected in either case, but instead, the dimeric bisphosphine **[1a-P]$_2$** was obtained. These results indicate that the radical **1a-[P]•** is so reactive that it would rather react with bromobenzene or couple with each other once generated. When the dimer **[1a-P]$_2$** was directly used as the reductant, the hydrodehalogenation did not occur (Eq. 1 of Scheme 5). This is presumably because **[1a-P]$_2$** cannot easily dissociate to render the corresponding phosphinyl radical **1a-[P]•** at the reaction temperature (90 °C).

Furthermore, DFT calculations were conducted to evaluate the stability of the radical and dimers. The dissociation free energies of the P-P bonds in **[1a-P]$_2$** and **[B-P]$_2$** ($\Delta G(1)_{Diss}$ and $\Delta G(2)_{Diss}$, see below) were estimated at the M06-2x/6-31+G(d,p)//6-311++G(2df,2p) level of theory with the SMD model for the solvation of toluene. As seen, the P-P bond of **[1a-P]$_2$** is much stronger (by 13.4 kcal/mol) than that of **[B-P]$_2$**. Wright's observation showed that no solution EPR signal could be detected for **[B-P]$_2$** in toluene at room temperature.[14] As the temperature was increased to 80 °C (353 K), a weak EPR signal of **B-[P]•** was captured. Considering the much stronger P-P bond of **[1a-P]$_2$**, it should be stable enough to tolerate our reaction temperature (90 °C). This may explain the absence of **1a-[P]•** EPR signal as well as the failure of the reaction of bromobenzene with **[1a-P]$_2$**.

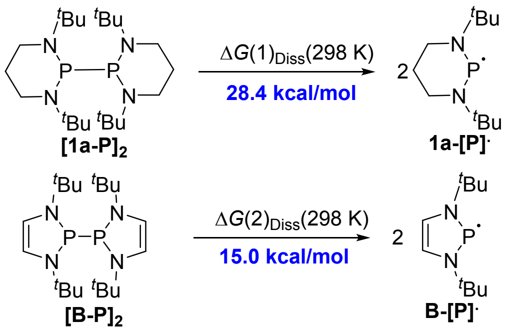

10. Representative NMR Spectra.

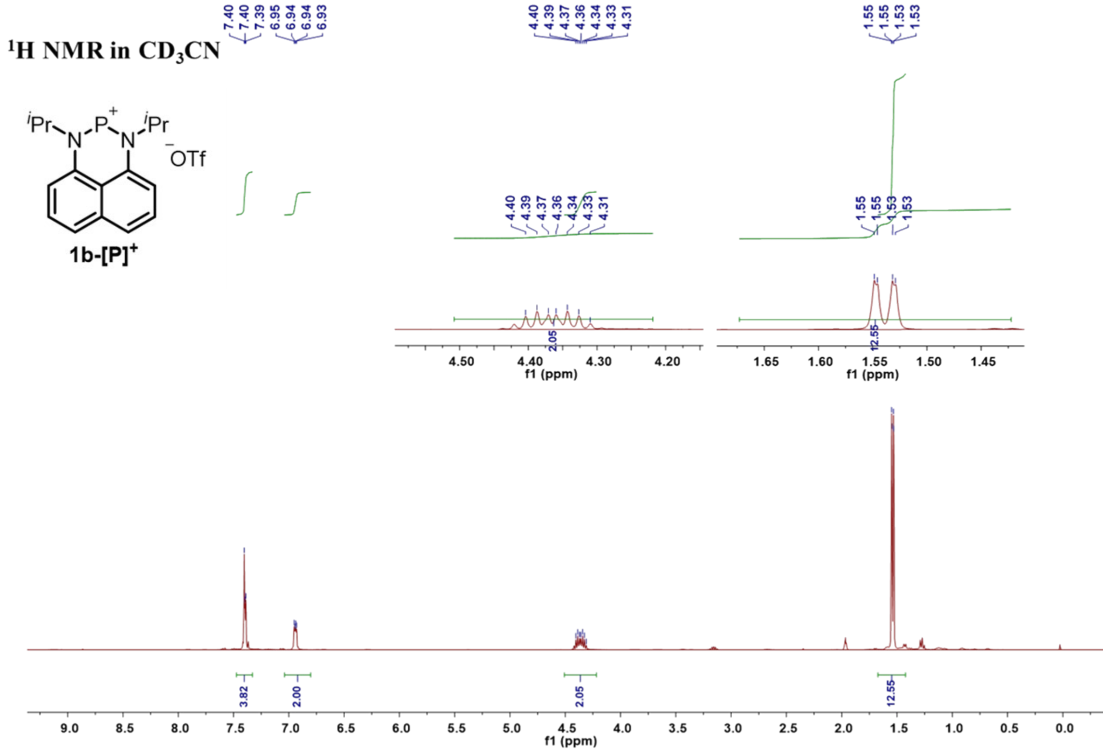

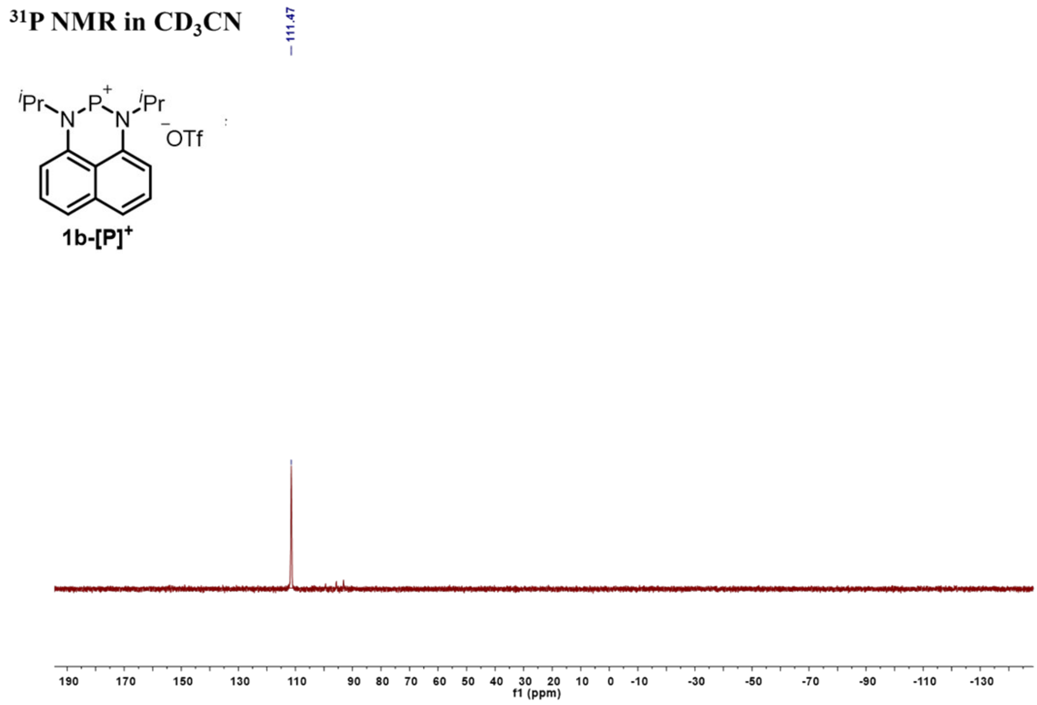

^{13}C NMR in CD_3CN

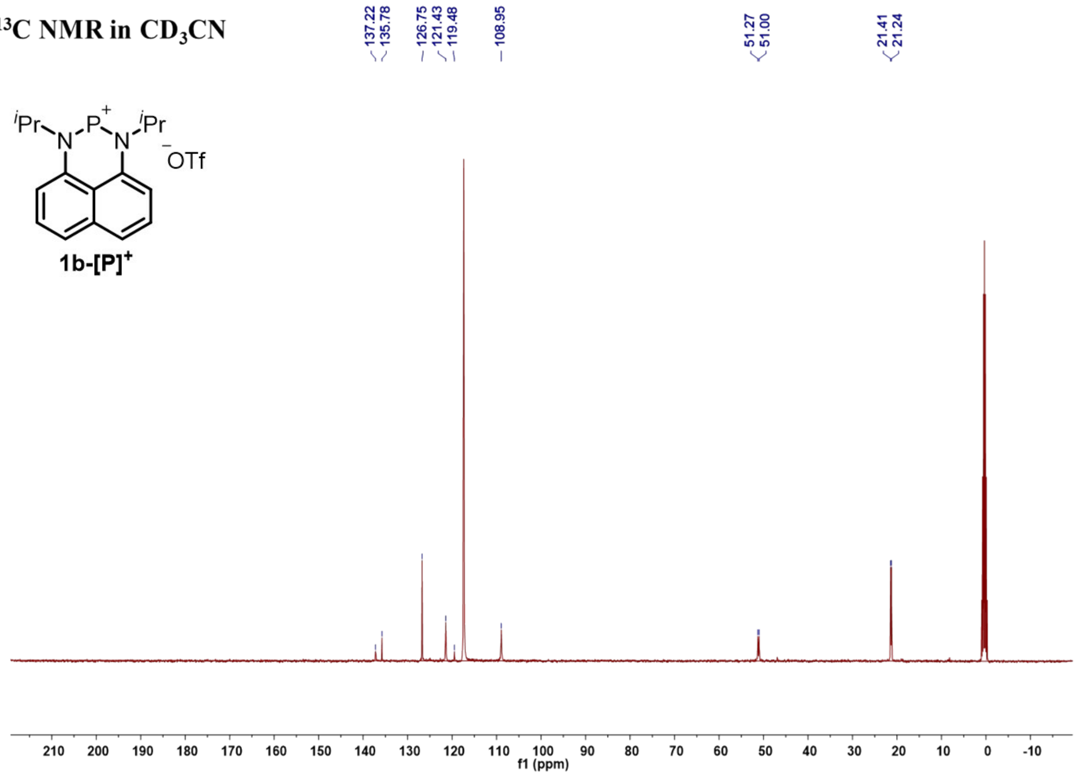

^{1}H NMR in toluene-d_8

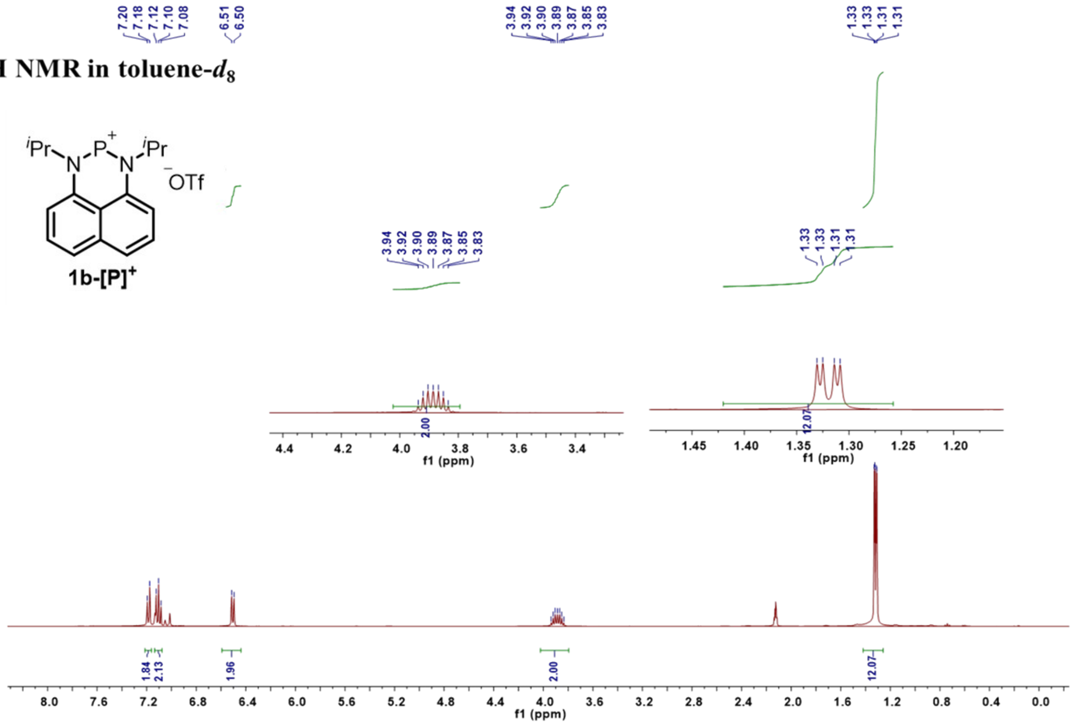

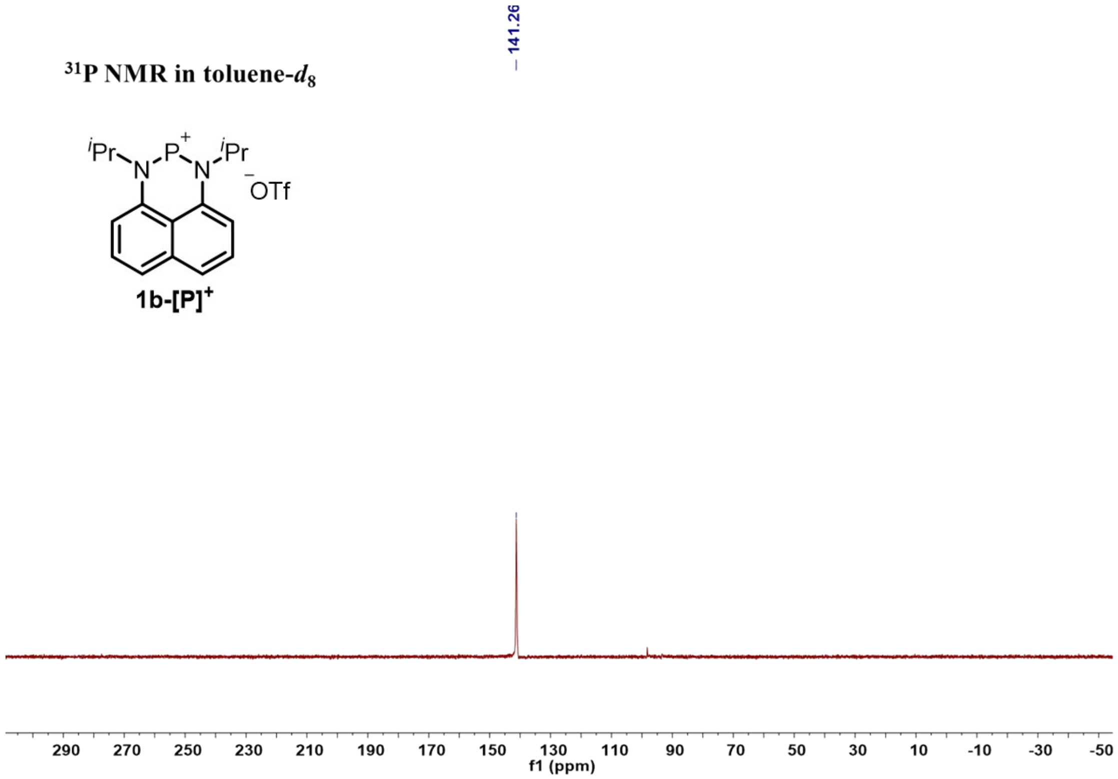

31P NMR in toluene-d8
iPr
N
P
+
N
iPr
−OTf
1b-[P]+
141.26
290
270
250
230
210
190
170
150
130
110
90
70
50
30
10
-10
-30
-50
f1 (ppm)

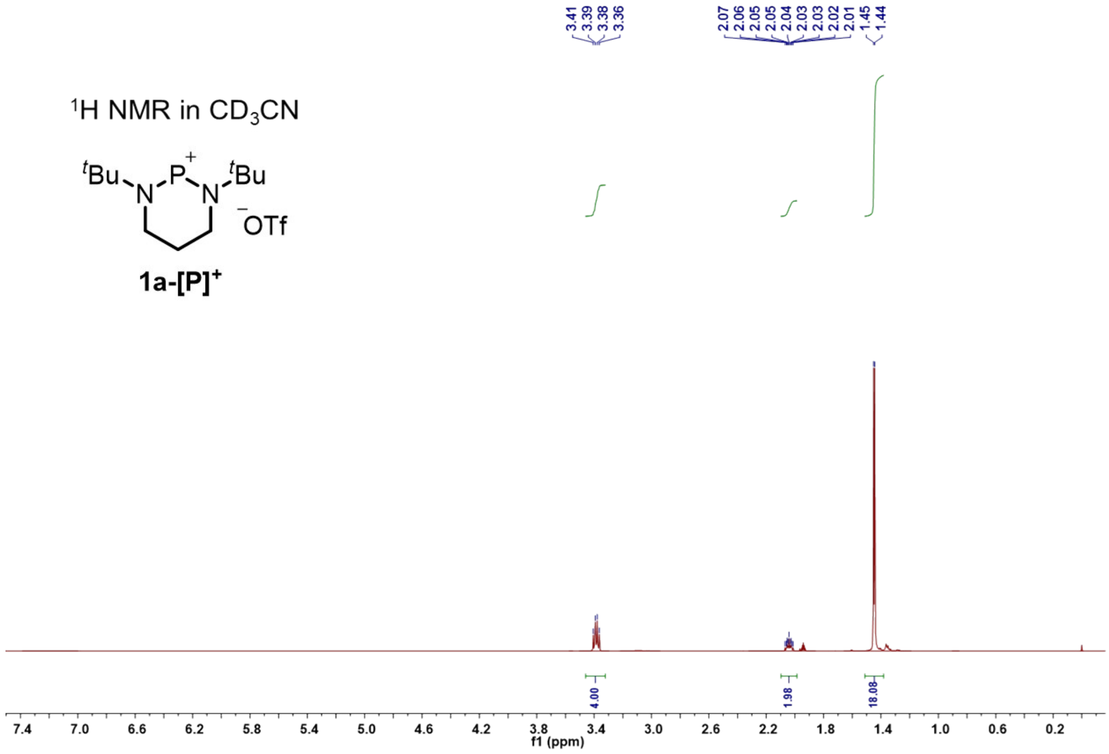

1H NMR in CD3CN
tBu
N
P
+
N
tBu
−OTf
1a-[P]+
3.41
3.39
3.38
3.36
2.07
2.06
2.05
2.04
2.03
2.03
2.02
2.01
1.45
1.44
4.00
1.98
18.08
7.4
7.0
6.6
6.2
5.8
5.4
5.0
4.6
4.2
3.8
3.4
3.0
2.6
2.2
1.8
1.4
1.0
0.6
0.2
f1 (ppm)

^{13}C NMR in CD_3CN

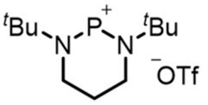

1a-[P]$^+$

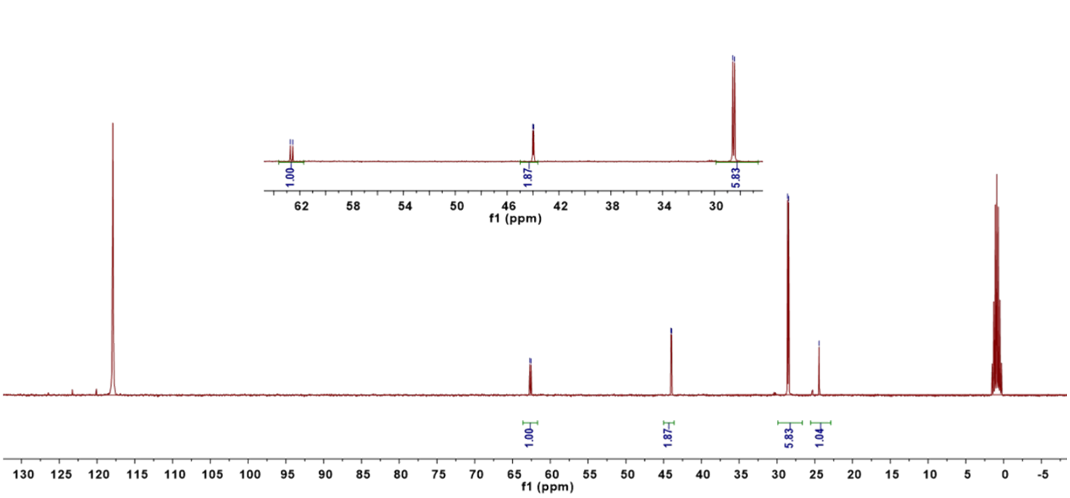

^{31}P NMR in CD_3CN

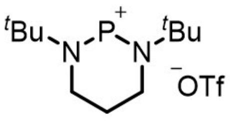

1a-[P]$^+$

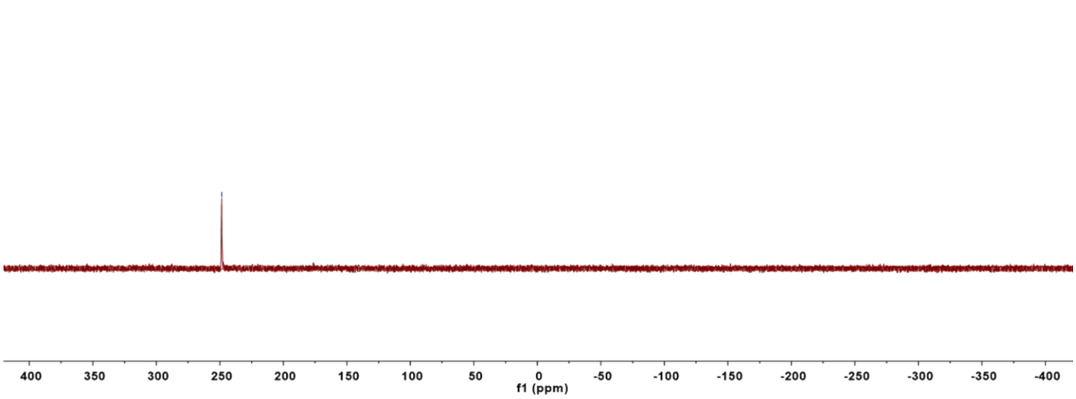

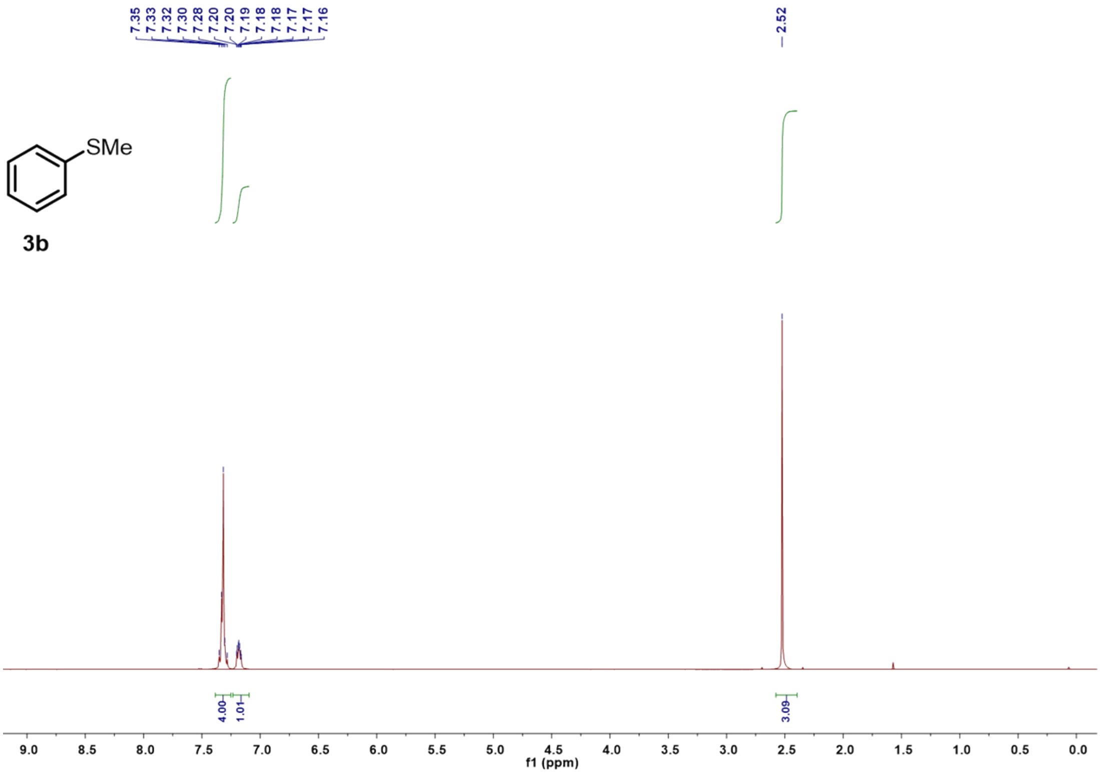
7.35
7.33
7.32
7.30
7.28
7.20
7.20
7.19
7.18
7.18
7.17
7.17
7.16
2.52
SMe
3b
4.00
1.01
3.09
9.0 8.5 8.0 7.5 7.0 6.5 6.0 5.5 5.0 4.5 4.0 3.5 3.0 2.5 2.0 1.5 1.0 0.5 0.0
f1 (ppm)

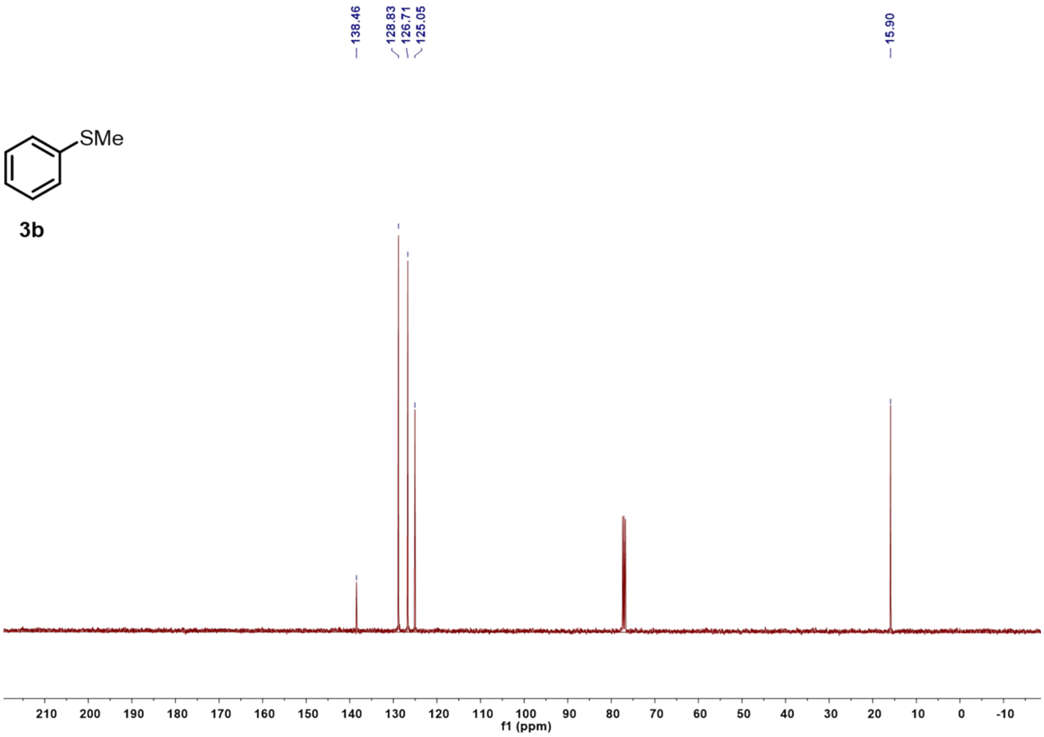
138.46
128.83
126.71
125.05
15.90
SMe
3b
210 200 190 180 170 160 150 140 130 120 110 100 90 80 70 60 50 40 30 20 10 0 -10
f1 (ppm)

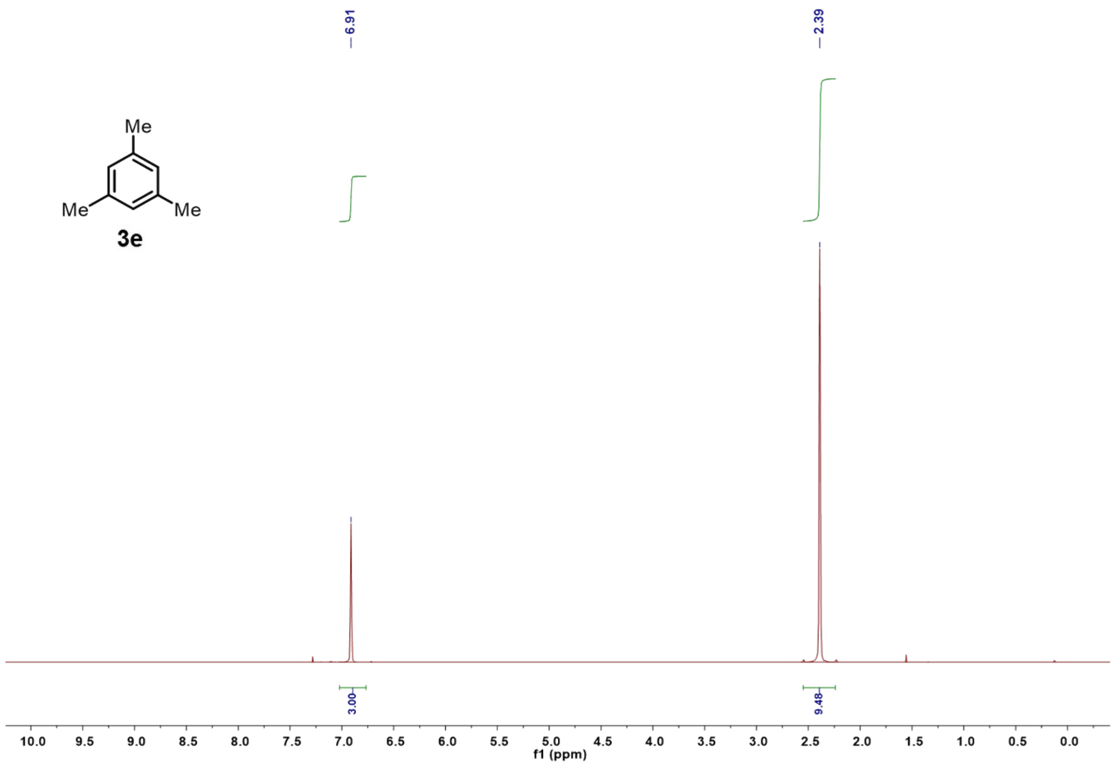

6.91
2.39
Me
Me
Me
3e
3.00
9.48
10.0
9.5
9.0
8.5
8.0
7.5
7.0
6.5
6.0
5.5
5.0
4.5
4.0
3.5
3.0
2.5
2.0
1.5
1.0
0.5
0.0
f1 (ppm)

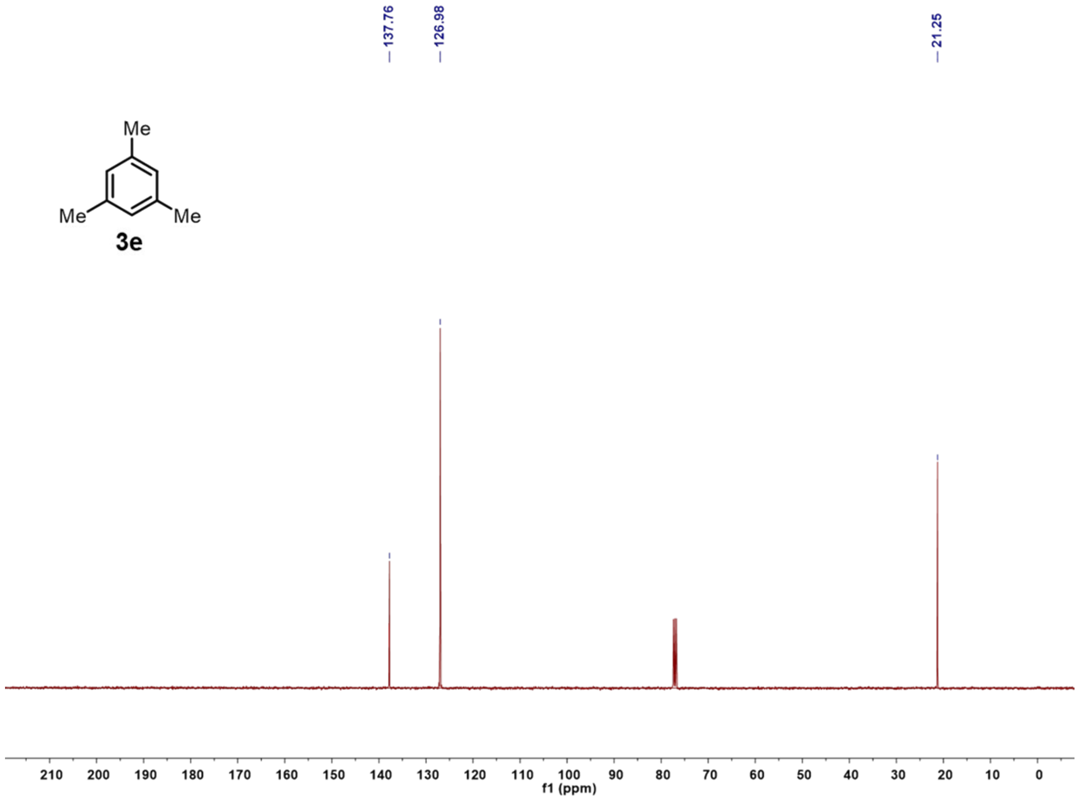

137.76
126.98
21.25
Me
Me
Me
3e
210
200
190
180
170
160
150
140
130
120
110
100
90
80
70
60
50
40
30
20
10
0
f1 (ppm)

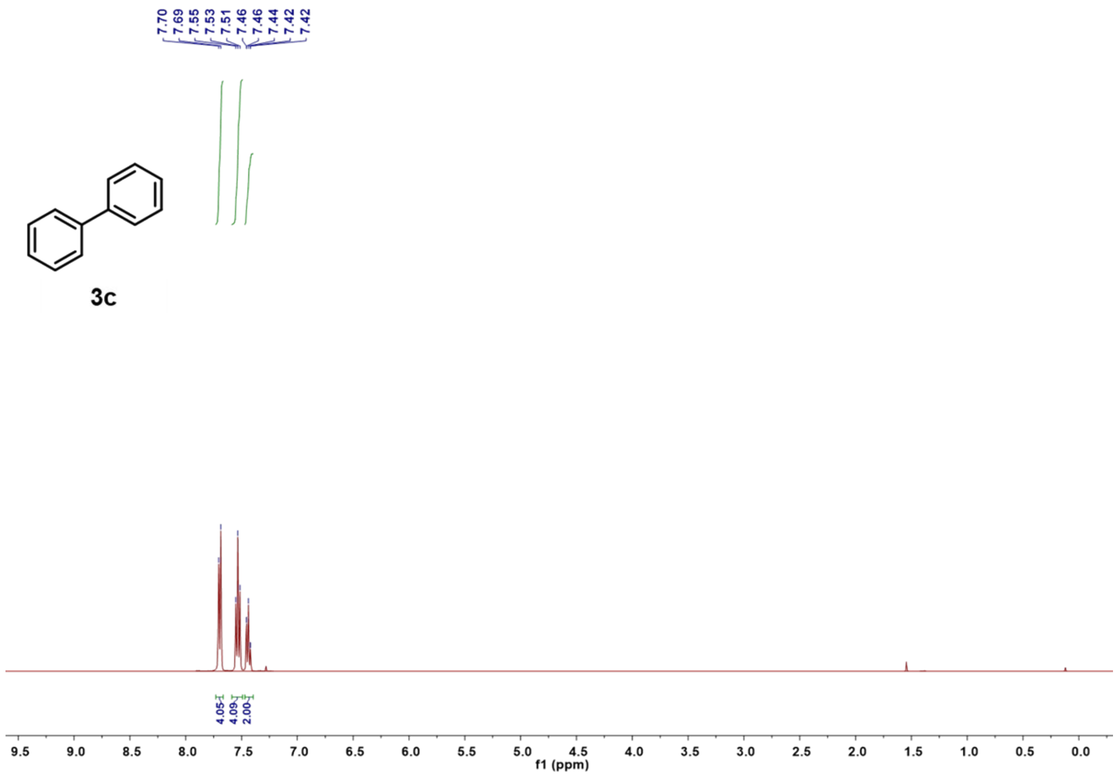
7.70
7.69
7.55
7.53
7.51
7.46
7.46
7.44
7.42
7.42
3c
4.05
4.09
2.00
9.5 9.0 8.5 8.0 7.5 7.0 6.5 6.0 5.5 5.0 4.5 4.0 3.5 3.0 2.5 2.0 1.5 1.0 0.5 0.0
f1 (ppm)

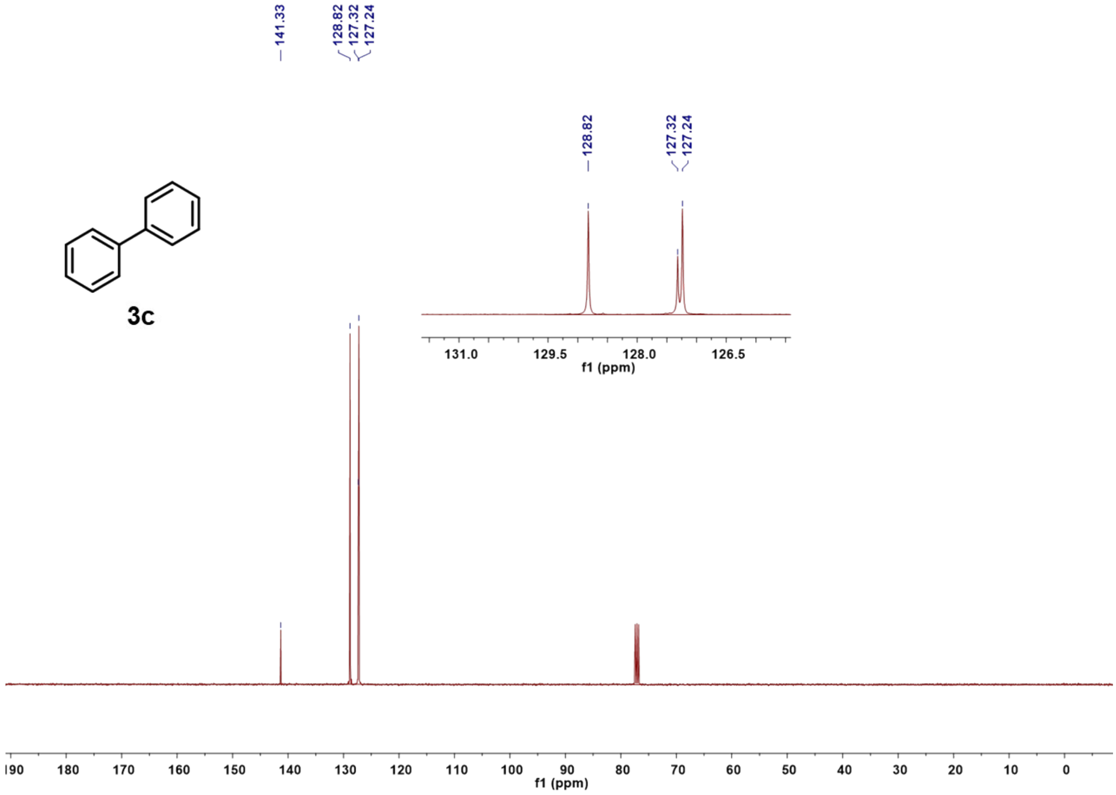
141.33
128.82
127.32
127.24
128.82
127.32
127.24
3c
131.0 129.5 128.0 126.5
f1 (ppm)
190 180 170 160 150 140 130 120 110 100 90 80 70 60 50 40 30 20 10 0
f1 (ppm)

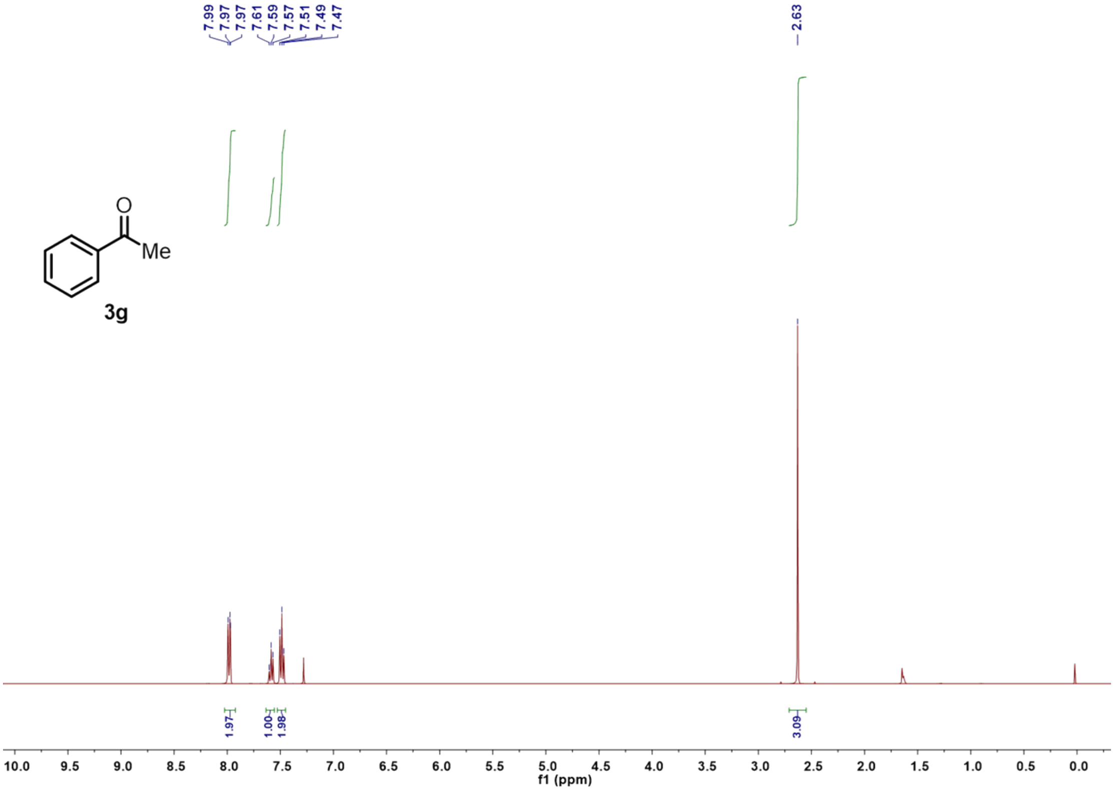

7.99
7.97
7.97
7.61
7.59
7.57
7.51
7.49
7.47
2.63
O
Me
1.97
1.00
1.98
3.09
10.0 9.5 9.0 8.5 8.0 7.5 7.0 6.5 6.0 5.5 5.0 4.5 4.0 3.5 3.0 2.5 2.0 1.5 1.0 0.5 0.0
f1 (ppm)

3g

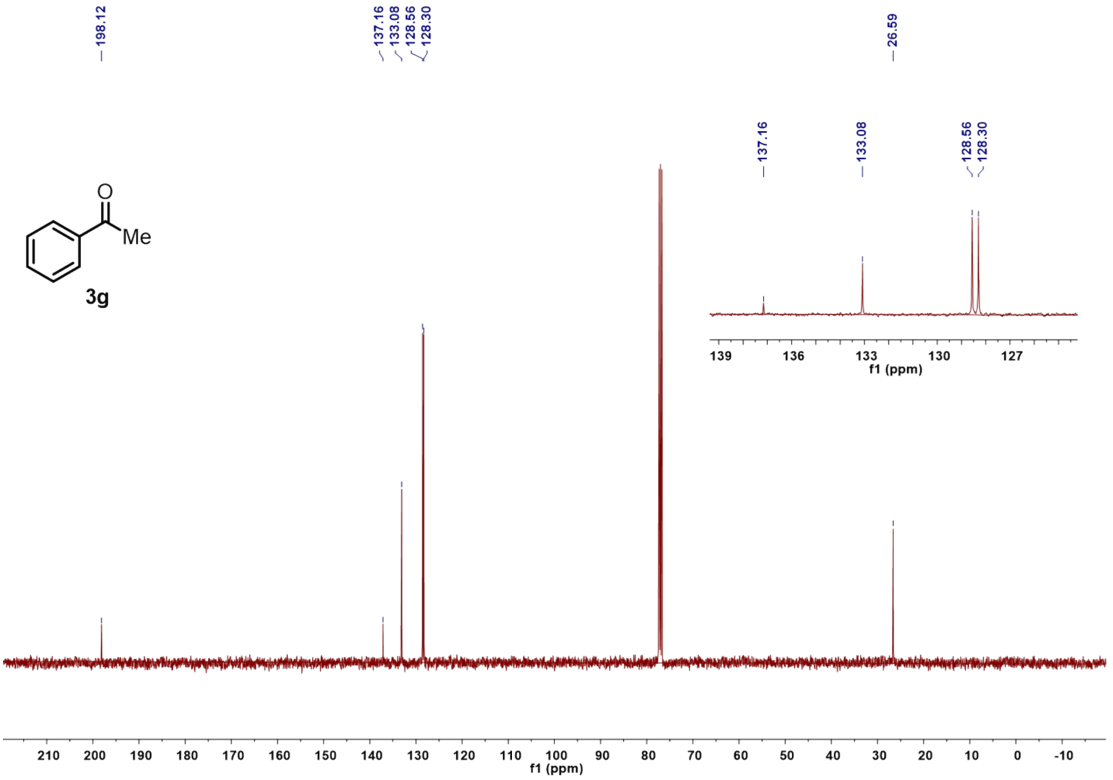

198.12
137.16
133.08
128.56
128.30
26.59
O
Me
139 136 133 130 127
f1 (ppm)
210 200 190 180 170 160 150 140 130 120 110 100 90 80 70 60 50 40 30 20 10 0 -10
f1 (ppm)

3g

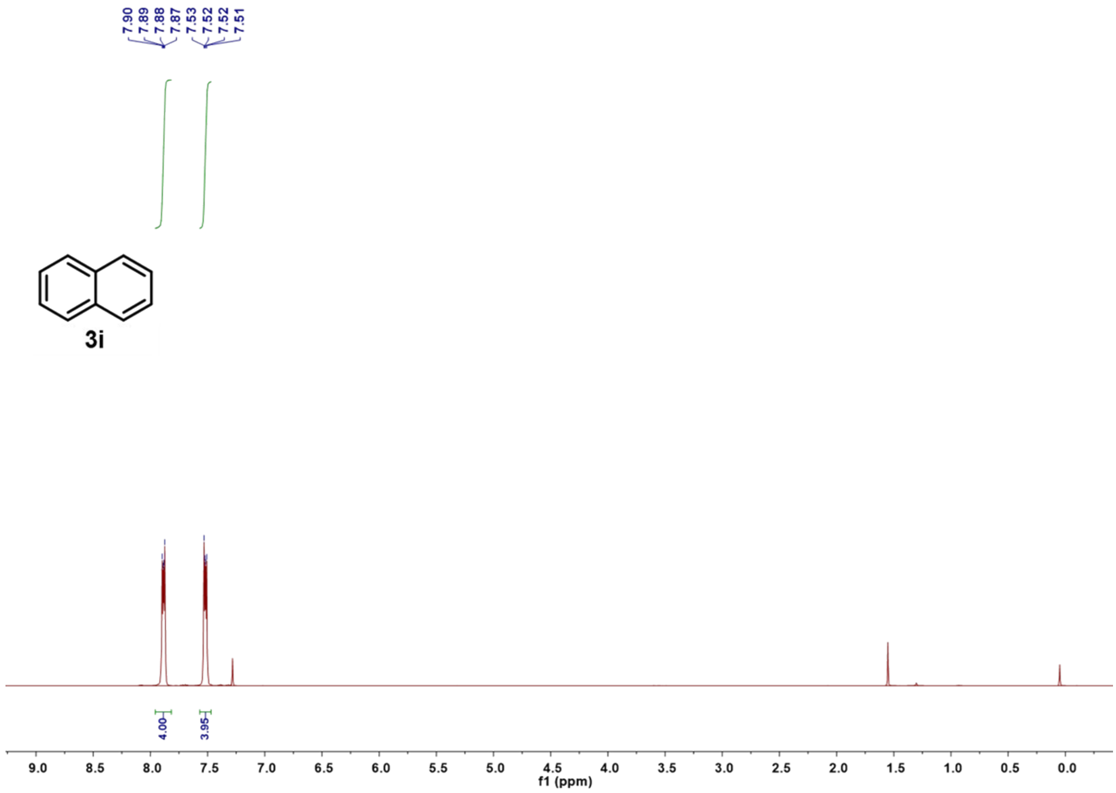
7.90
7.89
7.88
7.87
7.53
7.52
7.52
7.51
3i
4.00
3.95
9.0
8.5
8.0
7.5
7.0
6.5
6.0
5.5
5.0
4.5
4.0
3.5
3.0
2.5
2.0
1.5
1.0
0.5
0.0
f1 (ppm)

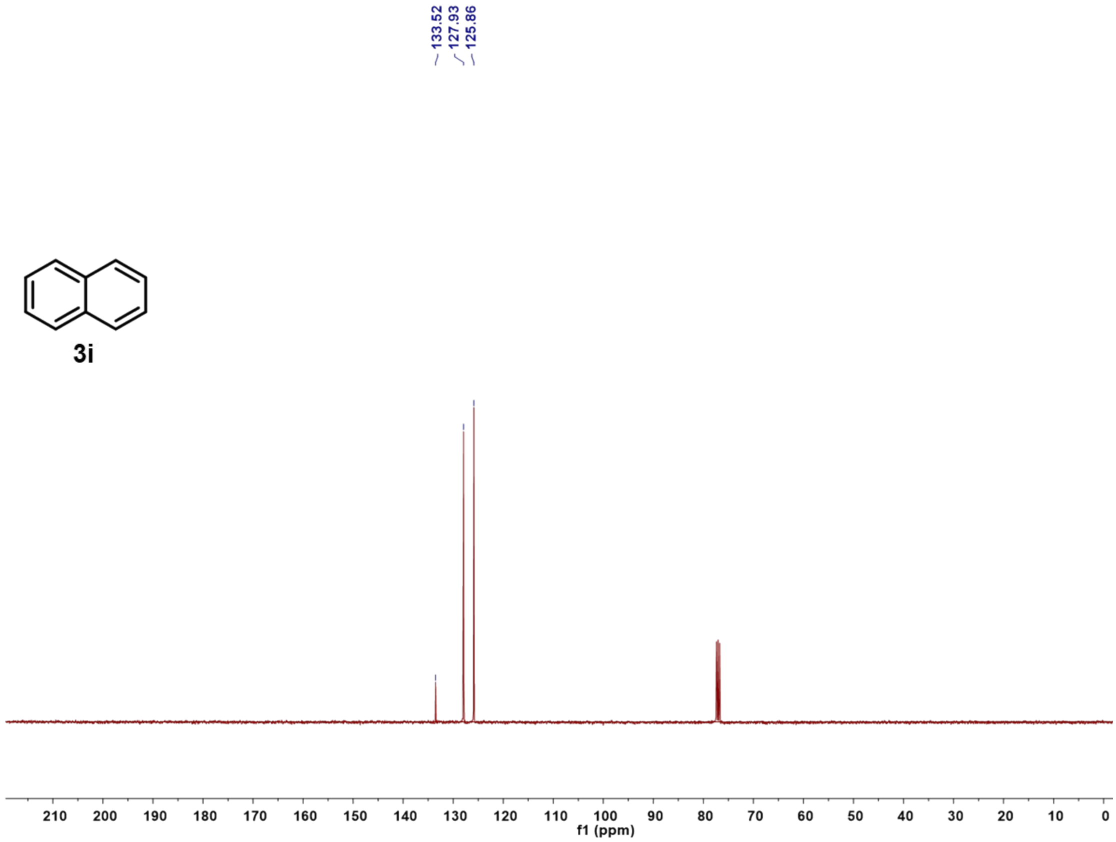
133.52
127.93
125.86
3i
210
200
190
180
170
160
150
140
130
120
110
100
90
80
70
60
50
40
30
20
10
0
f1 (ppm)

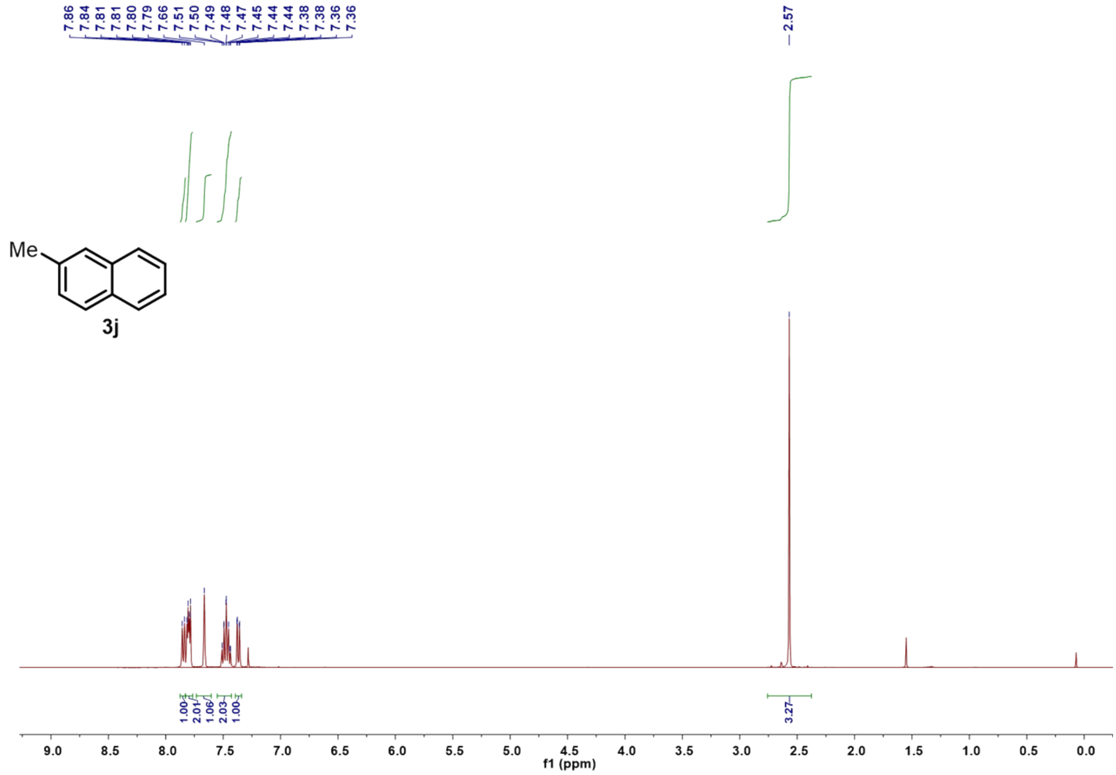
7.86 7.84 7.81 7.81 7.80 7.79 7.66 7.51 7.50 7.49 7.48 7.47 7.45 7.44 7.44 7.38 7.38 7.36 7.36
2.57
Me
3j
1.00 2.01 1.06 2.03 1.00
3.27
f1 (ppm)

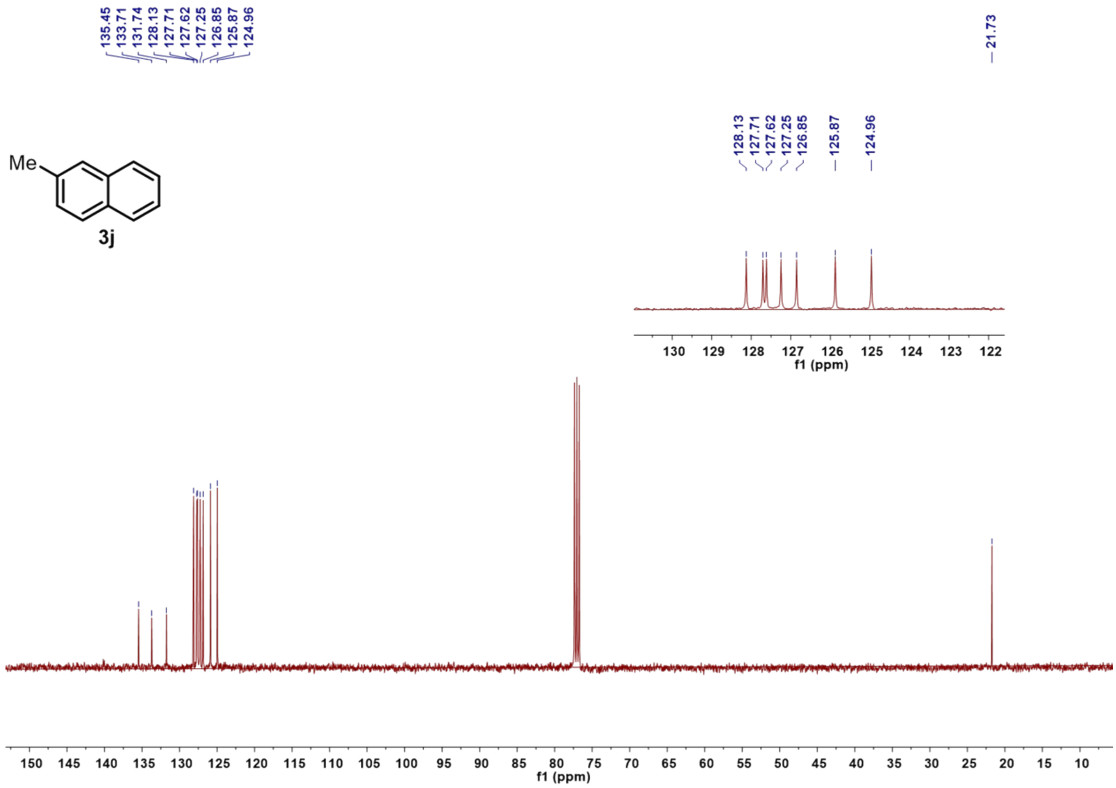
135.45 133.71 131.74 128.13 127.71 127.62 127.25 126.85 125.87 124.96
21.73
Me
3j
128.13 127.71 127.62 127.25 126.85 125.87 124.96
f1 (ppm)
f1 (ppm)

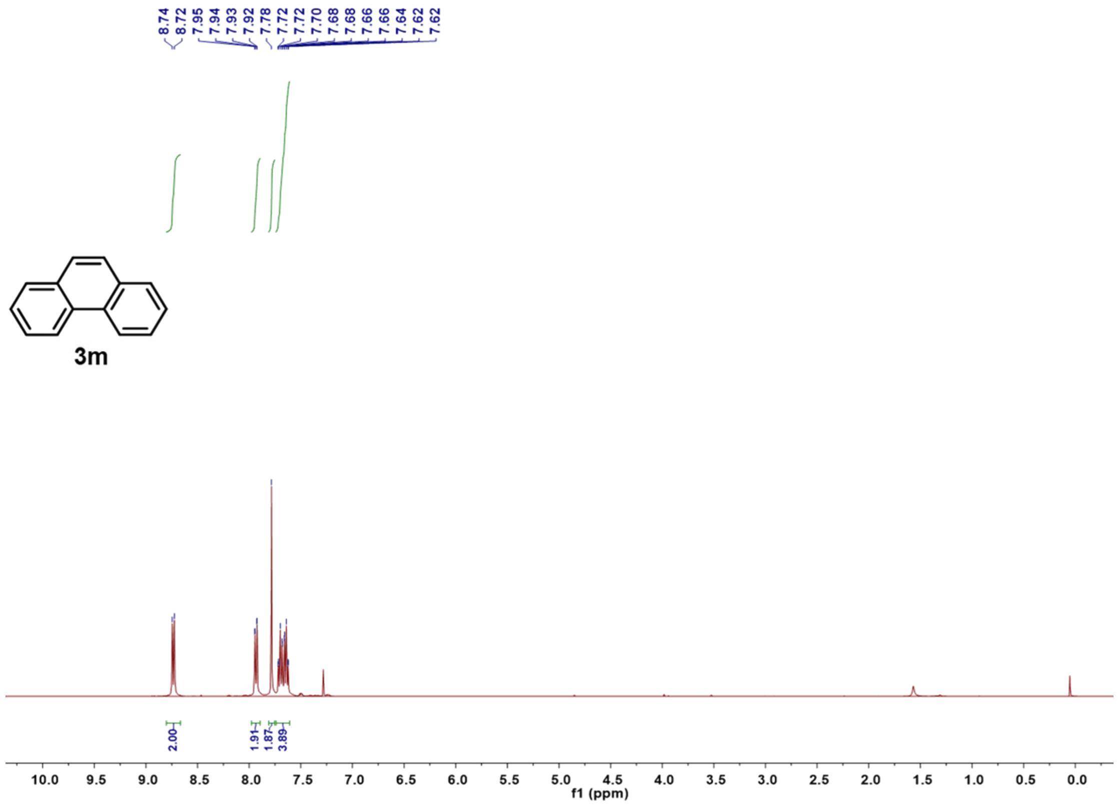

8.74
8.72
7.95
7.94
7.93
7.92
7.78
7.72
7.72
7.70
7.68
7.68
7.66
7.66
7.64
7.62
7.62
3m
2.00
1.91
1.87
3.89
10.0 9.5 9.0 8.5 8.0 7.5 7.0 6.5 6.0 5.5 5.0 4.5 4.0 3.5 3.0 2.5 2.0 1.5 1.0 0.5 0.0
f1 (ppm)

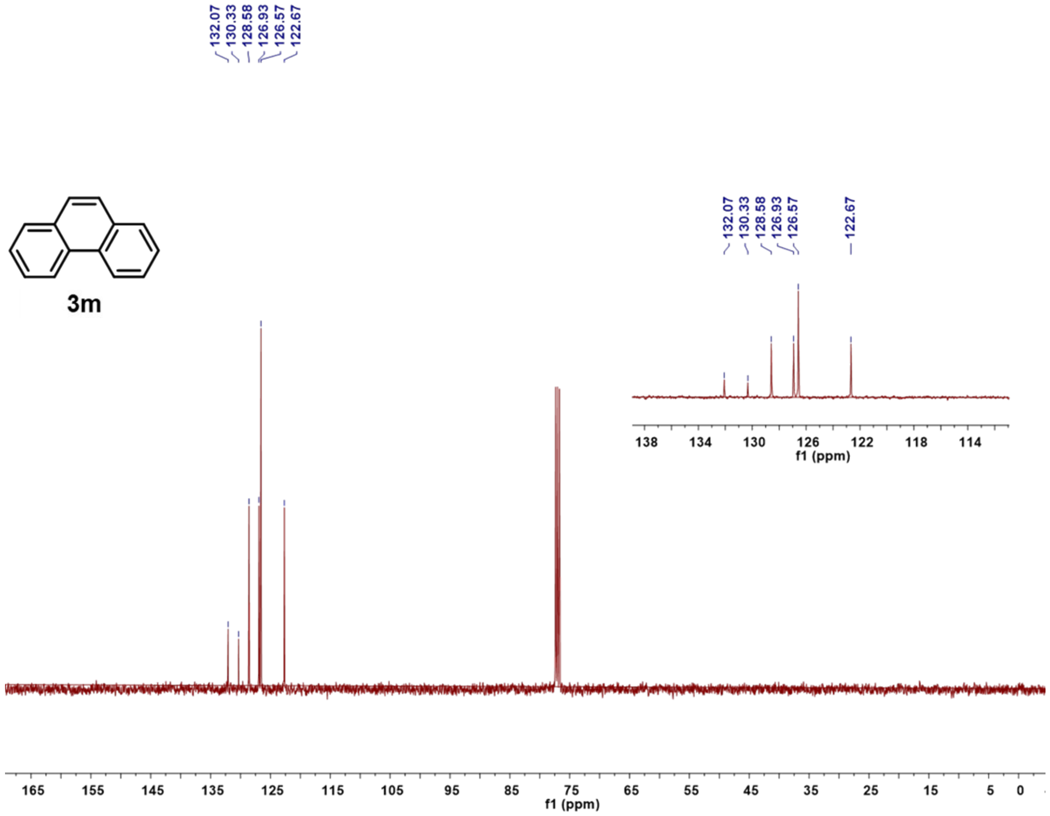

132.07
130.33
128.58
126.93
126.57
122.67
3m
138 134 130 126 122 118 114
f1 (ppm)
165 155 145 135 125 115 105 95 85 75 65 55 45 35 25 15 5 0
f1 (ppm)

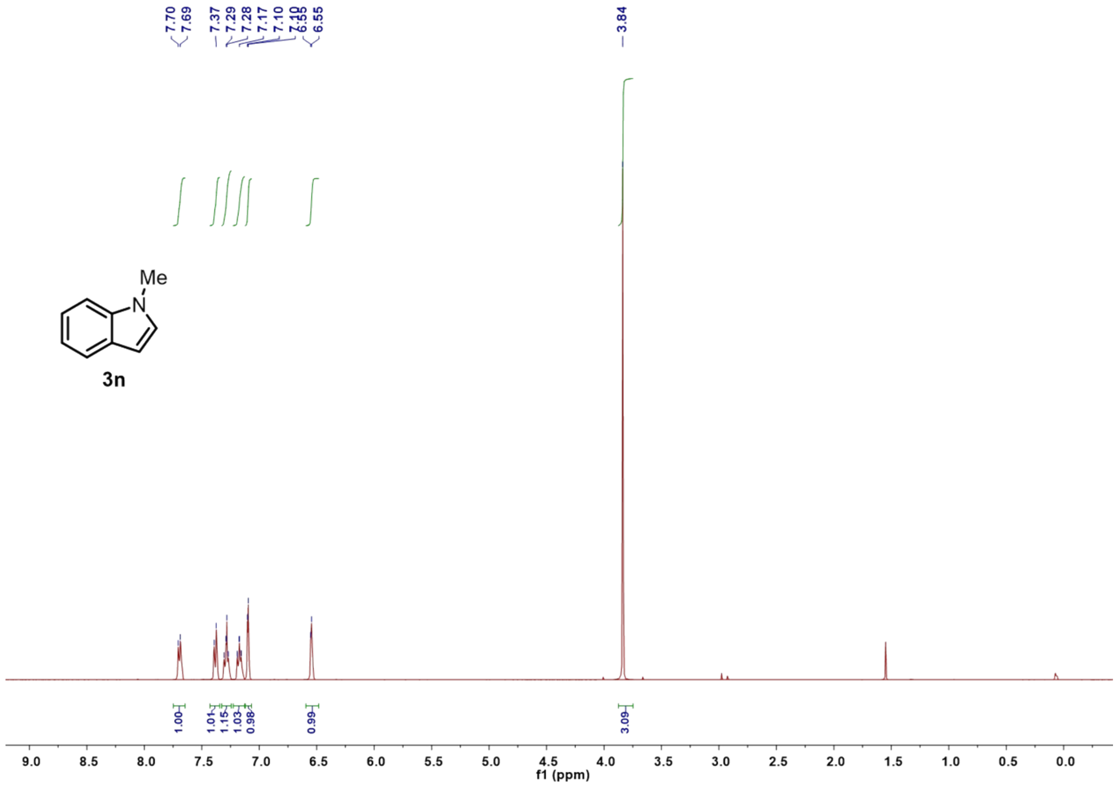
7.70
7.69
7.37
7.29
7.28
7.17
7.10
7.10
6.55
6.55
3.84
Me
N
3n
1.00
1.01
1.15
1.03
0.98
0.99
3.09
9.0 8.5 8.0 7.5 7.0 6.5 6.0 5.5 5.0 4.5 4.0 3.5 3.0 2.5 2.0 1.5 1.0 0.5 0.0
f1 (ppm)

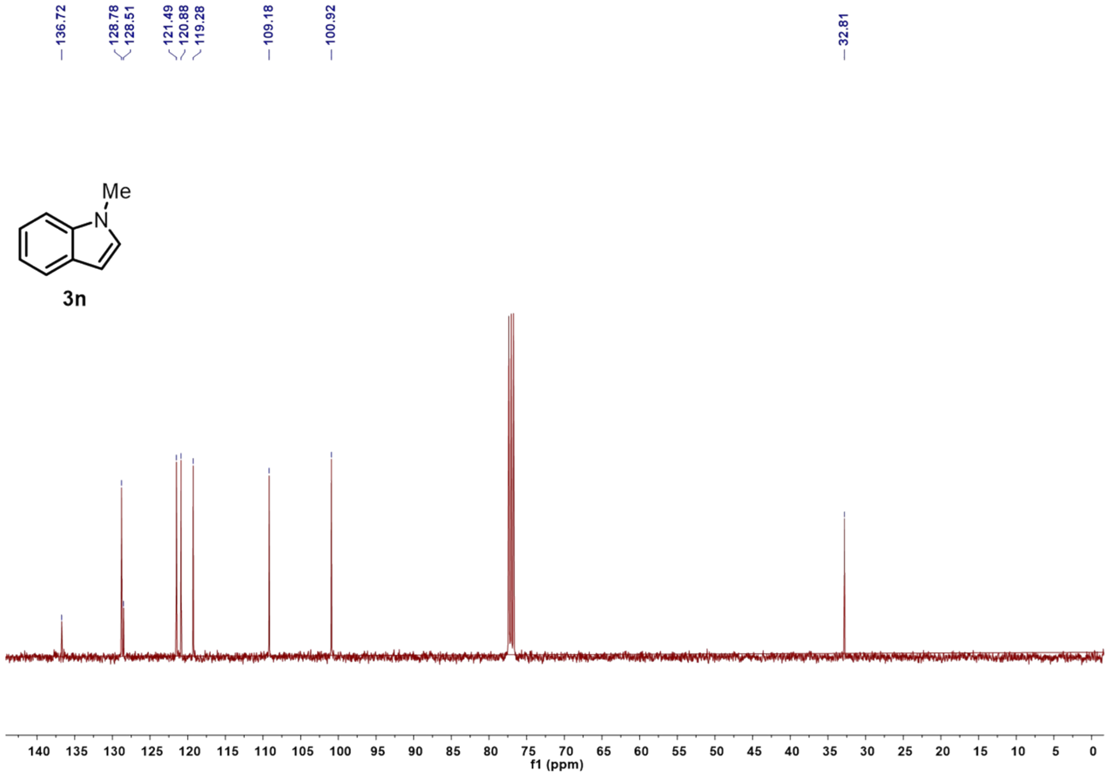
136.72
128.78
128.51
121.49
120.88
119.28
109.18
100.92
32.81
Me
N
3n
140 135 130 125 120 115 110 105 100 95 90 85 80 75 70 65 60 55 50 45 40 35 30 25 20 15 10 5 0
f1 (ppm)

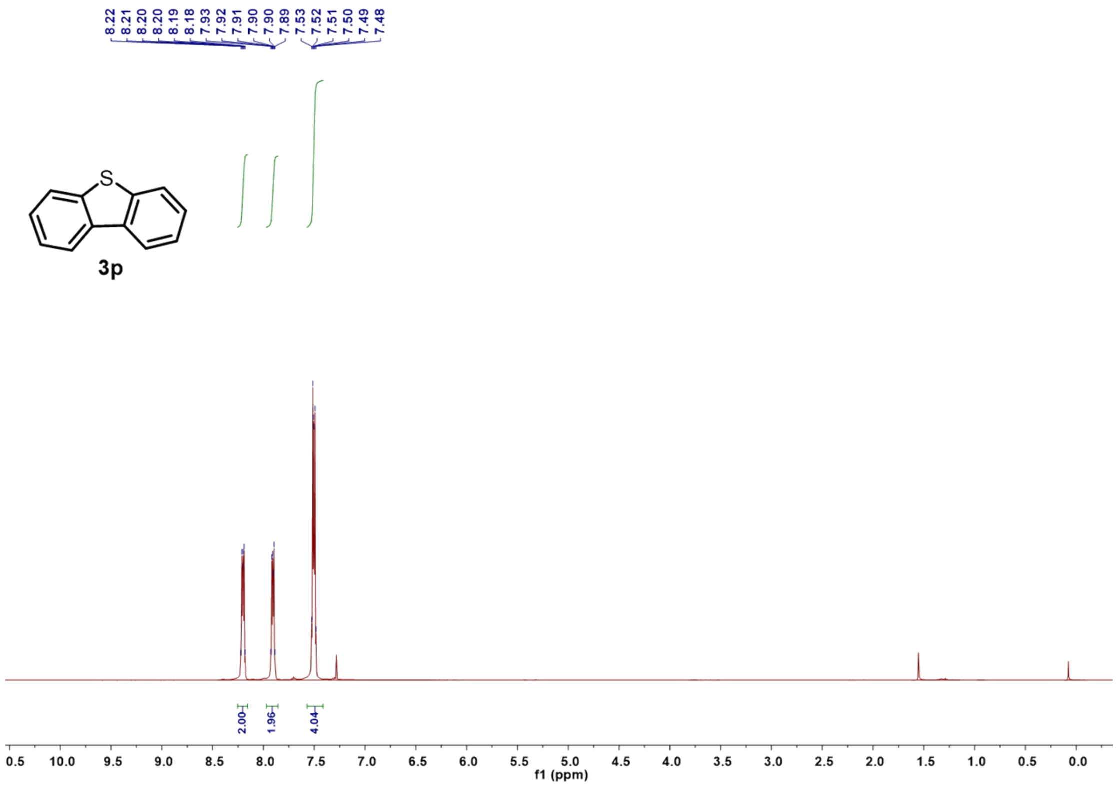
8.22 8.21 8.20 8.20 8.19 8.18 7.93 7.92 7.91 7.90 7.90 7.89 7.53 7.52 7.51 7.50 7.49 7.48
S
3p
2.00
1.96
4.04
0.5 10.0 9.5 9.0 8.5 8.0 7.5 7.0 6.5 6.0 5.5 5.0 4.5 4.0 3.5 3.0 2.5 2.0 1.5 1.0 0.5 0.0
f1 (ppm)

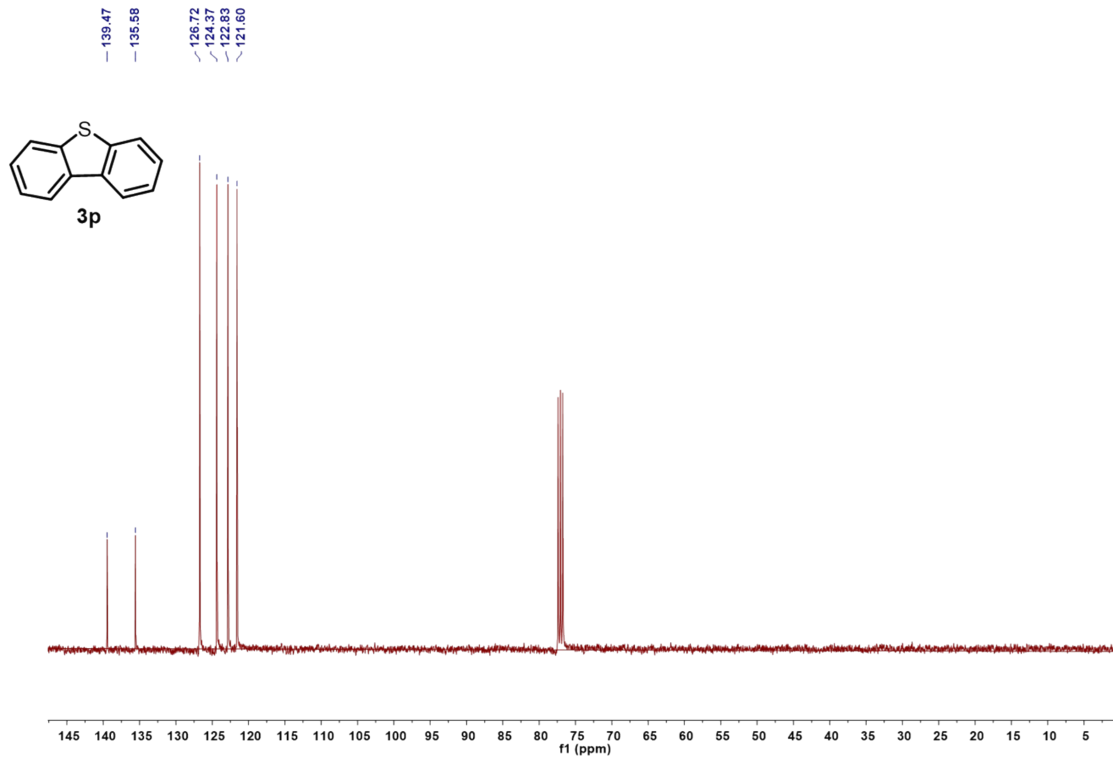
139.47
135.58
126.72 124.37 122.83 121.60
S
3p
145 140 135 130 125 120 115 110 105 100 95 90 85 80 75 70 65 60 55 50 45 40 35 30 25 20 15 10 5
f1 (ppm)

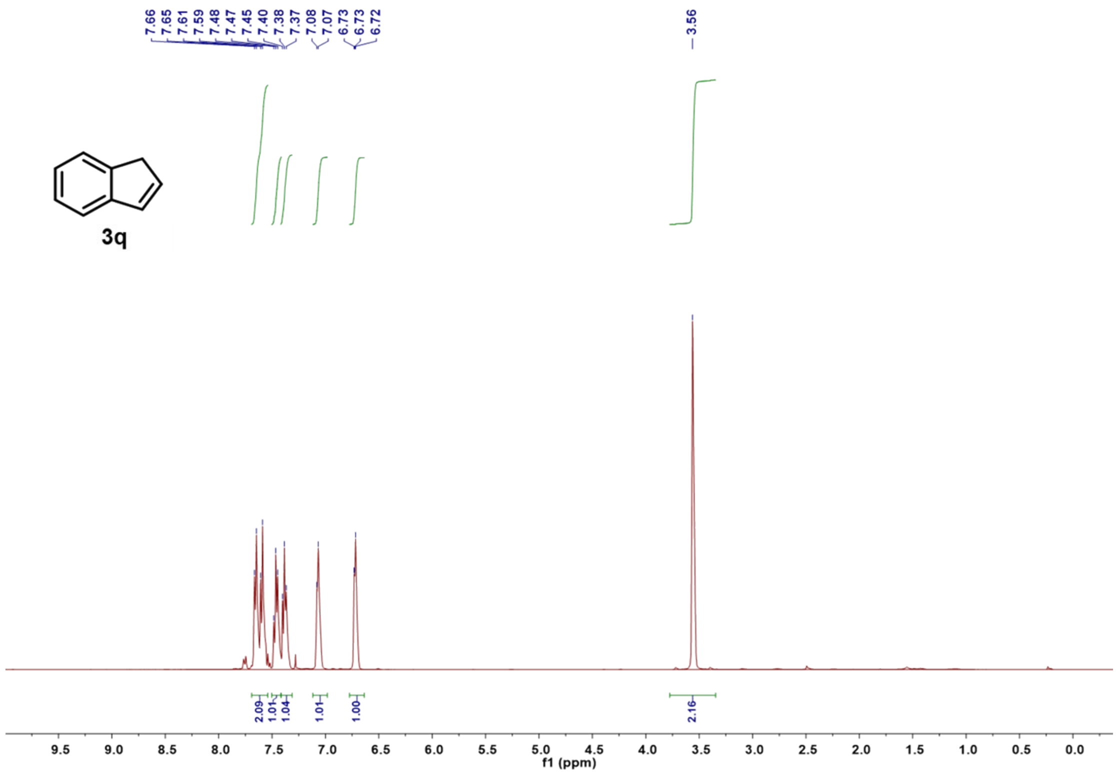
7.66
7.65
7.61
7.59
7.48
7.47
7.45
7.40
7.38
7.37
7.08
7.07
6.73
6.73
6.72
3.56
3q
2.09
1.01
1.04
1.01
1.00
2.16
9.5 9.0 8.5 8.0 7.5 7.0 6.5 6.0 5.5 5.0 4.5 4.0 3.5 3.0 2.5 2.0 1.5 1.0 0.5 0.0
f1 (ppm)

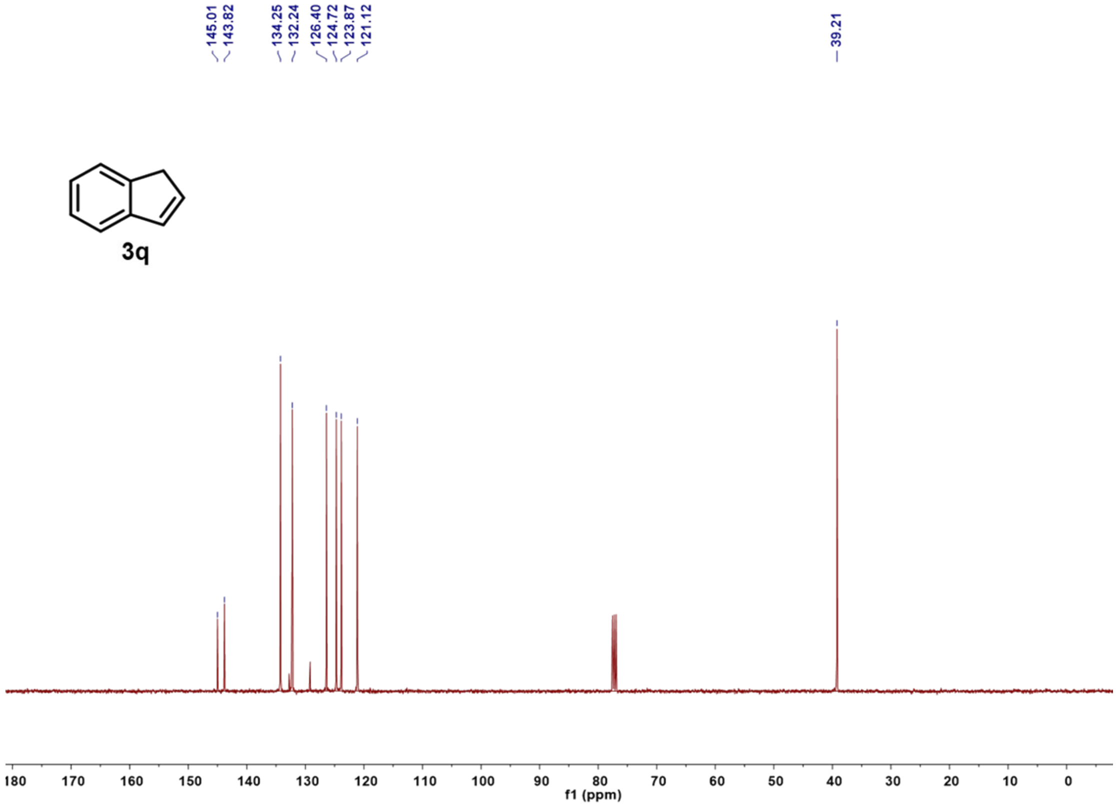
145.01
143.82
134.25
132.24
126.40
124.72
123.87
121.12
39.21
3q
180 170 160 150 140 130 120 110 100 90 80 70 60 50 40 30 20 10 0
f1 (ppm)

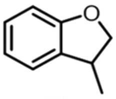

5a

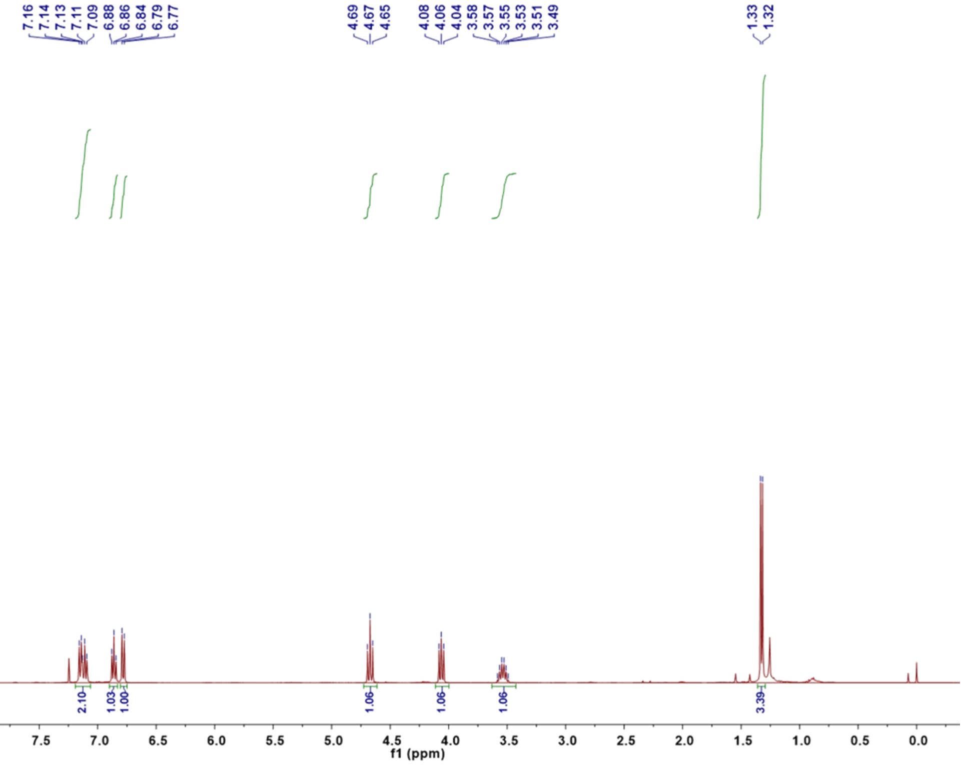
7.16
7.14
7.13
7.11
7.09
6.88
6.86
6.84
6.79
6.77
4.69
4.67
4.65
4.08
4.06
4.04
3.58
3.57
3.55
3.53
3.51
3.49
1.33
1.32
2.10
1.03
1.00
1.06
1.06
1.06
3.39
9.0 8.5 8.0 7.5 7.0 6.5 6.0 5.5 5.0 4.5 4.0 3.5 3.0 2.5 2.0 1.5 1.0 0.5 0.0
f1 (ppm)

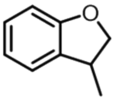

5a

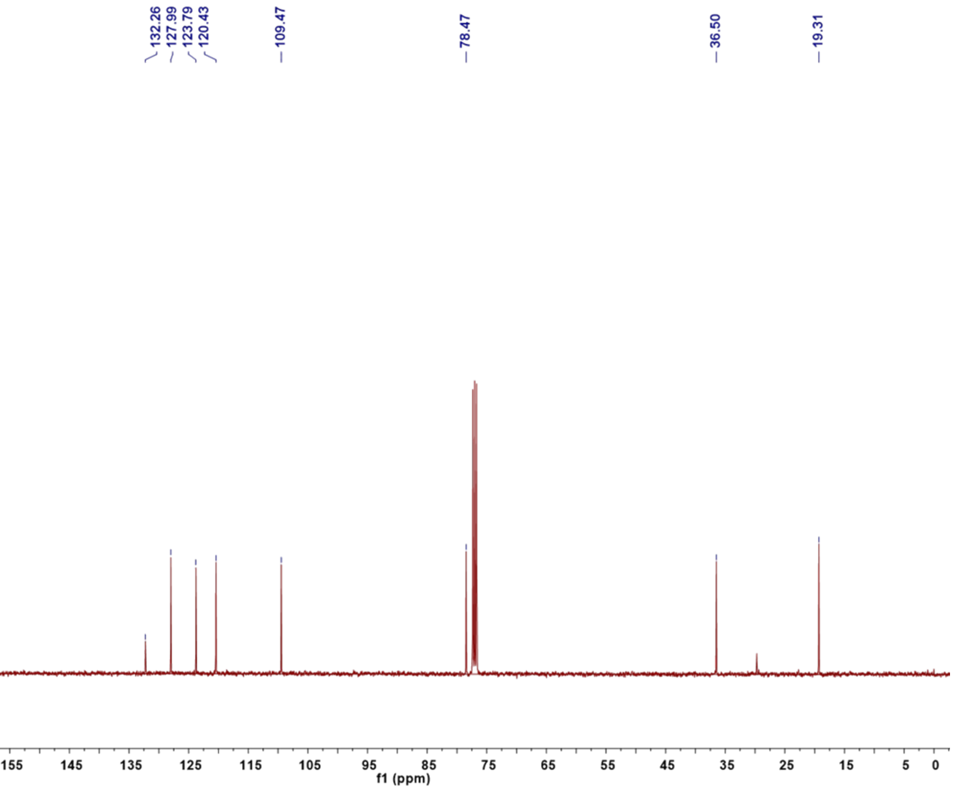
159.73
132.26
127.99
123.79
120.43
109.47
78.47
36.50
19.31
175 165 155 145 135 125 115 105 95 85 75 65 55 45 35 25 15 5 0
f1 (ppm)

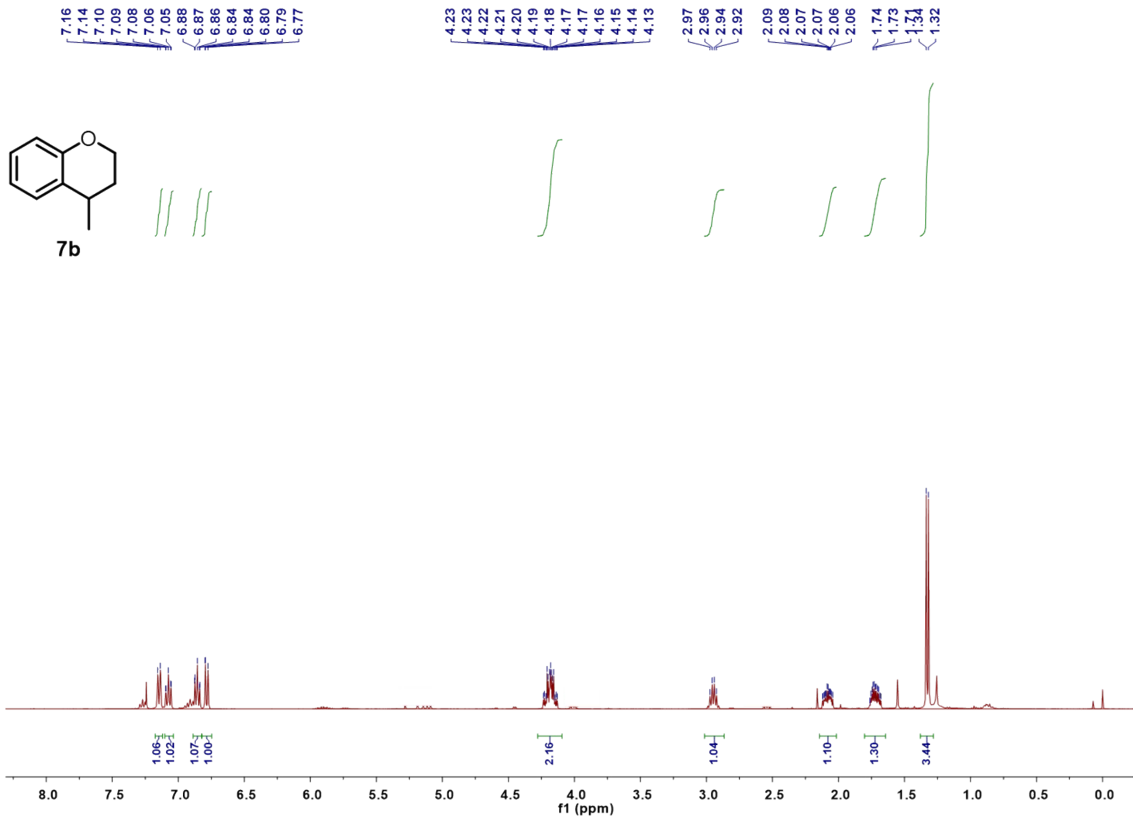
7.16 7.14 7.10 7.09 7.08 7.06 7.05 6.88 6.87 6.86 6.84 6.84 6.80 6.79 6.77
4.23 4.23 4.22 4.21 4.20 4.19 4.18 4.17 4.17 4.16 4.15 4.14 4.13
2.97 2.96 2.94 2.92
2.09 2.08 2.07 2.07 2.06 2.06
1.74 1.73 1.71 1.34 1.32
1.06 1.02 1.07 1.00 2.16 1.04 1.10 1.30 3.44
f1 (ppm)

7b

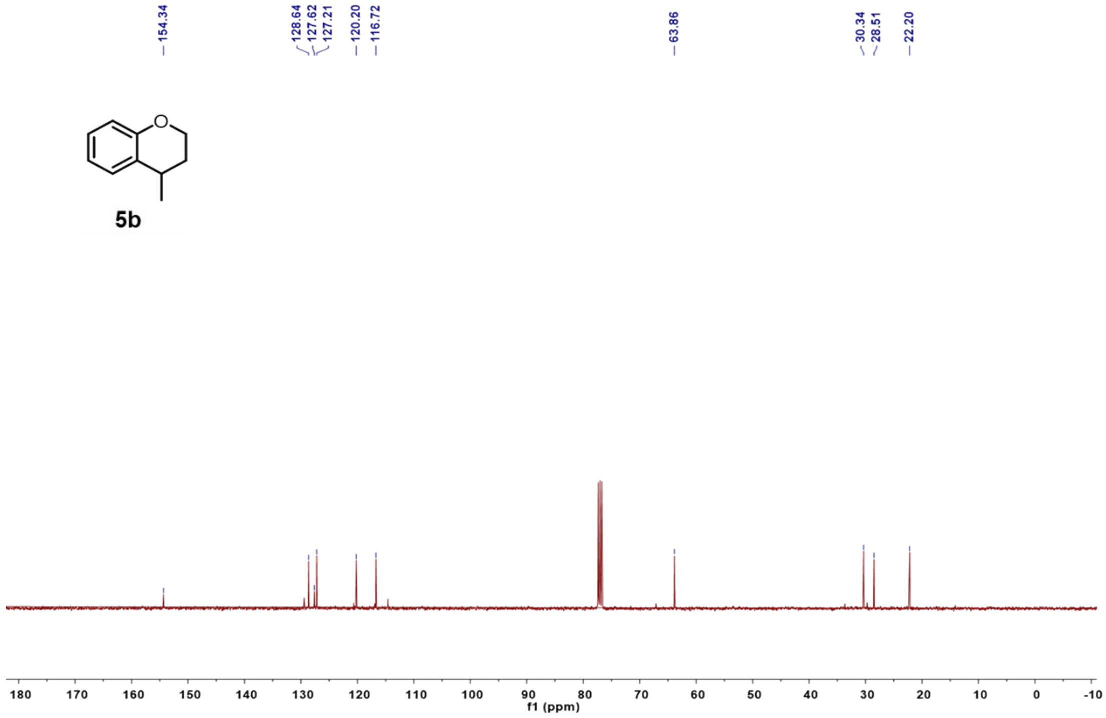
154.34
128.64 127.62 127.21 120.20 116.72
63.86
30.34 28.51 22.20
f1 (ppm)

5b

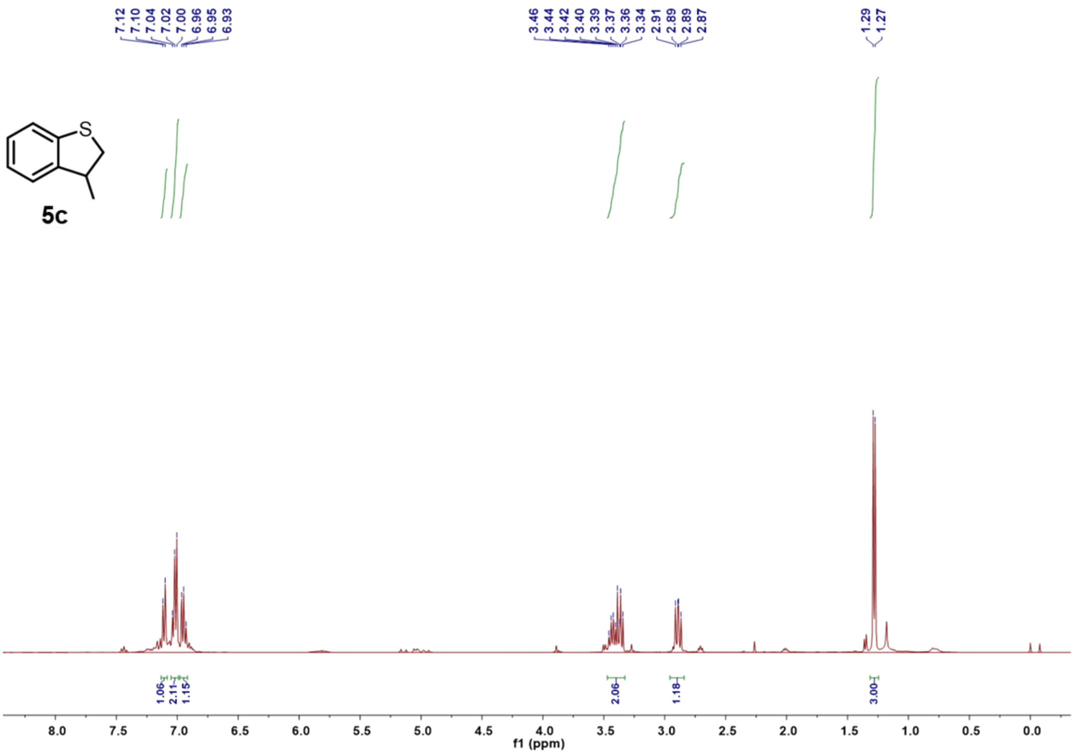
7.12
7.10
7.04
7.02
7.00
6.96
6.95
6.93
3.46
3.44
3.42
3.40
3.39
3.37
3.36
3.34
2.91
2.89
2.89
2.87
1.29
1.27
5c
1.06
2.11
1.15
2.06
1.18
3.00
f1 (ppm)

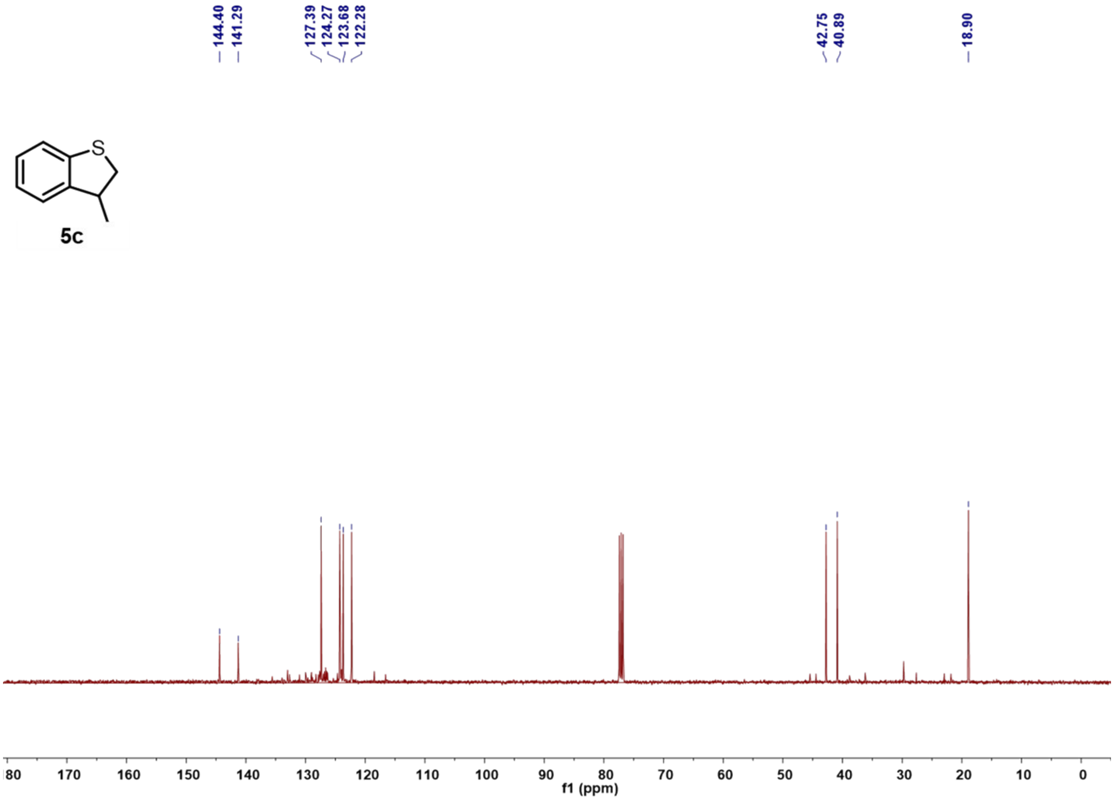
144.40
141.29
127.39
124.27
123.68
122.28
42.75
40.89
18.90
5c
f1 (ppm)

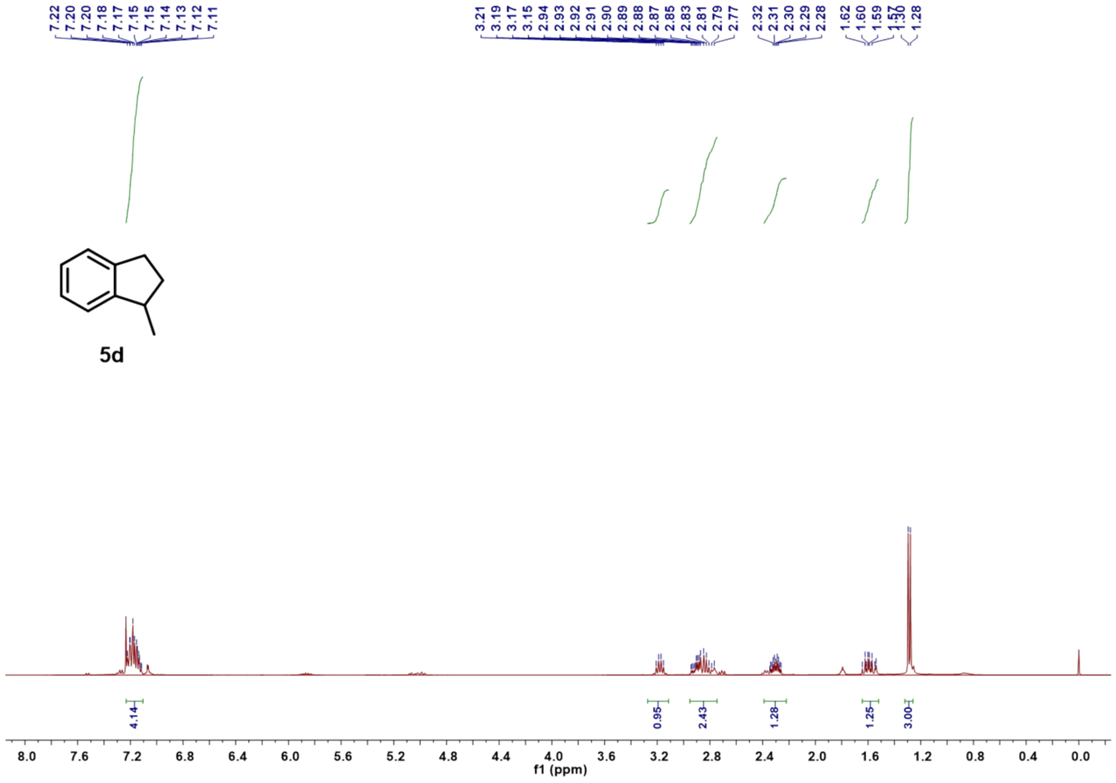
7.22
7.20
7.20
7.18
7.17
7.15
7.15
7.14
7.13
7.12
7.11
3.21
3.19
3.17
3.15
2.94
2.93
2.92
2.91
2.90
2.89
2.88
2.87
2.85
2.83
2.81
2.79
2.77
2.32
2.31
2.30
2.29
2.28
1.62
1.60
1.59
1.57
1.30
1.28
5d
4.14
0.95
2.43
1.28
1.25
3.00
8.0 7.6 7.2 6.8 6.4 6.0 5.6 5.2 4.8 4.4 4.0 3.6 3.2 2.8 2.4 2.0 1.6 1.2 0.8 0.4 0.0
f1 (ppm)

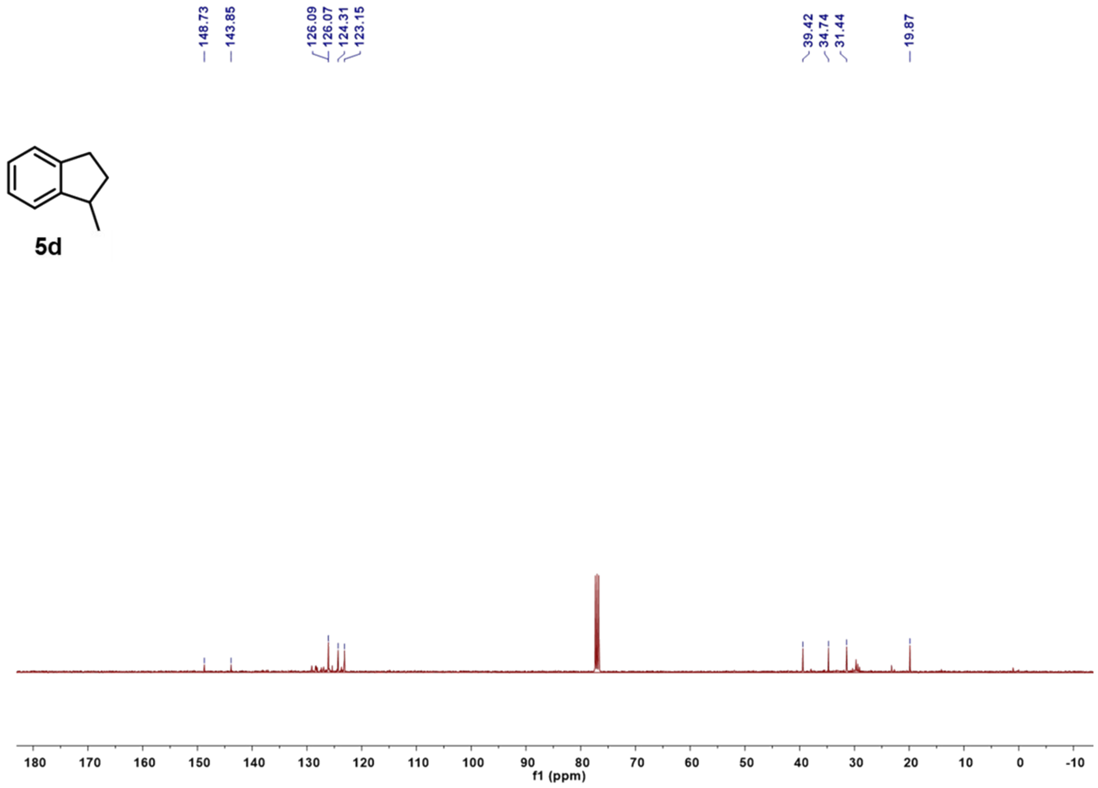
148.73
143.85
126.09
126.07
124.31
123.15
39.42
34.74
31.44
19.87
5d
180 170 160 150 140 130 120 110 100 90 80 70 60 50 40 30 20 10 0 -10
f1 (ppm)

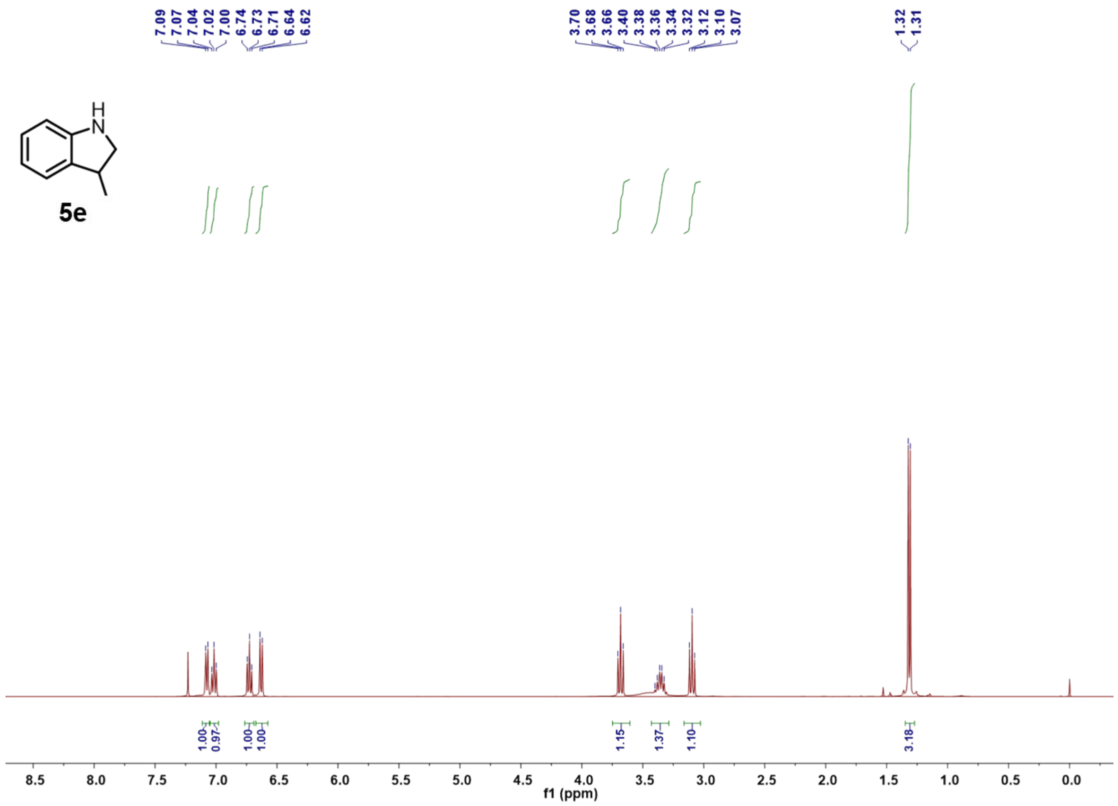
7.09
7.07
7.04
7.02
7.00
6.74
6.73
6.71
6.64
6.62
3.70
3.68
3.66
3.40
3.38
3.36
3.34
3.32
3.12
3.10
3.07
1.32
1.31
5e
1.00
0.97
1.00
1.00
1.15
1.37
1.10
3.18
8.5 8.0 7.5 7.0 6.5 6.0 5.5 5.0 4.5 4.0 3.5 3.0 2.5 2.0 1.5 1.0 0.5 0.0
f1 (ppm)

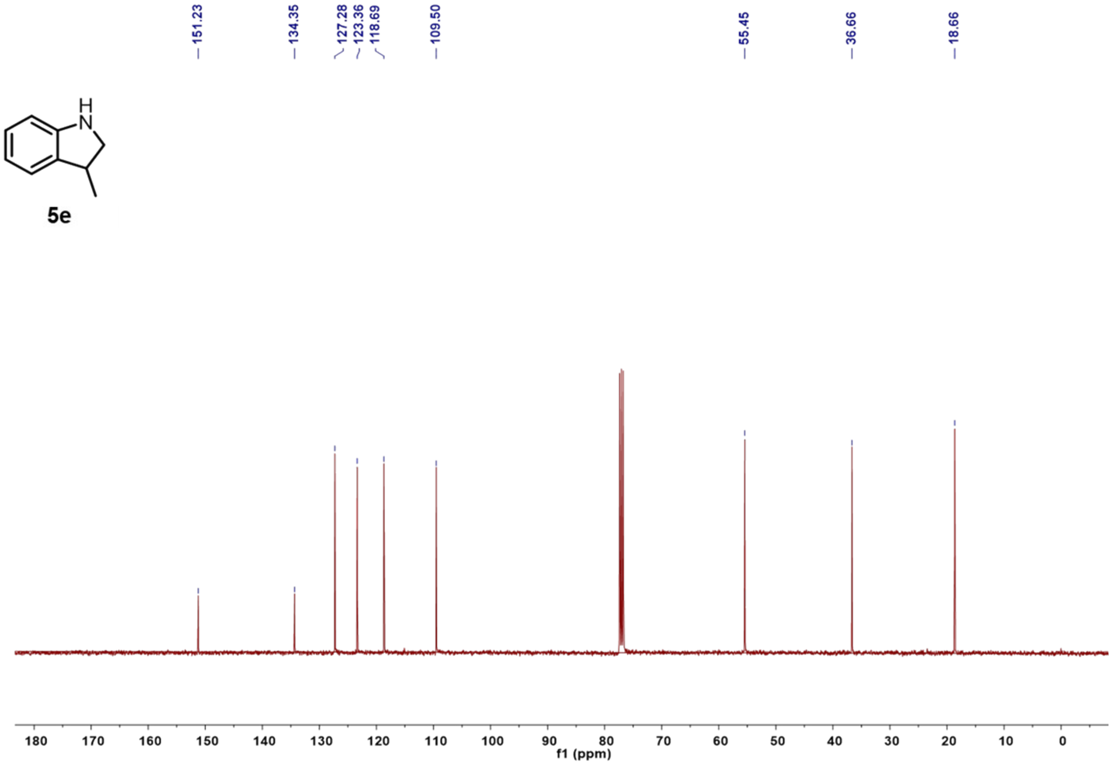
151.23
134.35
127.28
123.36
118.69
109.50
55.45
36.66
18.66
5e
180 170 160 150 140 130 120 110 100 90 80 70 60 50 40 30 20 10 0
f1 (ppm)

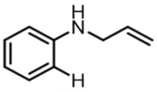

5e'

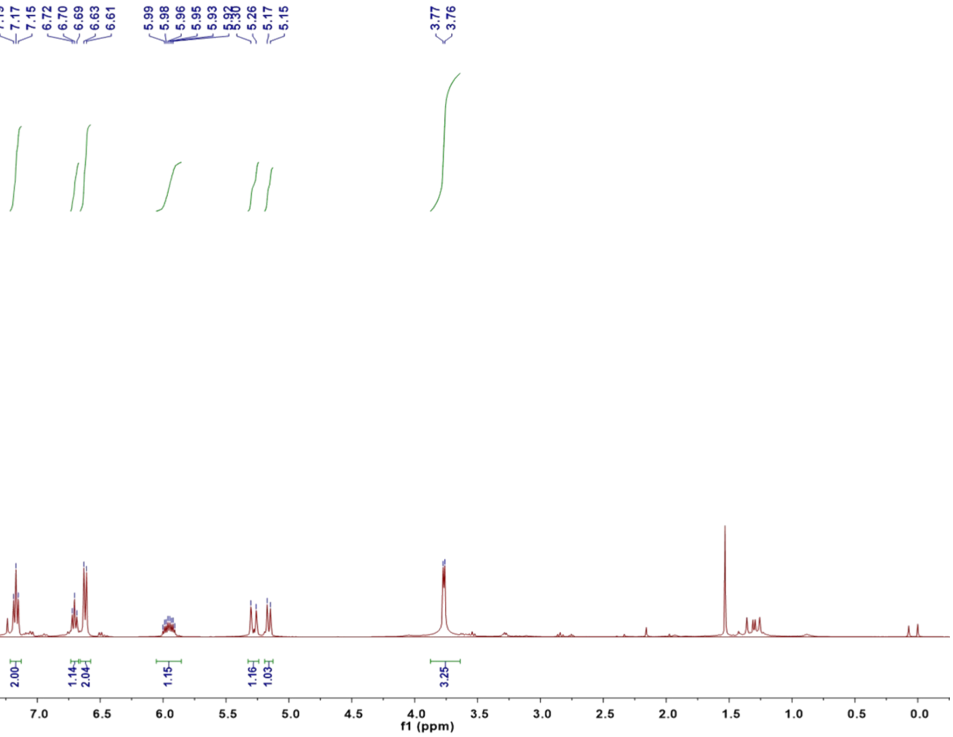
7.19
7.17
7.15
6.72
6.70
6.69
6.63
6.61
5.99
5.98
5.96
5.95
5.93
5.92
5.30
5.26
5.17
5.15
3.77
3.76
2.00
1.14
2.04
1.15
1.16
1.03
3.25
8.0 7.5 7.0 6.5 6.0 5.5 5.0 4.5 4.0 3.5 3.0 2.5 2.0 1.5 1.0 0.5 0.0
f1 (ppm)

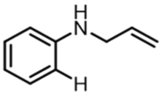

5e'

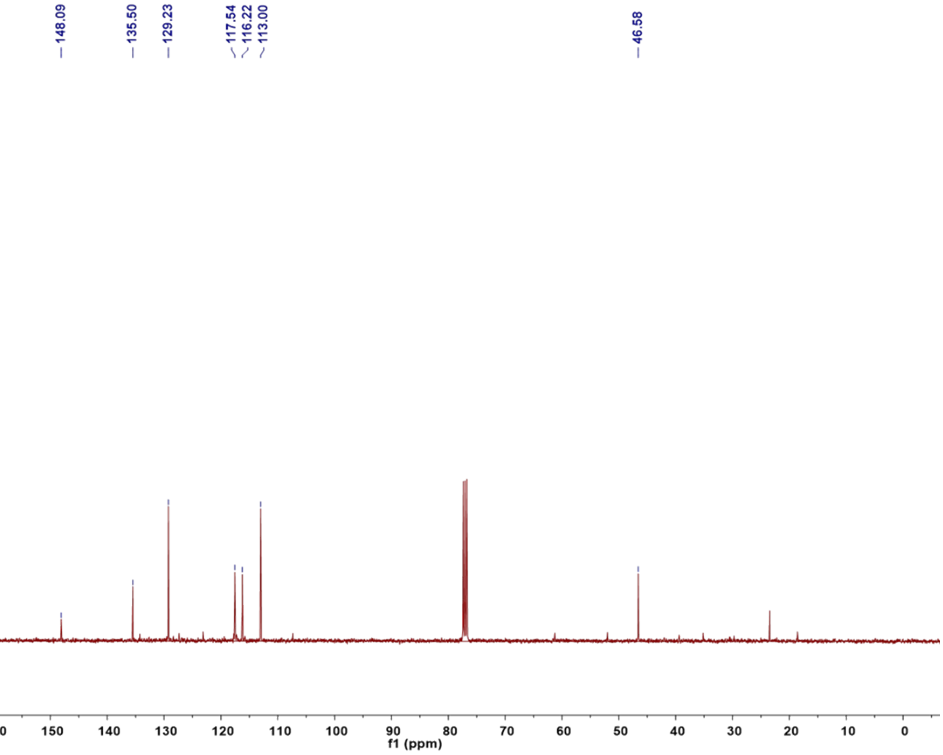
148.09
135.50
129.23
117.54
116.22
113.00
46.58
180 170 160 150 140 130 120 110 100 90 80 70 60 50 40 30 20 10 0
f1 (ppm)

11. SMD-M06-2X/6-31+G(d) Calculated Cartesian Coordinates and Energies.

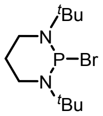

C	0.13815500	-0.82334900	2.18607300
C	-1.27395300	-0.63252400	1.62621300
H	-1.92284300	-1.43537700	1.99123300
H	-1.67798500	0.32196700	1.98567900
H	0.07128900	-1.32542800	3.15699500
C	0.99734600	-1.64084400	1.23221200
H	0.50975600	-2.59882200	1.00468300
H	1.95865400	-1.86321200	1.69771900
H	0.62306200	0.14505100	2.34559300
P	-0.02938900	-0.08509800	-0.75494100
N	1.22885000	-0.90816700	-0.01801900
N	-1.31829000	-0.65755200	0.15327900
C	-2.69504400	-0.62222600	-0.43601700
C	-3.33913300	-1.99889400	-0.23104500
C	-3.54689300	0.46954900	0.22623300
C	-2.64429300	-0.33951100	-1.94087500
H	-2.75164400	-2.77318100	-0.73618300
H	-3.42229500	-2.26371700	0.82731800
H	-4.35178900	-2.00263900	-0.64946200
H	-3.03577400	1.43767500	0.18293300
H	-4.50205500	0.55806600	-0.30238400
H	-3.77400600	0.24039300	1.27194900
H	-3.66203700	-0.40341700	-2.33868300
H	-2.26366400	0.66488600	-2.15713500
H	-2.02925600	-1.07035200	-2.47703500
C	2.63966500	-0.64916600	-0.42003100
C	2.68726700	0.09524800	-1.75920300
C	3.34176700	0.20709100	0.64313000
C	3.35522500	-1.99381700	-0.59576600
H	2.18294700	-0.46535000	-2.55359200
H	2.24760000	1.09620600	-1.69472100
H	3.73594300	0.21817900	-2.04918900
H	3.37533700	-0.29411700	1.61650900
H	4.37663100	0.40348700	0.34035600
H	2.82652400	1.16516000	0.76200400
H	4.38796400	-1.82516500	-0.91962200
H	3.39525600	-2.57179900	0.33272900
H	2.84816600	-2.59815700	-1.35594600
Br	0.11315700	2.20133800	0.19742900

Zero-point correction= 0.349784 (Hartree/Particle)

Thermal correction to Energy=	0.368050
Thermal correction to Enthalpy=	0.368994
Thermal correction to Gibbs Free Energy=	0.304532
Sum of electronic and zero-point Energies=	-3455.717547
Sum of electronic and thermal Energies=	-3455.699282
Sum of electronic and thermal Enthalpies=	-3455.698338
Sum of electronic and thermal Free Energies=	-3455.762799

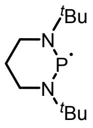

C	-0.16571600	2.17598100	0.37143100
C	1.26903400	1.66958100	0.24320100
H	1.75889000	2.22174500	-0.56696600
H	1.82293600	1.88199200	1.17255500
H	-0.17388200	3.26044400	0.21644100
C	-1.06969400	1.48343700	-0.64697500
H	-0.61711100	1.54868400	-1.64747100
H	-2.03292900	1.99594100	-0.69307500
H	-0.56186600	1.98193900	1.37562300
P	0.01527400	-0.77561000	0.44613700
N	-1.28622700	0.07923000	-0.29164800
N	1.30911800	0.23848600	-0.09014100
C	2.66635100	-0.37546000	-0.10623000
C	3.64467200	0.56401600	-0.82335600
C	3.17567400	-0.64581300	1.31863000
C	2.62706300	-1.69038300	-0.89481000
H	3.27228100	0.82694600	-1.82034400
H	3.82970800	1.48604400	-0.26348100
H	4.60785200	0.05696600	-0.94170400
H	2.53510200	-1.37274500	1.83147600
H	4.19451000	-1.05000900	1.29453800
H	3.19315300	0.27255500	1.91628500
H	3.64515000	-2.07617100	-1.01732500
H	2.03859700	-2.46140900	-0.38981400
H	2.19278300	-1.52717000	-1.88733800
C	-2.68135600	-0.35637700	-0.04606700
C	-2.72075400	-1.86177200	0.24094400
C	-3.28704500	0.39190500	1.15187700
C	-3.51385900	-0.10375200	-1.31123500
H	-2.24903600	-2.43045500	-0.56759700
H	-2.22459400	-2.11926100	1.18268000
H	-3.76488800	-2.18169300	0.32187900

H	-3.31235500	1.47481000	0.98578400
H	-4.31709100	0.06201200	1.33111700
H	-2.70063800	0.19598500	2.05705600
H	-4.53134200	-0.48559000	-1.17120000
H	-3.59574600	0.95966600	-1.55590500
H	-3.06505900	-0.61927700	-2.16718800

Zero-point correction= 0.346785 (Hartree/Particle)

Thermal correction to Energy= 0.363505

Thermal correction to Enthalpy= 0.364449

Thermal correction to Gibbs Free Energy= 0.303085

Sum of electronic and zero-point Energies= -883.837231

Sum of electronic and thermal Energies= -883.820511

Sum of electronic and thermal Enthalpies= -883.819567

Sum of electronic and thermal Free Energies= -883.880931

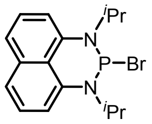

C	2.99007800	2.41605600	-0.17082000
C	3.66165300	1.22760300	-0.07714000
C	2.95386800	0.00010400	-0.16339100
C	1.54409100	0.00005100	-0.38478700
C	0.86806500	1.26609400	-0.48653500
C	1.59261000	2.43865900	-0.35783300
H	4.73739100	-1.19964600	0.07426500
H	3.52295800	3.35925800	-0.08825400
H	4.73729400	1.19998900	0.07430300
C	3.66175100	-1.22734100	-0.07717900
C	0.86815800	-1.26604100	-0.48656300
H	1.09825900	3.40124800	-0.38828000
C	1.59280100	-2.43855200	-0.35789500
C	2.99026800	-2.41584200	-0.17088700
H	1.09853300	-3.40118200	-0.38835200
H	3.52322300	-3.35900500	-0.08834700
N	-0.52240000	1.28187100	-0.71888200
N	-0.52231400	-1.28190900	-0.71889300
P	-1.49521300	-0.00005400	-0.19651400
C	-1.21012600	2.57020200	-0.97796400
C	-1.59289300	3.29541500	0.31251500
C	-2.42041700	2.37869800	-1.89149200
H	-0.48779300	3.16850100	-1.53950500
H	-0.75117700	3.36228600	1.00809600
H	-1.94101000	4.30963700	0.08777500

H	-2.40645400	2.76618100	0.82285200
H	-2.15168100	1.79536300	-2.77831900
H	-3.24978200	1.87839100	-1.38195200
H	-2.77797500	3.36010800	-2.21977500
C	-1.20997200	-2.57027200	-0.97799300
C	-2.42037100	-2.37878200	-1.89137600
C	-1.59255800	-3.29559500	0.31247800
H	-0.48766500	-3.16848600	-1.53965700
H	-2.15176700	-1.79536800	-2.77819200
H	-2.77790200	-3.36019200	-2.21968800
H	-3.24971300	-1.87856100	-1.38171100
H	-0.75076100	-3.36246500	1.00796000
H	-2.40609300	-2.76644100	0.82293900
H	-1.94064100	-4.30982100	0.08770700
Br	-1.00308800	-0.00004200	2.08284100

Zero-point correction= 0.333962 (Hartree/Particle)

Thermal correction to Energy= 0.353733

Thermal correction to Enthalpy= 0.354677

Thermal correction to Gibbs Free Energy= 0.285126

Sum of electronic and zero-point Energies= -3643.827442

Sum of electronic and thermal Energies= -3643.807671

Sum of electronic and thermal Enthalpies= -3643.806727

Sum of electronic and thermal Free Energies= -3643.876278

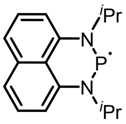

C	2.88137500	2.27275800	-0.08881800
C	3.49766900	1.05090900	-0.07218600
C	2.71999600	-0.13693100	-0.06054600
C	1.29293600	-0.06567000	-0.05911000
C	0.67201100	1.23715500	-0.08094500
C	1.47670300	2.36952800	-0.08887100
H	4.46162800	-1.41907400	-0.06776700
H	3.46897200	3.18678800	-0.09255800
H	4.58078700	0.96550600	-0.07012900
C	3.37532800	-1.39616400	-0.06943100
C	0.54515100	-1.30014500	-0.07951500
H	1.03878900	3.35847500	-0.07328400
C	1.23283500	-2.50702600	-0.08310600
C	2.64038000	-2.55051600	-0.08282000
H	0.69922300	-3.44796200	-0.06498200
H	3.13402900	-3.51866700	-0.08411600

N	-0.72621400	1.33880000	-0.10992100
N	-0.85635400	-1.26036200	-0.11263000
P	-1.73070800	0.08567600	0.50285100
C	-1.35525300	2.67125400	-0.24728900
C	-1.46008600	3.39294200	1.09823900
C	-2.71809700	2.58750100	-0.93430600
H	-0.70587100	3.23866300	-0.91995000
H	-0.49665100	3.43111300	1.61540800
H	-1.81855700	4.41875400	0.95768200
H	-2.17158000	2.87320400	1.75101200
H	-2.65937500	1.99728200	-1.85439400
H	-3.48549100	2.14840900	-0.28922500
H	-3.04325200	3.60040300	-1.19455500
C	-1.61703500	-2.52286300	-0.24814000
C	-2.95962400	-2.30323800	-0.94484500
C	-1.80210700	-3.22175800	1.10128400
H	-1.02509200	-3.15706200	-0.91390500
H	-2.83251200	-1.72775200	-1.86730400
H	-3.38673700	-3.27837300	-1.20212900
H	-3.68168100	-1.78291200	-0.30774500
H	-0.85015100	-3.34813600	1.62588700
H	-2.46431000	-2.63002000	1.74443900
H	-2.25666100	-4.20926500	0.96515000

Zero-point correction= 0.333028 (Hartree/Particle)

Thermal correction to Energy= 0.350802

Thermal correction to Enthalpy= 0.351746

Thermal correction to Gibbs Free Energy= 0.287505

Sum of electronic and zero-point Energies= -1071.950951

Sum of electronic and thermal Energies= -1071.933178

Sum of electronic and thermal Enthalpies= -1071.932233

Sum of electronic and thermal Free Energies= -1071.996475

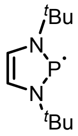

C	1.47346500	-0.18629700	0.67594500
C	1.47346500	-0.18629700	-0.67594500
H	2.35286000	-0.17079000	1.30585800
H	2.35286000	-0.17079000	-1.30585800
N	0.20327600	-0.27197800	1.22498400
N	0.20327600	-0.27197800	-1.22498400
P	-1.04581700	-0.24026300	0.00000000
C	-0.05844900	0.05766100	2.64213200

C	0.09560900	1.56947900	2.85048800
C	-1.48348100	-0.37528600	2.99353500
C	0.92285500	-0.70565300	3.53695300
H	1.11501400	1.89224100	2.61031700
H	-0.59731500	2.11621100	2.20100800
H	-0.11408000	1.83971100	3.89170400
H	-1.62485900	-1.44612500	2.81206900
H	-1.67543000	-0.17507200	4.05254700
H	-2.23121000	0.17951700	2.41471900
H	0.65855700	-0.54645800	4.58773200
H	0.88268000	-1.77986200	3.32746600
H	1.95447700	-0.36297900	3.40874100
C	-0.05844900	0.05766100	-2.64213200
C	-1.48348100	-0.37528600	-2.99353500
C	0.09560900	1.56947900	-2.85048800
C	0.92285500	-0.70565300	-3.53695300
H	-1.62485900	-1.44612500	-2.81206900
H	-2.23121000	0.17951700	-2.41471900
H	-1.67543000	-0.17507200	-4.05254700
H	1.11501400	1.89224100	-2.61031700
H	-0.11408000	1.83971100	-3.89170400
H	-0.59731500	2.11621100	-2.20100800
H	0.65855700	-0.54645800	-4.58773200
H	1.95447700	-0.36297900	-3.40874100
H	0.88268000	-1.77986200	-3.32746600

Zero-point correction= 0.293140 (Hartree/Particle)
Thermal correction to Energy= 0.307847
Thermal correction to Enthalpy= 0.308791
Thermal correction to Gibbs Free Energy= 0.252009
Sum of electronic and zero-point Energies= -843.402901
Sum of electronic and thermal Energies= -843.388194
Sum of electronic and thermal Enthalpies= -843.387250
Sum of electronic and thermal Free Energies= -843.444032

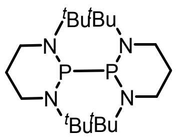

C	-1.67827800	-0.07800400	2.46838800
C	-2.00505100	1.27519700	1.84570000
H	-3.08160400	1.45393800	1.99378600
H	-1.47239000	2.06597200	2.39404600
H	-2.08744000	-0.08777900	3.48519900
C	-2.29024100	-1.23780600	1.69367800

H	-3.38140300	-1.07129000	1.62479000
H	-2.13990100	-2.15922600	2.26858200
H	-0.59648100	-0.21743300	2.55990000
P	-1.04413500	-0.00287400	-0.43193800
N	-1.70418300	-1.39870400	0.36081400
N	-1.69571200	1.34389400	0.41463800
C	-2.39271900	2.41969100	-0.34278900
C	-2.35566100	3.72159700	0.47006700
C	-1.67118000	2.69135100	-1.66786400
C	-3.85029200	2.03055400	-0.62774300
H	-2.92242500	3.65269000	1.40362900
H	-1.32271500	3.99826000	0.71169400
H	-2.79610300	4.53271300	-0.11951200
H	-1.75614300	1.85520500	-2.36774700
H	-2.10514600	3.57683100	-2.14615800
H	-0.60627900	2.87742500	-1.48459700
H	-4.37877000	2.83857800	-1.14816000
H	-3.88729300	1.13671500	-1.25996800
H	-4.39709000	1.81677900	0.29829200
C	-2.36418600	-2.41571400	-0.50745100
C	-1.39464300	-2.84773900	-1.61345600
C	-2.69889900	-3.66381400	0.32094300
C	-3.65291000	-1.86935700	-1.14054200
H	-1.12833100	-2.01394900	-2.27111500
H	-0.47060800	-3.24255200	-1.17597700
H	-1.85792700	-3.62628700	-2.23076400
H	-3.49867700	-3.48553800	1.04689700
H	-3.03897600	-4.45998600	-0.34976100
H	-1.81445700	-4.02651700	0.85739700
H	-4.18955300	-2.65970500	-1.67909500
H	-4.32866400	-1.46341300	-0.37833700
H	-3.42047800	-1.07256800	-1.85567700
C	1.67828100	-0.07741400	-2.46837600
C	2.00456200	1.27592500	-1.84573100
H	3.08104400	1.45508400	-1.99381200
H	1.47160100	2.06645800	-2.39413500
H	2.08746600	-0.08707500	-3.48518100
C	2.29066500	-1.23702100	-1.69369500
H	3.38179800	-1.07022400	-1.62504700
H	2.14043600	-2.15851500	-2.26851900
H	0.59653000	-0.21721200	-2.55992000
P	1.04419500	-0.00244900	0.43190900
N	1.70494400	-1.39797400	-0.36069700
N	1.69516200	1.34459600	-0.41467000

C	2.39164300	2.42075800	0.34275600
C	2.35414300	3.72261200	-0.47017200
C	1.66974100	2.69215400	1.66768400
C	3.84931900	2.03228100	0.62800900
H	2.92105800	3.65390100	-1.40365500
H	1.32110900	3.99881600	-0.71194700
H	2.79416200	4.53395200	0.11941800
H	1.75490200	1.85604800	2.36759500
H	2.10323600	3.57782600	2.14604400
H	0.60479800	2.87777200	1.48419700
H	4.37730800	2.84056000	1.14853500
H	3.88660100	1.13849500	1.26028900
H	4.39641800	1.81874000	-0.29789700
C	2.36529600	-2.41479500	0.50750600
C	1.39597400	-2.84710200	1.61357900
C	2.70029100	-3.66274000	-0.32098900
C	3.65393500	-1.86811000	1.14050500
H	1.12964400	-2.01340400	2.27135200
H	0.47193900	-3.24203600	1.17621900
H	1.85948500	-3.62560500	2.23078300
H	3.49989300	-3.48412500	-1.04705200
H	3.04074100	-4.45882000	0.34963600
H	1.81589100	-4.02571100	-0.85733400
H	4.19087900	-2.65832100	1.67895300
H	4.32945800	-1.46189900	0.37823200
H	3.42134400	-1.07143200	1.85571200

Zero-point correction= 0.696545 (Hartree/Particle)

Thermal correction to Energy= 0.730468

Thermal correction to Enthalpy= 0.731412

Thermal correction to Gibbs Free Energy= 0.635764

Sum of electronic and zero-point Energies= -1767.745395

Sum of electronic and thermal Energies= -1767.711472

Sum of electronic and thermal Enthalpies= -1767.710528

Sum of electronic and thermal Free Energies= -1767.806176

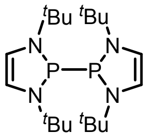

C	-2.18041300	-0.68269100	1.59392900
C	-2.18936100	0.66178300	1.58995200
H	-2.47573500	-1.29876300	2.43562300
H	-2.49475800	1.27914200	2.42726000
N	-1.67637400	-1.28996600	0.41686900

N	-1.69568600	1.26831500	0.40894600
P	-0.98120900	-0.00793100	-0.55362500
C	-2.44460100	-2.40717400	-0.19939000
C	-3.74708600	-1.89902200	-0.82681800
C	-1.56803300	-3.06060500	-1.26817200
C	-2.76125100	-3.44394100	0.88270100
H	-4.33408000	-1.33584300	-0.09170800
H	-3.53458300	-1.24345000	-1.67917600
H	-4.35936100	-2.73467000	-1.18646700
H	-0.65101800	-3.46035600	-0.82287900
H	-2.11215300	-3.88159100	-1.74849500
H	-1.28058300	-2.34545800	-2.04691000
H	-3.16840300	-4.34742800	0.41645400
H	-1.85479700	-3.71972400	1.43327300
H	-3.50786200	-3.08077000	1.59649200
C	-2.47135500	2.38370300	-0.20008800
C	-1.64279300	2.98610800	-1.33500700
C	-3.81767000	1.88907500	-0.74020900
C	-2.70517500	3.45654000	0.86864000
H	-0.67271300	3.33390000	-0.96412600
H	-1.45797300	2.25627300	-2.13073300
H	-2.17620100	3.83569200	-1.77571300
H	-4.39252300	1.39296900	0.05069800
H	-4.41472200	2.72523100	-1.12347500
H	-3.66772200	1.17577800	-1.55873000
H	-3.12881100	4.35360300	0.40443700
H	-3.41128800	3.12439500	1.63668100
H	-1.76218100	3.73050400	1.35467900
C	2.18887500	-0.66153300	-1.59021000
C	2.18010700	0.68294700	-1.59375300
H	2.49394000	-1.27869700	-2.42778500
H	2.47543800	1.29924100	-2.43528600
N	1.69537700	-1.26836700	-0.40922300
N	1.67645800	1.28987800	-0.41640500
P	0.98111600	0.00775400	0.55375000
C	2.47155600	-2.38360000	0.19956800
C	3.81787800	-1.88866200	0.73940500
C	1.64349000	-2.98631800	1.33467900
C	2.70527600	-3.45633700	-0.86927800
H	4.39246500	-1.39245400	-0.05162100
H	3.66792200	-1.17539400	1.55795600
H	4.41518900	-2.72467700	1.12257400
H	0.67334700	-3.33419100	0.96406100
H	2.17718200	-3.83589900	1.77504500

H	1.45878300	-2.25663800	2.13056500
H	3.12930900	-4.35331100	-0.40526000
H	1.76216400	-3.73052700	-1.35497200
H	3.41103300	-3.12401000	-1.63755900
C	2.44455500	2.40705900	0.19987700
C	1.56811500	3.06008900	1.26900600
C	3.74728100	1.89897000	0.82689300
C	2.76082200	3.44409500	-0.88207400
H	0.65088600	3.45967400	0.82399200
H	1.28104200	2.34472900	2.04768700
H	2.11216300	3.88110500	1.74935000
H	4.33413300	1.33597000	0.09151900
H	4.35955800	2.73461600	1.18653600
H	3.53508600	1.24319000	1.67916400
H	3.16797000	4.34753700	-0.41573600
H	3.50730200	3.08114800	-1.59612600
H	1.85421000	3.71988200	-1.43238400

Zero-point correction= 0.589097 (Hartree/Particle)

Thermal correction to Energy= 0.620387

Thermal correction to Enthalpy= 0.621332

Thermal correction to Gibbs Free Energy= 0.530344

Sum of electronic and zero-point Energies= -1686.852754

Sum of electronic and thermal Energies= -1686.821464

Sum of electronic and thermal Enthalpies= -1686.820519

Sum of electronic and thermal Free Energies= -1686.911507

12. Reference.

1. (a) J. Barluenga, F. J. Fañanás, R. Sanz, C. Marcos and M. Trabada, *Org. Lett.*, 2002, **4**, 1587-1590; (b) V. Pace, L. Castoldi and W. Holzer, *Tetrahedron Lett.*, 2012, **53**, 967-972; (c) G. A. Molander and D. L. Sandrock, *J. Am. Chem. Soc.*, 2008, **130**, 15792-15793; (d) S. Xu, F. Haeffner, B. Li, L. N. Zakharov and S.-Y. Liu, *Angew. Chem. Int. Ed.*, 2014, **53**, 6795-6799.
2. J. Zhang, J.-D. Yang and J.-P. Cheng, *Angew. Chem. Int. Ed.*, 2019, **58**, 5983-5987.
3. J. Zhang, J.-D. Yang and J.-P. Cheng, *Chem. Sci.*, 2020, DOI: 10.1039/C9SC05883D.
4. (a) H. A. Spinney, I. Korobkov, G. A. DiLabio, G. P. A. Yap and D. S. Richeson, *Organometallics*, 2007, **26**, 4972-4982; (b) H. A. Spinney, G. P. A. Yap, I. Korobkov, G. DiLabio and D. S. Richeson, *Organometallics*, 2006, **25**, 3541-3543.
5. R. Matsubara, T. Yabuta, U. Md Idros, M. Hayashi, F. Ema, Y. Kobori and K. Sakata, *J. Org. Chem.*, 2018, **83**, 9381-9390.
6. H. Kim and C. Lee, *Angew. Chem. Int. Ed.*, 2012, **51**, 12303-12306.
7. S. Urban, B. Beiring, N. Ortega, D. Paul and F. Glorius, *J. Am. Chem. Soc.*, 2012, **134**, 15241-15244.
8. C. Santilli, S. S. Beigbaghlou, A. Ahlburg, G. Antonacci, P. Fristrup, P.-O. Norrby and R. Madsen, *Eur. J. Org. Chem.*, 2017, **2017**, 5269-5274.
9. M. Rauser, R. Eckert, M. Gerbershagen and M. Niggemann, *Angew. Chem. Int. Ed.*, 2019, **58**, 6713-6717.

10. F. Ding, Y. Zhang, R. Zhao, Y. Jiang, R. L.-Y. Bao, K. Lin and L. Shi, *Chem. Commun.*, 2017, **53**, 9262-9264.
11. M. J. T. Frisch, G. W.; Schlegel, H. B.; Scuseria, G. E.;Robb, M. A.; Cheeseman, J. R.; Scalmani, G.; Barone, V.; Mennucci,B.; Petersson, G. A.; Nakatsuji, H.; Caricato, M.; Li, X.; Hratchian, H.P.; Izmaylov, A. F.; Bloino, J.; Zheng, G.; Sonnenberg, J. L.; Hada, M.;Ehara, M.; Toyota, K.; Fukuda, R.; Hasegawa, J.; Ishida, M.; Nakajima,T.; Honda, Y.; Kitao, O.; Nakai, H.; Vreven, T.; Montgomery, J. A., Jr.;J. E, P.; Ogliaro, F.; Bearpark, M.; Heyd, J. J.; Brothers, E.; Kudin, K.N.; Staroverov, V. N.; Keith, T.; Kobayashi, R.; Normand, J.;Raghavachari, K.; Rendell, A.; Burant, J. C.; Iyengar, S. S.; Tomasi,J.; Cossi, M.; Rega, N.; Millam, J. M.; Klene, M.; Knox, J. E.; Cross, J.B.; Bakken, V.; Adamo, C.; Jaramillo, J.; Gomperts, R.; Stratmann, R.E.; Yazyev, O.; Austin, A. J.; Cammi, R.; Pomelli, C.; Ochterski, J. W.;Martin, R. L.; Morokuma, K.; Zakrzewski, V. G.; Voth, G. A.; Salvador,P.; Dannenberg, J. J.; Dapprich, S.; Daniels, A. D.; Farkas, O.;Foresman, J. B.; Ortiz, J. V.; Cioslowski, J.; Fox, D. J.Gaussian 09,Revision D.01, Gaussian, Inc., Wallingford, CT, 2013.
12. (a) Y. Zhao and D. G. Truhlar, *Acc. Chem. Res.*, 2008, **41**, 157-167; (b) Y. Zhao and D. G. Truhlar, *Chem. Phys. Lett.*, 2011, **502**, 1-13.
13. A. V. Marenich, C. J. Cramer and D. G. Truhlar, *J. Phys. Chem. B*, 2009, **113**, 6378-6396.
14. R. Edge, R. J. Less, E. J. L. McInnes, K. Müther, V. Naseri, J. M. Rawson and D. S. Wright, *Chem. Commun.*, 2009, 1691-1693.
